# Oxindole Compounds Induce NRF2-ARE Antioxidant Pathway in MCF7 Cells and Reduce Inflammatory Response in RAW264.7 Cells

**DOI:** 10.3390/molecules31183314

**Published:** 2026-09-18

**Authors:** Baskar Selvaraj, Qui Ngoc Sang Nguyen, Seong-Hee Ko, Ki-Yeon Yoo, Suk Woo Kang, Jae Wook Lee

**Affiliations:** 1 Center for Natural Product Efficacy Optimization, Gangneung Institute, Korea Institute of Science and Technology (KIST), Gangneung 25451, Republic of Koreakevindh2515@kist.re.kr (Q.N.S.N.); 2 Department of Pharmacology, College of Dentistry, Gangwon National University, Gangneung 25457, Republic of Korea; 3 Department of Anatomy, College of Dentistry, Gangwon National University, Gangneung 25457, Republic of Korea; 4 Institute of Chemistry, Vietnam Academy of Science and Technology (VAST), Hanoi 100000, Vietnam; 5 Natural Product Applied Science, KIST School, University of Science and Technology, Gangneung 25451, Republic of Korea

**Keywords:** oxindole compounds, NRF2–ARE signaling pathway, antioxidant activity, anti-inflammatory activity, oxidative stress, KWAP1, PH-1, NQO1, EAW264.7 macrophages

## Abstract

Using previously reported chemical structures and synthetic methods, we prepared a series of 19 oxindole derivatives and evaluated their ability to activate the NRF2–ARE signaling pathway and induce the expression of downstream antioxidant genes. All 19 compounds activated NRF2–ARE signaling. Among them, OIC3 and OIC15 were selected for further investigation based on their relatively low EC_50_ values of 6.20 and 4.95 µM, respectively, as well as their favorable cytotoxicity profiles. Both compounds activated NRF2 in a concentration-dependent manner and progressively increased the expression of the NRF2 target genes HO-1 and NQO1 over 8–24 h. Their anti-inflammatory activities were further evaluated in RAW 264.7 macrophages. Quantum chemical calculations identified the exocyclic benzylidene carbon as the most probable electrophilic reaction site, while covalent docking suggested that the Cys151 adducts of both compounds could be structurally accommodated within the KEAP1 BTB domain. Collectively, these findings identify OIC3 and OIC15 as promising NRF2–ARE activators and support their further investigation as potential therapeutic candidates for oxidative stress- and inflammation-related diseases.

## 1. Introduction

The nuclear factor erythroid 2-related factor 2 (NRF2) antioxidant response element (ARE) signaling pathway (NRF2-ARE) serves as a critical cellular defense mechanism against intracellular stress induced by reactive oxygen species (ROS) and other oxidative stressors that cause cellular and tissue damage [1,2]. The NRF2-ARE signaling regulates the expression of various cytoprotective genes that attenuate highly reactive, electrophilic, and oxidative stressors.

Under unstressed conditions, NRF2 is continuously synthesized, and its intracellular levels are restricted in the cytoplasm by Kelch-like ECH-associated protein 1 (KEAP1), which binds NRF2 and delivers it to the CUL3-based E3 ubiquitin ligase complex for K48-linked ubiquitination; the polyubiquitinated NRF2 is subsequently degraded by the 26S proteasome complex, thereby controlling intracellular NRF2 levels [3,4,5]. When cells are exposed to excessive oxidative stress, reactive oxygen species (ROS) and electrophiles modify critical cysteine residues of KEAP1 (such as Cys151, Cys273, and Cys288), leading to conformational changes that impair NRF2-KEAP1 binding, thereby allowing NRF2 to evade proteasomal degradation [6,7]. This allows newly synthesized NRF2 to accumulate and translocate the nucleus, where it heterodimerizes with small Maf proteins and binds to AREs in the promoters of target genes, thereby inducing transcription of cytoprotective and detoxification genes such as heme oxygenase-1 (HO-1) and NAD(P)H quinone oxidoreductase 1 (NQO1) [8,9]. This NRF2-ARE signaling pathway is essential for protecting cells from oxidative damage, and has emerged as a promising target for therapeutic intervention in diseases associated with oxidative stress.

A wide range of drugs and small molecules activate the NRF2-ARE pathway, thereby inducing antioxidant and cytoprotective gene expression and offering therapeutic potential for diseases associated with excessive oxidative stress-mediated damage [10,11]. The FDA-approved NRF2 activator dimethyl fumarate (DMF) is used to treat multiple sclerosis relapses by stabilizing NRF2 via KEAP1 cysteine modification and inducing its target genes, HO-1 and NQO1 [10,12]. Omaveloxolone (RTA 408), a synthetic antioxidant, activates NRF2 and is approved for the treatment of Friedreich’s ataxia, highlighting the clinical relevance of targeted NRF2 activation [13,14]. Other synthetic compounds such as bardoxolone methyl (CDDO-Me) have been investigated for chronic kidney disease and inflammatory conditions due to their potent NRF2 induction [15,16]. Natural compounds such as sulforaphane, curcumin, resveratrol, *trans*-chalcone, and andrographolide have demonstrated NRF2 activation and antioxidant effects in preclinical studies and some are being explored for chemoprevention and chronic disease modulation [17,18,19]. Despite these advances, many activators exhibit limited bioavailability, off-target effects, or safety concerns, highlighting the need for novel, selective NRF2 modulators with improved therapeutic indices for clinical application.

Indole-based compounds have the ability to induce the NRF2-ARE antioxidant pathway and reduce oxidative stress, making them promising leads for therapeutic development. Several studies have demonstrated that indole derivatives can enhance the NRF2-ARE pathway and activate target antioxidant genes in in vitro and in vivo animal models [20,21]. Natural indole compounds, such as indole-3-carbinol from cruciferous vegetables, asperpenazine and asperpendoline from fungi, and indole-3-acetaldehyde and indole acetic acid from the microbiome, have been shown to activate NRF2-ARE and upregulate antioxidant genes [22,23,24]. Oxindole and indole-based compounds represent an important and versatile chemical scaffold for the modulation of the NRF2-ARE signaling pathway due to their favorable redox-responsive properties and structural flexibility [23,25]. These properties highlight their therapeutic potential in diseases driven by oxidative stress, including neurodegenerative disorders, inflammatory conditions, and metabolic diseases [26,27,28]. However, many reported indole-based NRF2 activators exhibit limitations such as cytotoxicity at higher concentrations, insufficient selectivity, or suboptimal pharmacokinetic profiles. Therefore, the continued discovery and optimization of novel oxindole derivatives with improved potency, safety, and NRF2 pathway selectivity are essential to advance these compounds toward clinically viable antioxidant and cytoprotective therapeutics.

Herein, we synthesized 19 oxindole derivatives and studied their potential for NRF2-ARE activation and downstream target gene activation. We selected two compounds, OIC3 and OIC15, which significantly induced the NRF2 pathway at 6.22 µM and 4.95 µM, respectively, and increased HO-1 and NQO1 protein levels, demonstrating therapeutic potential against oxidative stress-mediated diseases.

## 2. Results and Discussion

### 2.1. Synthesis of Oxindole Derivatives

**Figure 1:**
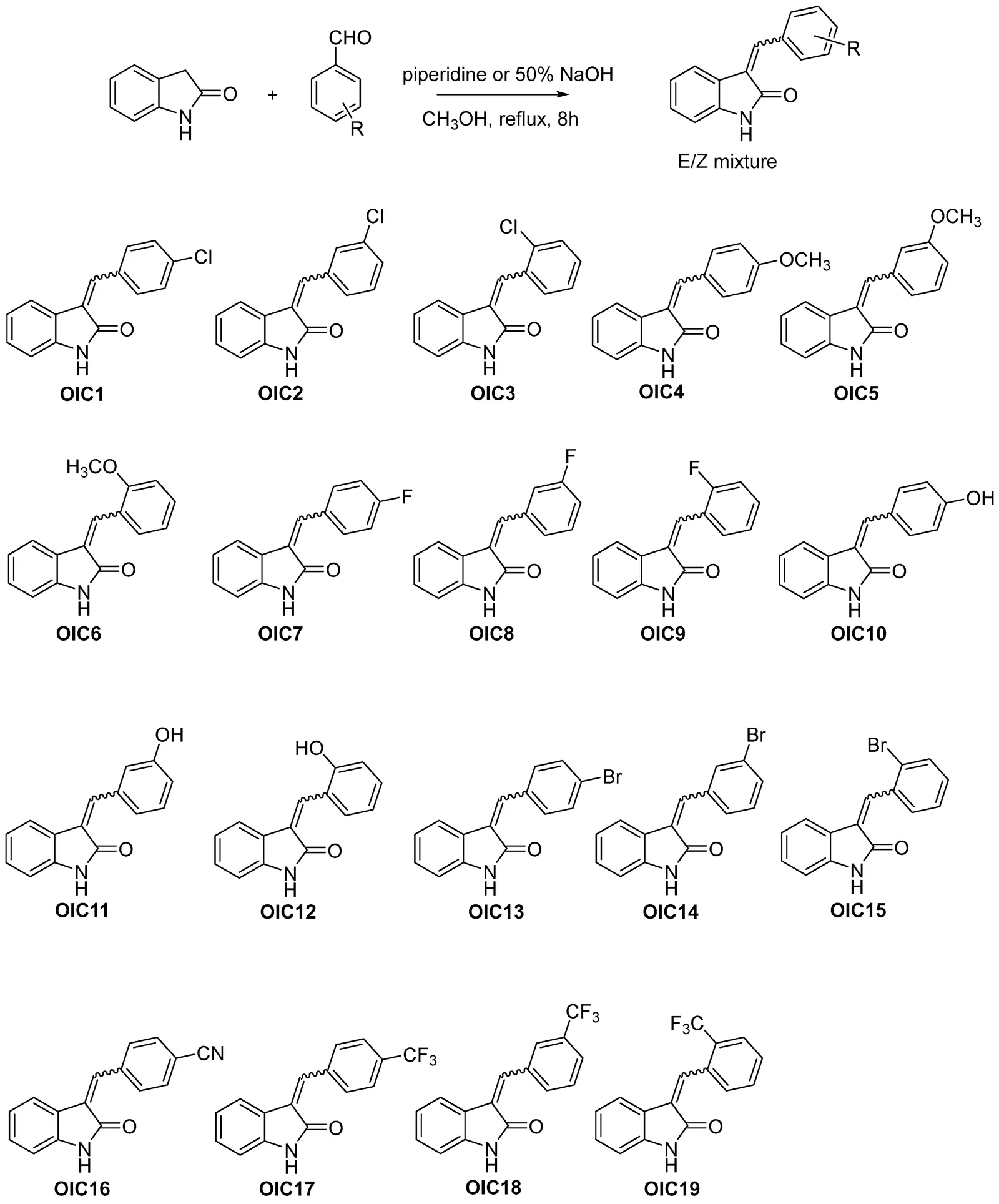
The synthetic scheme and chemical structures of the oxindole derivatives OIC1–OIC19 evaluated in this study. The compounds were prepared based on previously reported structures and synthetic methods [29,30,33,34,35,36,37,38,39].

As shown in Figure 1, a series of 19 oxindole derivatives was synthesized via Knoevenagel condensation using indolin-2-one as the starting material [29,30]. Nineteen aryl aldehydes bearing various substituents were selected to investigate the effects of their electronic properties on biological activity. Methoxy and hydroxy groups were incorporated as electron-donating substituents, whereas cyano, fluoro, chloro, bromo, and trifluoromethyl groups were introduced as electron-withdrawing substituents. Following completion of the reactions, the products were purified by silica gel column chromatography or preparative HPLC, affording the desired derivatives in yields ranging from 23% to 37%.

The α,β-unsaturated keto moiety present in the oxindole scaffold may act as a reactive electrophilic site capable of interacting with cysteine residues in KEAP1 [31]. Therefore, the electronic properties of the substituents on the aryl aldehydes may modulate the electrophilicity of the α,β-unsaturated keto moiety and consequently influence the biological activity of the resulting oxindole derivatives [32]. Based on this rationale, a series of 19 compounds with diverse electronic properties was synthesized to investigate the structure–activity relationship of these derivatives.

### 2.2. NRF2-ARE Activation of Oxindole Compounds and Hit Selection

The ARE-LUC activation was tested in ARE-LUC-encoded MCF7 reporter cells. The 19 oxindole compounds were treated in ARE-MCF7 cells using a two-fold serial dilution from 100 µM to 3.1 µM, and the luciferase signal was measured using the ONE-Glo reagent, and EC_50_ graphs were generated (Figure S1).

All synthesized compounds induced NRF2-ARE luciferase signals at the tested concentrations; however, some compounds showed NRF2 activation only at high concentrations and failed to show a gradual activation in NRF2-induced luciferase signal with increasing concentration (Figures S1 and S2, Table 1).

Hit compounds were selected by comparing the EC_50_ values of each compound, prioritizing those with lower EC_50_ values and no cytotoxic effects at 50 µM. The compound OIC18 showed a low EC_50_ value (3.6 µM); however, it exhibited strong cytotoxicity. OIC6 showed a comparable EC_50_ (6.8 µM) but a substantially lower maximal ARE–luciferase induction (14-fold, versus 71- and 39-fold for OIC3 and OIC15) and was therefore not selected. In addition, some compounds showed maximum NRF2 induction only at high concentrations, whereas the induction fold decreased drastically at lower concentrations, such as OIC2, OIC5, and OIC9; therefore, their EC_50_ values dropped significantly (Figures S1 and S2, Table 1). The compounds OIC3 and OIC15 showed low EC_50_ values, did not exhibit any cytotoxicity in ARE-MCF7 cells, and showed maximal NRF2 induction; therefore, these two compounds were selected for further analysis, whereas OIC18, despite its low EC_50_ value, was excluded due to its cytotoxicity. Although the overall dose–response relationship was not strongly pronounced, OIC3 and OIC15 showed a tendency toward increased NRF2 activation with increasing concentrations.

In terms of the structure–activity relationship (SAR), derivatives bearing electron-withdrawing groups (EWGs), such as chloro and bromo substituents, showed greater potency than those bearing electron-donating groups (EDGs), such as methoxy and hydroxy groups. Among the chloro- and bromo-substituted derivatives, the *ortho*-substituted compounds exhibited greater potency than their corresponding *meta*- and *para*-substituted analogues. Because this positional preference could reflect either an electronic or a steric effect on the Michael acceptor, the electronic structures of all 19 derivatives were examined computationally; the results are described in Section 2.6.

### 2.3. OIC3 and OIC15 Dose-Dependently Induce the NRF2-ARE Signaling Pathway

OIC3 and OIC15 were chosen as hit compounds to study the NRF2 signaling pathway in detail. The compounds were applied to ARE-MCF7 cells at different concentrations to examine NRF2 protein levels and NRF2-ARE target genes by immunoblotting. Both compounds were treated for 24 h, after which samples were collected and immunoblots were performed.

**Figure 2:**
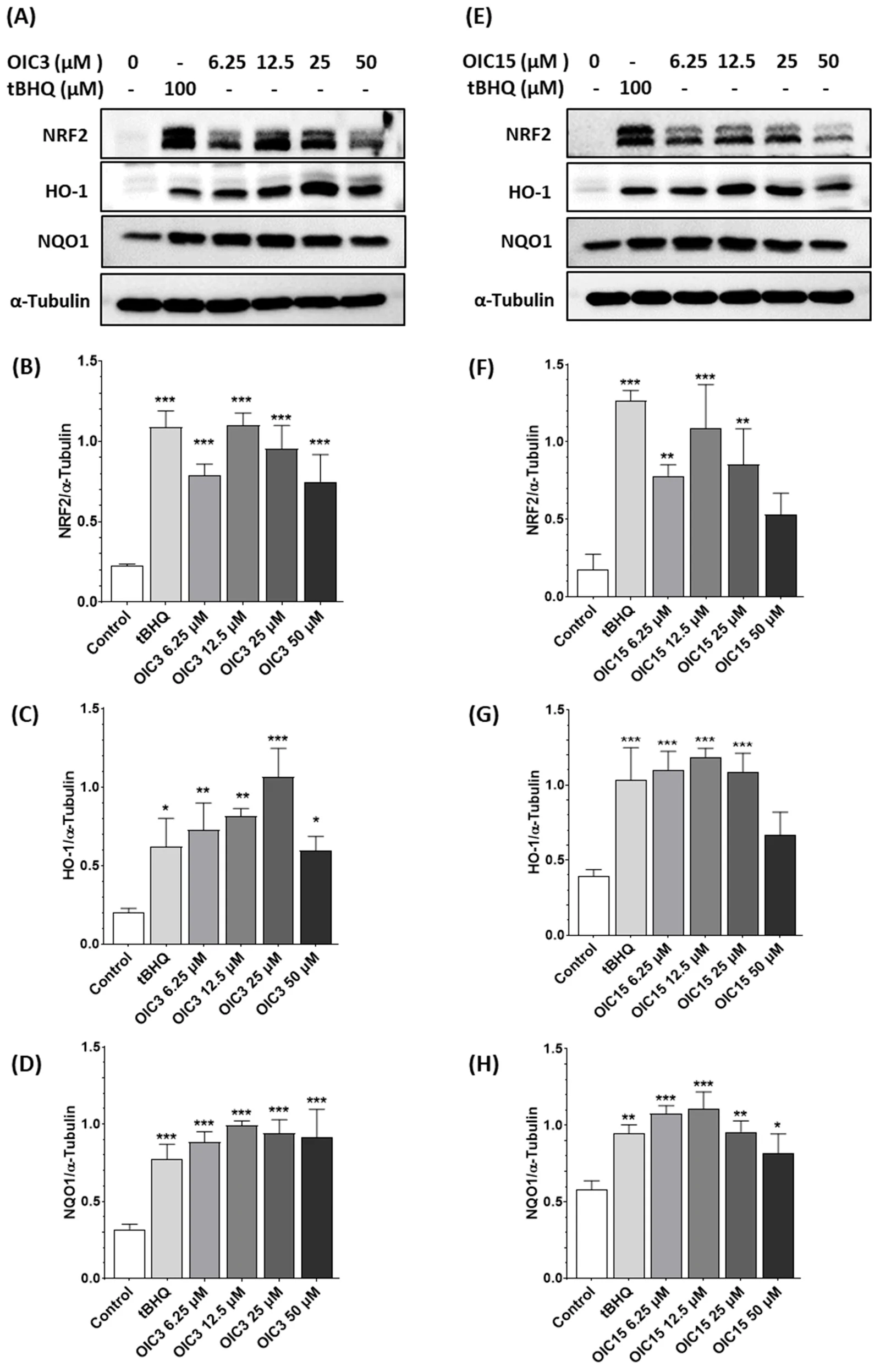
Effect of OIC3 and OIC15 on the expression of NRF2 and its downstream antioxidant proteins in ARE-MCF7 cells. (**A**) Representative western blots showing the expression of NRF2, HO-1, and NQO1 after treatment of OIC3. Densitometric analysis of the western blot of NRF2 (**B**), HO-1 (**C**), and NQO1 (**D**). (**E**) Representative western blots showing the expression of NRF2, HO-1, and NQO1 after treatment of OIC15. Densitometric analysis of the western blot of NRF2 (**F**), HO-1 (**G**), and NQO1 (**H**). The compounds were treated at indicated concentrations, and tBHQ for 24 h in ARE-MCF7 cells and immunoblot was performed by loading equal amounts of proteins in each wells. The bar graphs were generated with three independent experiments. Statistical significance, *** *p* < 0.001, ** *p* < 0.01, * *p* < 0.05.

The data show that NRF2 activation induced by both compounds increased at the tested concentrations from 6.3 to 25 µM and decreased at 50 µM at the 24 h time point, but remained higher than the DMSO control (Figure 2). tBHQ, a known activator of NRF2-ARE signaling, was used as a positive control, and both OIC3 and OIC15 induced NRF2 signaling at levels comparable to tBHQ.

NRF2 target genes HO-1 and NQO1 were then examined to determine whether NRF2 activation led to the induction of downstream target genes involved in the oxidative stress response. Both compounds significantly induced HO-1 and NQO1 protein levels after treatment and showed a dose-dependent increase in protein expression (Figure 2). Overall, the data show that both compounds induced NRF2 and its target genes HO-1 and NQO1 in a concentration-dependent manner at the 24 h time point.

### 2.4. Time-Course Analysis of NRF2-ARE Signaling Activation by OIC3 and OIC15

To examine the time-dependent activation of NRF2 by OIC3 and OIC15, the EC_50_ concentrations were selected for analysis. The EC_50_ values were 5 µM for OIC15 and 6.2 µM for OIC3. ARE-MCF7 cells were treated with these concentrations at different time points (4, 8, 12, 24, and 48 h), and immunoblotting was performed to assess NRF2 and target gene protein levels.

**Figure 3:**
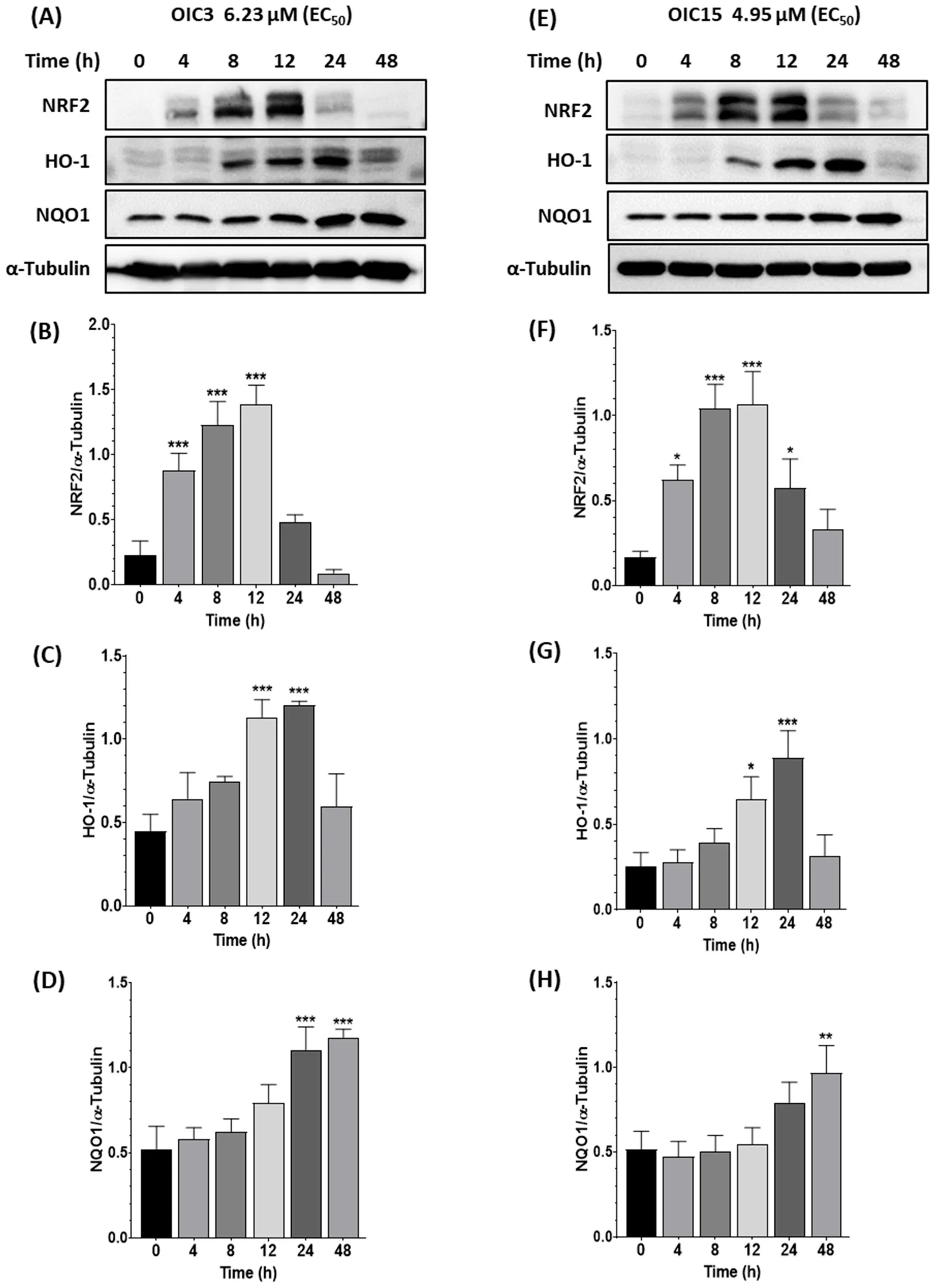
Effect of OIC3 and OIC15 on the expression of NRF2 and its downstream antioxidant proteins in ARE-MCF7 cells at different time points. (**A**) Representative western blots showing the expression of NRF2, HO-1, and NQO1 after treatment of OIC3. Densitometric analysis of the western blot of NRF2 (**B**), HO-1 (**C**), and NQO1 (**D**). (**E**) Representative western blots showing the expression of NRF2, HO-1, and NQO1 after treatment of OIC15. Densitometric analysis of the western blot of NRF2 (**F**), HO-1 (**G**), and NQO1 (**H**). The EC_50_ concentrations of OIC3 (6.2 µM) and OIC15 (5 µM) were treated at indicated concentrations for 24 h in ARE-LUC in MCF7 cells and immunoblot was performed by loading equal amounts of proteins in each wells. The bar graphs were generated with three independent experiments. Statistical significance, *** *p* < 0.001, ** *p* < 0.01, * *p* < 0.04.

The data show that both OIC3 and OIC15 induced NRF2 protein levels as early as 4 h, with a gradual increase reaching a maximum at 12 h, followed by a decrease toward 48 h (Figure 3). In contrast, HO-1 protein levels increased from 8 h, reached a maximum at 24 h, and decreased at 48 h, with both compounds showing a similar pattern (Figure 3). However, NQO1 protein levels increased continuously from 8 h up to 48 h in response to both compounds.

Overall, these data clearly demonstrate that at their EC_50_ concentrations, both compounds induced NRF2 protein expression from 4 h and significantly induced target gene protein expression from 8 h, with saturation occurring at later time points, indicating robust activation of the NRF2-ARE signaling pathway and its downstream target genes.

### 2.5. Anti-Inflammatory Effects of OIC3 and OIC15 in RAW264.7 Cells

**Figure 4:**
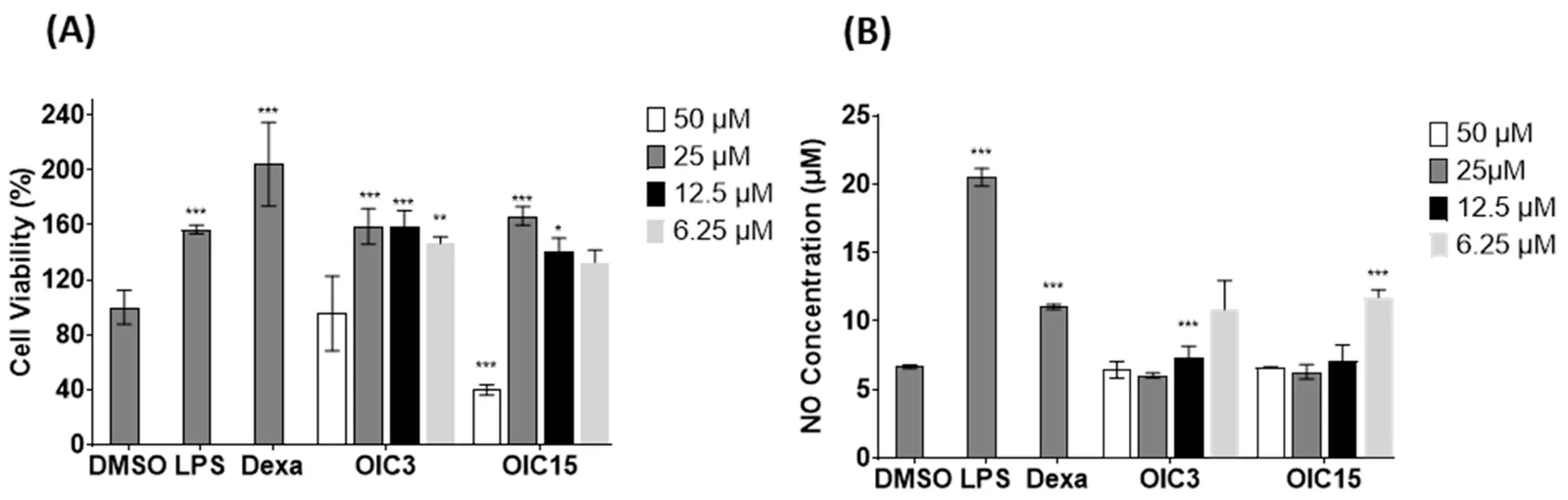
anti-inflammatory effects of OIC3 and OIC15 in Raw264.7 cells. (**A**) The cell viability of OIC3 and OIC15. (**B**) NO product of OIC3 and OIC15. Statistical significance, *** *p* < 0.001, ** *p* < 0.007, * *p* < 0.02.

It is known that activation of NRF2 can have antioxidant and anti-inflammatory effects [40]. We therefore tested OIC3 and OIC15 on NO product by LPS induced RAW264.7 cells. Before evaluating the anti-inflammatory potential of the compounds OIC3 and OIC15, their cytotoxic effects were first assessed in murine RAW 264.7 macrophages to exclude nonspecific toxicity that could affect any observed biological activities. Cells were pretreated with each compound at concentrations of 50, 25, 12.5, and 6.3 µM for 2 h, followed by stimulation with LPS (1 µg/mL) for an additional 22 h. In Figure 4A, OIC15 exhibited reduced cell viability, lower than around 40% at 50 µM, whereas OIC3 maintained a higher safety in the range of testing concentrations. The anti-inflammatory activities of OIC3 and OIC15 were subsequently evaluated by measuring nitric oxide (NO) production in LPS-stimulated RAW 264.7 cells. Treatment with LPS markedly induced NO release, while dexamethasone significantly suppressed NO levels, serving as a positive control. Notably, both OIC3 and OIC15 effectively inhibited NO production in a concentration-dependent manner, as shown in Figure 4B. Although OIC15 showed slightly stronger NO inhibitory activity, it also induced toxicity at the highest concentration. In contrast, OIC3 demonstrated significant suppression of NO production while maintaining cell viability.

### 2.6. In Silico Evaluation of the Michael Acceptor Properties of the Oxindole Scaffold

**Figure 5:**
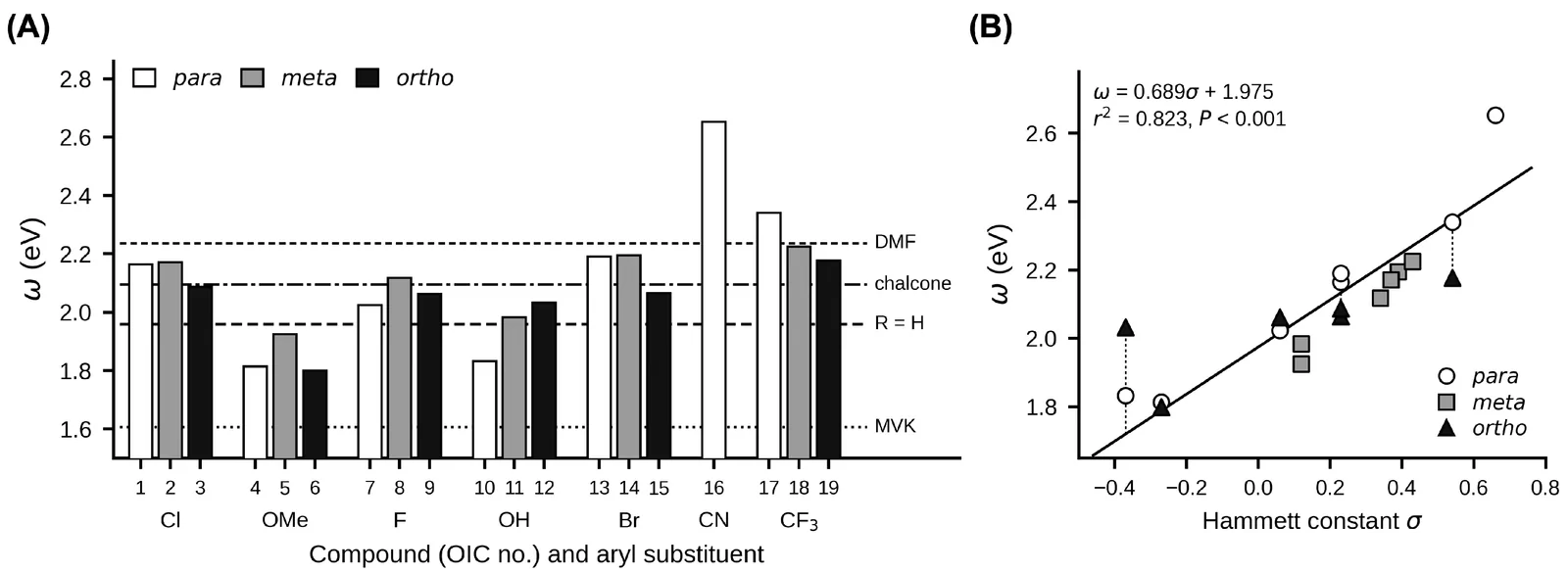
In silico characterization of the oxindole scaffold as a Michael acceptor. (**A**) Global electrophilicity index ω; bars are labeled with the compound number and grouped by aryl substituent, and fill indicates the substitution position. Horizontal lines mark the values for methyl vinyl ketone (MVK), the unsubstituted scaffold (R = H), *trans*-chalcone and dimethyl fumarate (DMF). (**B**) Correlation of ω with the Hammett constant σ. The regression line was fitted to the *para* and *meta* derivatives only; the *ortho* derivatives are plotted at σp for reference, with dotted lines indicating their deviation from the fitted line.

To assess whether the oxindole scaffold possesses the electronic features expected of a Michael acceptor, density functional theory calculations were carried out on all 19 derivatives and on four reference Michael acceptors treated identically (Figure 5, Table S1). The condensed Fukui function *f*^+^ [41] identified the exocyclic benzylidene carbon (C_β_) as the site most susceptible to nucleophilic attack in every compound, with the oxindole C3 ranked second and carrying about half the *f*^+^ value. The calculated global electrophilicity index ω [42] ranged from 1.80 eV (OIC6) to 2.65 eV (OIC16), placing all 19 derivatives in the strong-electrophile region of the scale defined at the same level of theory (ω > 1.5 eV) [43,44]. For comparison, methyl vinyl ketone, a standard model substrate for thiol conjugate addition [45], gave 1.61 eV, the unsubstituted parent scaffold gave 1.96 eV, *trans*-chalcone gave 2.09 eV, and dimethyl fumarate gave 2.24 eV (Figure 5A). The series therefore spans the electrophilicity range of chalcone-type NRF2 activators [18] and dimethyl fumarate [46].

Across the 13 *para*- and *meta*-substituted derivatives, ω correlated linearly with the Hammett substituent constant σ [47] (ω = 0.689σ + 1.975; *r*^2^ = 0.823, *n* = 13, *p* < 0.001; Figure 5B). As σ is an independent experimental scale, this agreement provides an internal check on the protocol. Because σ is defined only for *meta* and *para* substituents [47], the six *ortho* derivatives were excluded from the regression. The lower ω of the ortho isomers has a steric origin: the optimized aryl–vinyl dihedral angle was larger for the *ortho* isomer than for the corresponding *para* isomer in all six substituent series (mean difference 7.1 ± 4.3°; paired *t*-test, *p* = 0.010; Table S1). Within the chloro and bromo series, the *ortho* isomer had the lowest ω of the three (OIC3, 2.09 eV; OIC15, 2.07 eV) while being the most potent. The positional effect described in Section 2.2 therefore cannot be attributed to a difference in intrinsic thiol reactivity; within this congeneric series electrophilicity is a necessary rather than a discriminating property. These descriptors, however, were calculated for isolated molecules in the gas phase and do not account for solvation.

**Figure 6:**
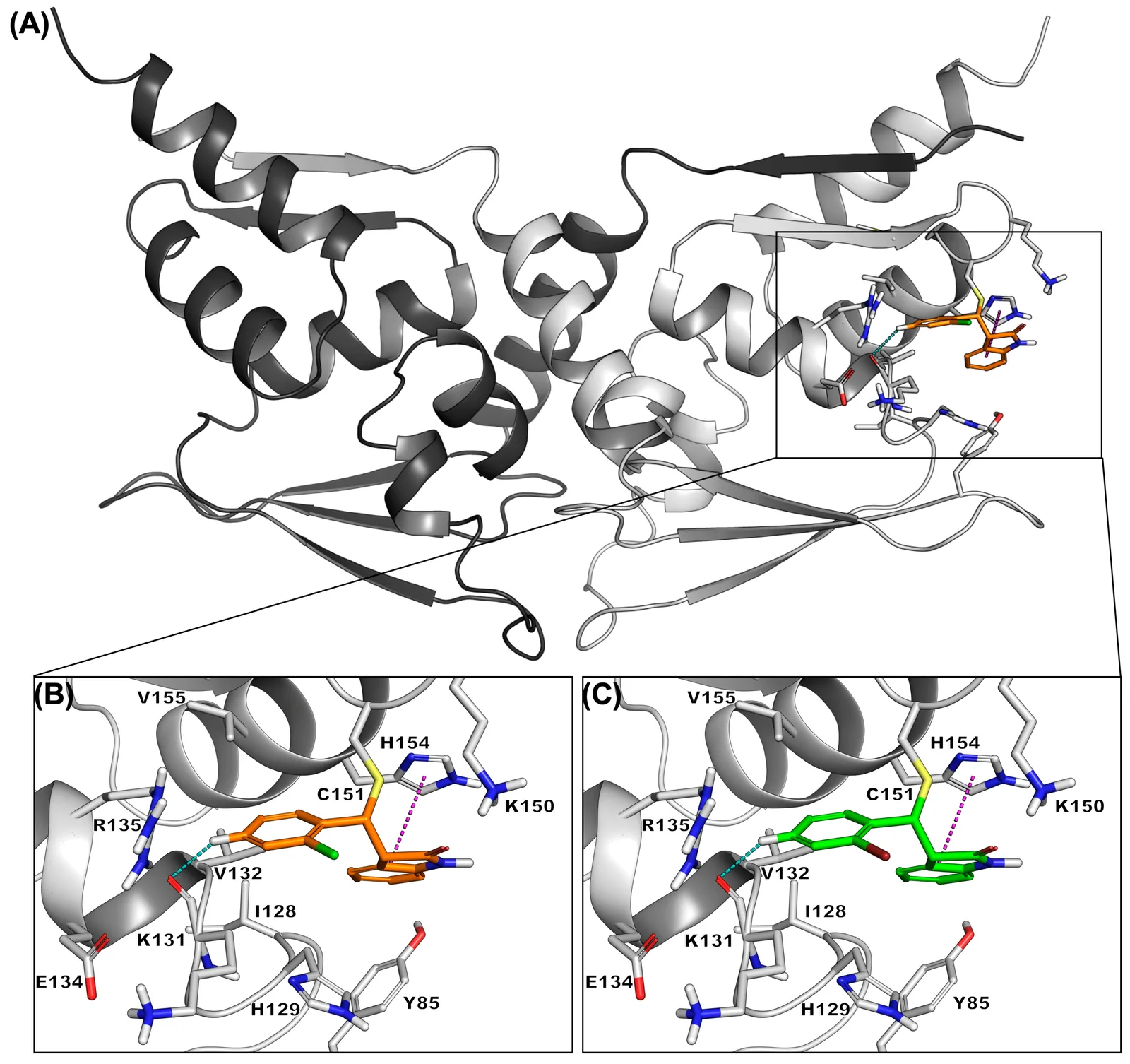
Covalent docking of OIC3 and OIC15 to Cys151 of the KEAP1 BTB domain (PDB 4CXT). (**A**) The BTB homodimer, with the ligand-bound protomer in light grey and the second protomer in dark grey; OIC3 is shown as orange sticks and the boxed region is enlarged below. (**B**) The OIC3 adduct, ligand in orange; (**C**) the OIC15 adduct, ligand in green. Residues within 5 Å of the ligand are shown as sticks and labeled in one-letter code. Magenta dashed lines, π–π stacking with His154; cyan dashed lines, aromatic C–H···O contact with the Lys131 backbone carbonyl.

To examine whether the corresponding adducts can be accommodated in the site, OIC3 and OIC15 were docked covalently to Cys151 using the KEAP1 BTB domain in complex with CDDO (PDB 4CXT) as the receptor [48]. Redocking of the co-crystallized ligand reproduced the experimental binding mode with a heavy-atom RMSD of 0.53 Å, compared with a mean of 1.52 Å reported for the method [49]. Both compounds were accommodated without steric conflict and adopted essentially the same binding mode, the two poses differing by 0.006 Å over the common scaffold (Table S2) and contacting the same residues; the oxindole packed against His129, Lys150 and His154, and the aryl ring against Lys131, Val132, Arg135 and Val155 (Figure 6). In both poses the oxindole benzo ring formed a parallel-displaced π–π stacking interaction with the imidazole of His154, and the aryl ring a weak aromatic C–H···O contact with the backbone carbonyl oxygen of Lys131. The *ortho* halogen was directed toward solvent and made no close contact with the protein, consistent with a steric rather than a direct interaction role. Docking scores were used only to select a representative pose, not to rank the derivatives (Table S2). The calculations all assumed the E isomer, although 3-arylideneoxindoles are known to isomerize in solution under acidic, basic or photochemical conditions [50], and the isomeric composition under the assay conditions was not determined. Taken together, the calculations support the Michael acceptor mechanism proposed in Section 2.1 but indicate that the differences in cellular potency within the series arise from factors other than intrinsic electrophilicity.

## 3. Materials and Methods

### 3.1. Chemicals and Reagents

All chemicals were purchased from Sigma-Aldrich (St. Louis, MO, USA) or Alfa Aesar (Haverhill, MA, USA). The ONE-Glo assay kit was obtained from Promega (Madison, WI, USA), and DMEM high-glucose with penicillin/streptomycin was purchased from HyClone (Logan, UT, USA). The ARE-LUC MCF7 cells were purchased from Signosis (Santa Clara, CA, USA). The antibodies, NRF2 (12721), α-Tubulin (2144), NQO1 (62262), and HO-1 (43966), were purchased from Cell Signaling Technology (Danvers, MA, USA).

### 3.2. Synthesis of the Oxindole Derivatives

Indolin-2-one (1.0 equiv.) and the corresponding carboxaldehyde (1.0–1.4 equiv.) were dissolved in methanol (5 mL), followed by the addition of piperidine (1.0–2.0 equiv.) or 50% aqueous NaOH. The reaction mixture was heated under reflux until the starting material was completely consumed, as monitored by thin-layer chromatography (TLC). After completion, the solvent was removed under reduced pressure, and the residue was partitioned between ethyl acetate and water. The organic layer was collected and dried, and the solvent was evaporated under reduced pressure. The resulting residue was purified by silica gel column chromatography or preparative HPLC using a linear gradient of 10% acetonitrile (ACN)/90% H_2_O to 100% ACN over 60 min to afford the desired compounds. All synthesized compounds were characterized by ^1^H NMR and ^13^C NMR spectroscopy and LC–MS analysis.

### 3.3. Quantum Chemical Calculations and Covalent Docking

Quantum chemical calculations were performed with Jaguar (Schrödinger Release 2026-3; Schrödinger LLC, New York, NY, USA) [51]. Structures were generated from SMILES and prepared with LigPrep at pH 7.4, with the *E* configuration about the exocyclic C3=CH bond specified explicitly, and a conformational search was carried out for each compound with MacroModel using the OPLS4 force field [52], giving 106 conformers across the 23 structures. Rather than pre-selecting a single force-field minimum, every conformer was optimized independently using the B3LYP functional [46,53] with the 6-31G* basis set [54] in the gas phase. This level was chosen because the electrophilicity scale used for classification is defined at B3LYP/6-31G(d) [43,44], and no solvation model was applied for the same reason. Conformers agreeing in electronic energy to within 0.05 kcal mol^−1^ were treated as the same minimum and the lowest-energy unique conformer of each compound was used for analysis. As a check, Boltzmann-weighted values at 298 K were also computed, and every optimization was continued from its converged geometry until all convergence criteria were satisfied (Table S1). Reactivity descriptors were obtained from the frontier orbital energies as μ = (*E*_HOMO_ + *E*_LUMO_)/2, η = *E*_LUMO_ − *E*_HOMO_ and ω = μ^2^/(2η) [42,55], with 1 hartree = 27.2114 eV, and the condensed Fukui function f^+^ for nucleophilic attack, derived from the LUMO [41]. Methyl vinyl ketone, (*E*)-3-benzylidene-2-oxindole, *trans*-chalcone and dimethyl fumarate were calculated identically as reference electrophiles. The aryl–vinyl dihedral angle was taken as the mean of the C_α_=C_β_−C_ipso_−C_ortho_ torsions measured through the two *ortho* carbons, folded into the 0–90° range.

For covalent docking, the KEAP1 BTB domain in covalent complex with CDDO (PDB 4CXT, 2.66 Å) [48] was used as the receptor and the biological assembly was prepared with the Protein Preparation Wizard [56] at pH 7.4. Docking was performed with CovDock [49] in pose prediction mode, using the Michael addition reaction type and Cys151 as the reactive residue, and the protocol was validated by redocking the co-crystallized ligand (Table S2). RMSD was calculated in place, without superposition, using an atom correspondence obtained from graph isomorphism of the heavy-atom skeletons. For each ligand the pose with the best cdock affinity was selected; since CDDO contains two enone systems, only poses in which the covalent bond was formed at the A-ring carbon modified in the crystal structure were considered. Contact residues were defined as those with any atom within 5 Å of the ligand, and π–π stacking geometry by the separation between ring centroids and the angle between the ring planes.

### 3.4. Cell Culture Condition

ARE-MCF7 cells were obtained from Signosis and grown in high-glucose DMEM (Cytiva, Marlborough, MA, USA) supplemented with 10% FBS (Gibco-Thermo Fisher Scientific, Waltham, MA, USA) and 1% penicillin/streptomycin (Gibco-Thermo Fisher Scientific, Waltham, MA, USA), under 5% CO_2_ at 37 °C.

### 3.5. NRF2-ARE Activation Screening by ONE-Glo Assay

ARE-MCF7 cells (20,000 cells per well) were seeded in 96-well plates (Greiner Bio-One, Kremsmünster, Austria) and incubated for 24 h at 37 °C under 5% CO_2_. The cells were then treated with the compounds at the indicated concentrations and incubated for an additional 24 h. Subsequently, 50 µL of fivefold-diluted ONE-Glo reagent was added to each well and mixed thoroughly. Luminescence was measured using a GloMax multi-plate reader (Promega, Madison, WI, USA).

### 3.6. Cell Viability Assay

ARE-MCF7 cells (20,000 cells per well) were seeded in 96-well plates (Corning, Corning, NY, USA) and cultured for 24 h at 37 °C under 5% CO_2_. The compounds were then added to each well, and after 24 h of incubation, the medium was removed. Fresh growth medium containing 10% Ez-Cytox reagent was added, and the cells were incubated for 30 min. Absorbance was measured at 450 nm using a Tecan multi-plate reader (Männedorf, Switzerland).

### 3.7. Western Blot

ARE-MCF cells (5 × 10^5^ cells per well) were seeded in 6-well plates (Corning, USA) and cultured for 24 h at 37 °C under 5% CO_2_. The cells were then treated with the indicated compounds and harvested. Cell lysates were prepared using cell extraction buffer (Invitrogen, Carlsbad, CA, USA) supplemented with a protease inhibitor cocktail (Roche, Indianapolis, IN, USA) by incubating on ice for 30 min with intermittent vortexing. The lysates were centrifuged at 14,000 rpm for 20 min, and the supernatants were collected. Total protein concentration was determined using a BCA assay kit (Thermo Fisher Scientific, Waltham, MA, USA). Equal amounts of protein (20 µg) were loaded into each well of SDS-PAGE gels and resolved at 100 V for 90 min, followed by transfer to PVDF membranes at 100 V for 90 min. The membranes were incubated with the respective primary antibodies overnight at 4 °C and with secondary antibodies for 90 min at room temperature. Immunoblots were developed using chemiluminescent detection reagents.

### 3.8. Statistical Analysis

All experiments were performed in triplicate, and graphs were generated using GraphPad Prism software (Version 11.1.0). Statistical significance was calculated using one-way ANOVA followed by Tukey’s post hoc test. Correlations between calculated descriptors and Hammett substituent constants were evaluated by Pearson regression, and paired comparisons of dihedral angles by two-tailed paired *t*-test.

## 4. Conclusions

In this study, we aimed to identify novel and potent oxindole-based NRF2 activators with minimal cytotoxicity. Nineteen oxindole derivatives were obtained in acceptable yields and evaluated in ARE-MCF7 cells for NRF2 activation and cytotoxicity. All tested compounds exhibited NRF2 activation, and OIC3 and OIC15 were identified as the most promising candidates based on their low EC_50_ values, minimal cytotoxicity, and strong NRF2 activation. Further mechanistic evaluation demonstrated that both compounds increased NRF2 protein levels in a dose-dependent manner and activated the NRF2–ARE pathway at the transcriptional level. In particular, OIC3 and OIC15 significantly increased the expression of the downstream antioxidant proteins HO-1 and NQO1, with their induction becoming evident from 8 h and NQO1 remaining elevated for up to 48 h. Quantum chemical calculations placed all 19 derivatives within the strong-electrophile range and identified the exocyclic benzylidene carbon as the reactive center, and covalent docking indicated that the corresponding Cys151 adducts can be accommodated in the KEAP1 BTB domain without steric conflict. These results are consistent with the proposed mechanism, although they do not by themselves demonstrate direct binding to KEAP1.

Collectively, these findings identify OIC3 and OIC15 as potent oxindole-based NRF2 activator leads and demonstrate that this chemical scaffold can be used to develop small molecule modulators of the NRF2–ARE antioxidant pathway. This study provides a foundation for further structure–activity relationship optimization and mechanistic investigations of oxindole derivatives. Future studies, including target engagement, selectivity, pharmacokinetic evaluation, and in vivo efficacy and safety studies, will be necessary to determine their therapeutic applicability.

## Figures and Tables

**Table 1:** Oxindole compounds, ARE-LUC activation and cell viability. ND, not determined.

S.No	OIC	Aldehyde	Yield (%)	EC_50_ (µM)	Max ARE-LUC (Fold)	Viability at 50 µM (% Live)
1	OIC1	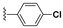	27.7	58.2	33	116%
2	OIC2	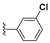	30.5	11.6	73	89%
3	OIC3	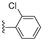	30.7	6.2	71	105%
4	OIC4	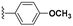	26.7	ND	6.8	101%
5	OIC5	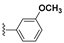	22.7	41	46	119%
6	OIC6	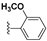	29	6.8	14	126%
7	OIC7	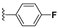	30.1	ND	18	125%
8	OIC8	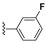	36.7	ND	28	110%
9	OIC9	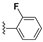	26.7	20	50	133%
10	OIC10	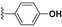	33.3	ND	4.3	105%
11	OIC11	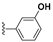	33.3	37.4	6.3	88%
12	OIC12	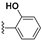	36.7	ND	6.6	90%
13	OIC13	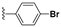	28.7	27.3	3.8	102%
14	OIC14	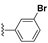	23.7	9.3	42	94%
15	OIC15	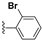	31.3	5	39	115%
16	OIC16	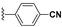	31.7	ND	6.2	91%
17	OIC17	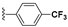	27.7	26	8.2	107%
18	OIC18	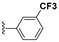	31	3.6	53	55%
19	OIC19	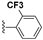	29	16.7	2	96%

## Data Availability

The original contributions presented in the study are included in the further inquiries can be directed to the corresponding authors.

## Supplementary Materials

The following supporting information can be downloaded at: https://www.mdpi.com/article/10.3390/molecules31183314/s1, Figure S1. Oxindole compounds (1–19) EC_50_ graphs. The compounds were treated on ARE-MCF7 cells for 24 h and ONE-Glo assay was performed and luminescence data was generated. Then, EC_50_ graphs were generated with GraphPad Prism software. (N = 3). Figure S2. Oxindole compounds (1–19) cytotoxicity data. The compounds, tBHQ 50 µM were treated on ARE-MCF7 cells for 24 h, Ez-Cytox reagent was added and OD was measured at 450 nm. Then, the graphs were generated with GraphPad Prism software. (N =3 ). Table S1. Quantum chemical descriptors for the oxindole derivatives and reference Michael acceptors. Table S2. Covalent docking of OIC3 and OIC15 to Cys151 of the KEAP1 BTB domain (PDB 4CXT).
