# Supplementary material for: Oxindole Compounds Induce NRF2-ARE Antioxidant Pathway in MCF7 Cells and Reduce Inflammatory Response in RAW264.7 Cells
Source: Molecules. 2026 Sep 18;31(18):3314. doi: 10.3390/molecules31183314 (PMC13609623; doi:10.3390/molecules31183314)
Supplement: Supplementary file 1 [file molecules-31-03314-s001.zip › molecules-4472264-supplementary.pdf]

Supplementary Information

Oxindole compounds induce NRF2-ARE antioxidant pathway in MCF7 cells and reduce inflammatory response in RAW264.7 cells

Baskar Salvaraj[1], Qui Ngoc Sang Nugyen[1,3,5], Seong-Hee Ko[2], Ki-Yeon Yoo[3], Suk Woo Kang[1*], Jae Wook Lee[1,4,*]

[1]Center for Natural Product Efficacy Optimization, Korea Institute of Science and Technology (KIST), Gangneung Institute, Gangneung, 25451, Republic of Korea

[2]Department of Pharmacology, College of Dentistry, Gangwon National University, Gangneung, 25457, Republic of Korea

[3]Department of Anatomy, College of Dentistry, Gangwon National University, Gangneung, 25457, Republic of Korea

[4]Natural Product Applied Science, KIST School, University of Science and Technology, Gangneung, 25451, Republic of Korea

[5]Insitute of Natural Product Chemistry, Vietnam Academy of Science and Technology (VAST), Hanoi, 100000, Vietnam

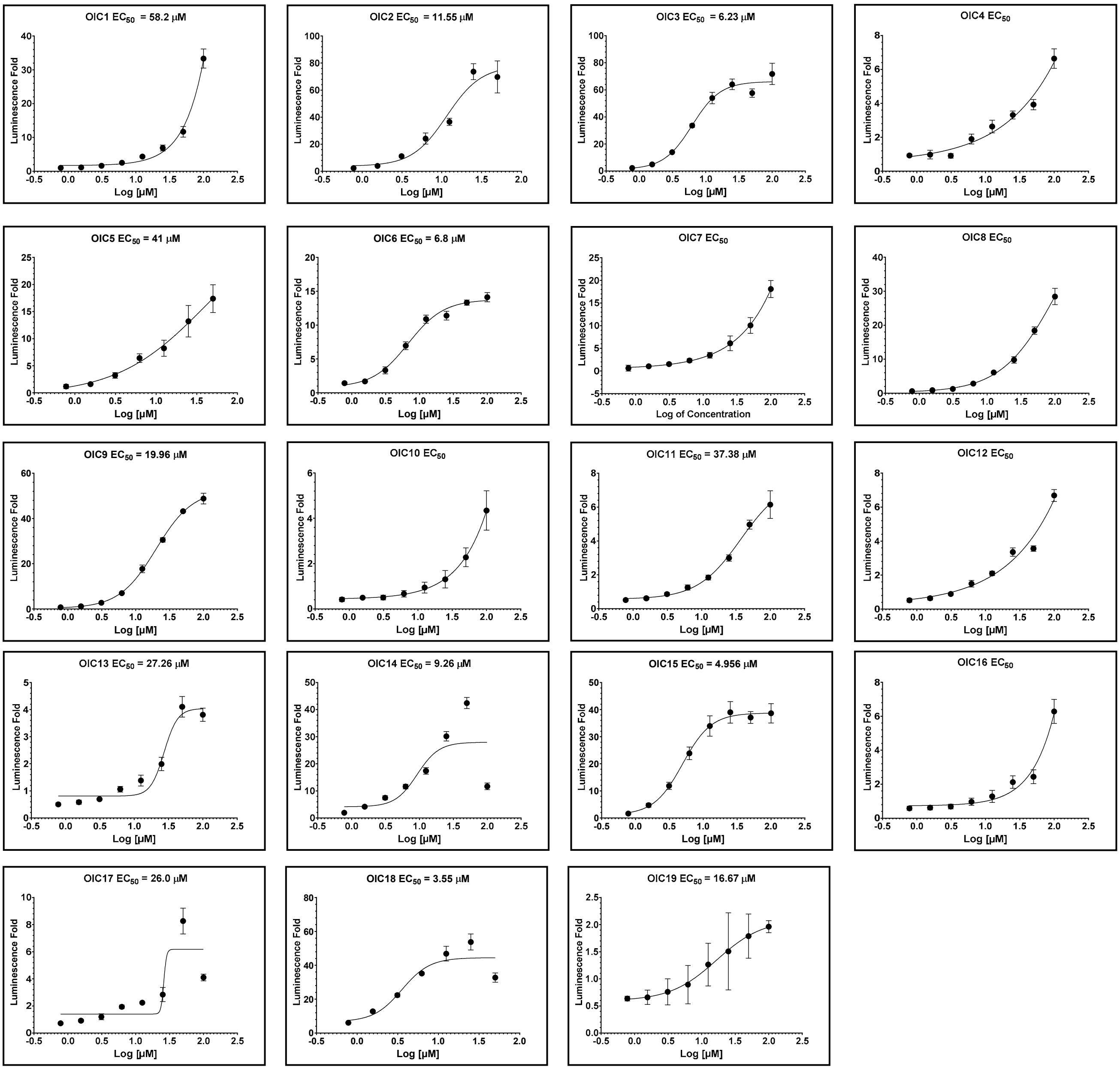

Figure S1. Oxindole compounds (1-19) EC_{50} graphs. The compounds were treated on ARE-MCF7 cells for 24 h and ONE-Glo assay was performed and luminescence data was generated. Then, EC_{50} graphs were generated with GraphPad Prism software. (N=3).

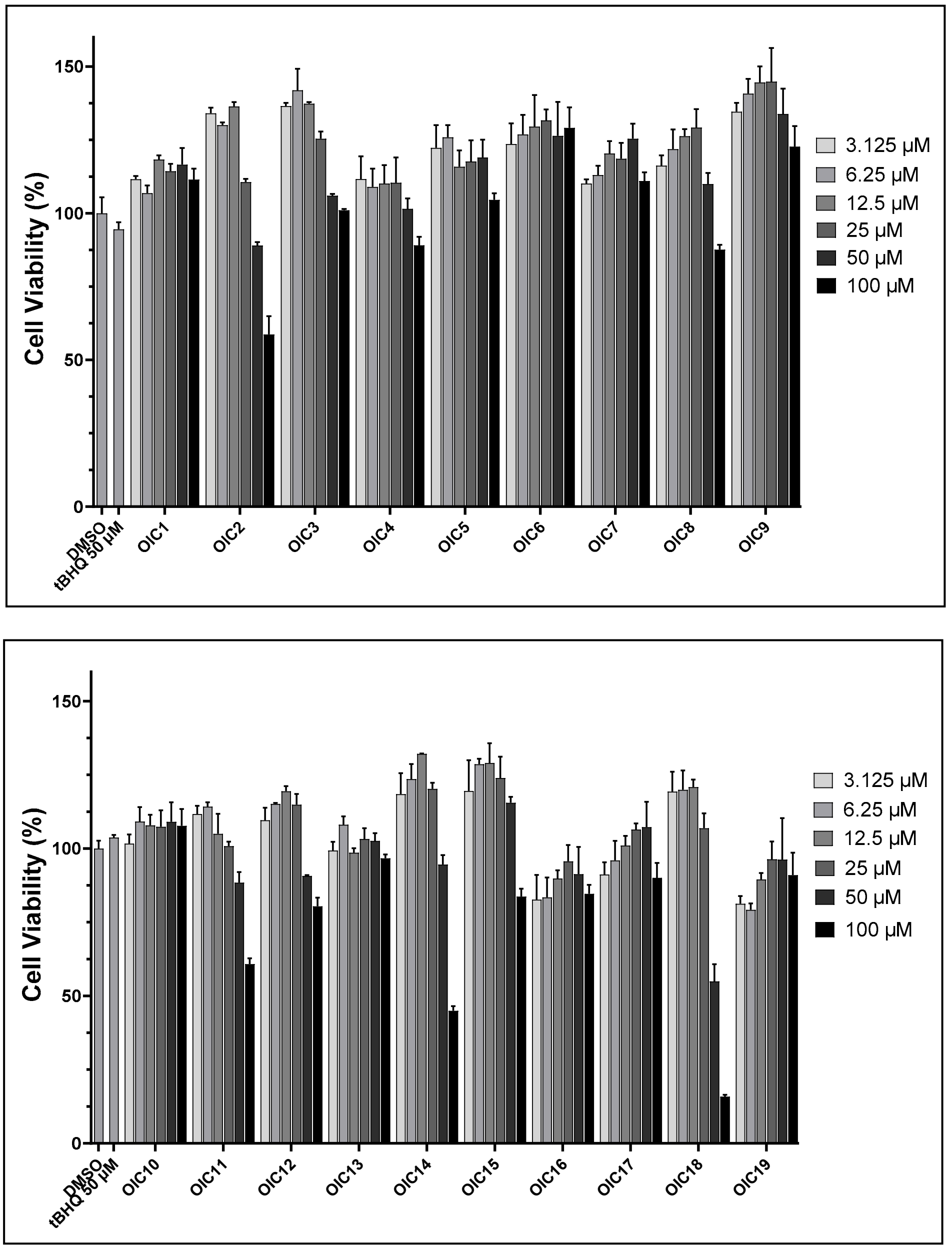

Figure S2. Oxindole compounds (1-19) cytotoxicity data. The compounds, tBHQ 50 μM were treated on ARE-MCF7 cells for 24 h, Ez-Cytox reagent was added and OD was measured at 450 nm. Then, the graphs were generated with GraphPad Prism software. (N=3).

Experimental Section

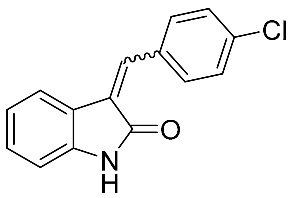

3-(4-chlorobenzylidene)indolin-2-one(OIC1)

Indolin-2-one (39.0 mg, 0.3 mmol) was dissolved in methanol (1 mL). 4-chlorobenzaldehyde (42.2 mg, 0.3 mmol) and piperidine (30.0 μL, 0.36 mmol) was added to reaction mixture sequentially. The reaction mixture was refluxed and stirred for 8 h. The yellow solid (21.2 mg, 27.7%) was obtained by column chromatography.

^{1}H NMR (400 MHz, $CDCl_3$) δ 8.40 (s, 1H), 7.75 (s, 1H), 7.61-7.56 (m, 3H), 7.45 (d, *J* = 8.5 Hz, 2H), 7.23 (t, *J* = 7.7 Hz, 1H), 6.91-6.86 (m, 2H). ^{13}C NMR (100 MHz, $CDCl_3$) δ 170.0, 141.8, 136.0, 135.7, 133.4, 130.8, 130.3, 129.2, 128.1, 123.2, 122.1, 121.6, 110.4. MS (m/z): $[M + H]^+$ calcd for $C_{15}H_{10}ClNO$, 256.05; found 256.1.

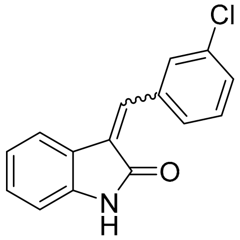

3-(3-chlorobenzylidene)indolin-2-one(OIC2)

Indolin-2-one (39.0 mg, 0.3 mmol) was dissolved in methanol (1 mL). 3-chlorobenzaldehyde (42.2 mg, 0.3 mmol) and piperidine (30.0 μL, 0.36 mmol) was added to reaction mixture sequentially. The reaction mixture was refluxed at 80 °C and stirred for 8 h. After completion of the reaction, the reaction mixture was cooled to room temperature (15-25 °C), and then the reaction mixture was concentrated under reduced pressure. The residue was dissolved in EA (50 mL), and H_2O (30 mL). The solution was transferred to separatory funnel, organic layers were separated. The organic layer was dried over Na_2SO_4, and filtered. The solvent was removed under reduced pressure and residue was purified by prep-LC (ACN / H_2O, 10% ACN to 100% ACN for 1 h, linear gradient) to give the yellow solid (23.5 mg, yield = 30.7%). ^{1}H NMR (400 MHz, $CDCl_3$) δ 8.89 (s, 1H), 7.74 (s, 1H), 7.63 (s, 1H), 7.55-7.53 (m, 2H), 7.40 (d, *J* = 5.35 Hz, 2H), 7.23 (t, *J* = 7.6 Hz, 1H), 6.93 (m, 1H), 6.89 (m, 2H). ^{13}C NMR (100 MHz, $CDCl_3$) δ 169.9, 141.9, 136.8, 135.6, 134.9, 130.5, 130.2, 129.7, 129.2, 128.8, 127.4, 123.3, 122.2, 121.5, 110.5. MS (m/z): $[M + H]^+$ calcd for $C_{15}H_{10}ClNO$, 256.05; found 256.1.

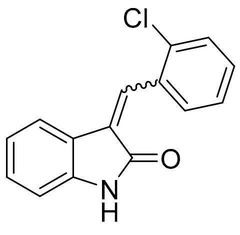

3-(2-chlorobenzylidene)indolin-2-one(OIC3)

Indolin-2-one (39.0 mg, 0.3 mmol) was dissolved in methanol (1 mL). 2-chlorobenzaldehyde (42.2 mg, 0.3 mmol) and piperidine (30.0 μL, 0.36 mmol) was added to reaction mixture sequentially. The reaction mixture was refluxed at 80 °C and stirred for 8 h. After completion of the reaction, the reaction mixture was cooled to room

temperature (15-25 °C), and then the reaction mixture was concentrated under reduced pressure. The residue was dissolved in EA (50 mL), and H_2O (30 mL). The solution was transferred to separatory funnel, organic layers were separated. The organic layer was dried over Na_2SO_4, and filtered. The solvent was removed under reduced pressure and residue was purified by prep-LC (ACN / H_2O, 10% ACN to 100% ACN for 1 h, linear gradient) to give the yellow solid (23.4 mg, yield = 30.7%). ^{1}H NMR (400 MHz, $CDCl_3$) δ 8.72 (s, 1H), 7.88 (s, 1H), 7.73 (dd, *J* = 7.5, 1.8 Hz, 1H), 7.52-7.33 (m, 3H), 7.22 (td, *J* = 7.7, 1.2 Hz, 1H), 6.92 (d, *J* = 7.75 Hz, 1H), 6.83 (td, *J* = 7.75, 1 Hz, 1H). ^{13}C NMR (100 MHz, $CDCl_3$) δ 169.9, 142.0, 134.6, 134.0, 133.7, 130.9, 130.4, 130.3, 130.2, 129.2, 126.8. 123.4, 122.1, 121.5, 110.5. MS (m/z): $[M + H]^+$ calcd for $C_{15}H_{10}ClNO$, 256.05; found 256.1.

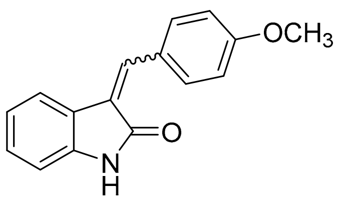

3-(4-methoxybenzylidene)indolin-2-one(OIC4)

Indolin-2-one (39.0 mg, 0.3 mmol) was dissolved in methanol (1 mL). 4-methoxybenzaldehyde (42.2 mg, 0.3 mmol) and piperidine (30.0 μL, 0.36 mmol) was added to reaction mixture sequentially. The reaction mixture was refluxed at 80 °C and stirred for 8 h. After completion of the reaction, the reaction mixture was cooled to room temperature (15-25 °C), and then the reaction mixture was concentrated under reduced pressure. The residue was dissolved in EA (50 mL), and H_2O (30 mL). The solution was transferred to separatory funnel, organic layers were separated. The organic layer was dried over Na_2SO_4, and filtered. The solvent was removed under reduced pressure and residue was purified by prep-LC (ACN / H_2O, 10% ACN to 100% ACN for 1 h, linear gradient) to give the yellow solid (20.4 mg, yield = 26.7%). ^{1}H NMR (400 MHz, $CDCl_3$) δ 8.66 (s, 1H), 7.79 (s, 1H), 7.75 (d, *J* = 7.7 Hz, 1H), 7.67 (d, *J* = 8.6 Hz, 2H), 7.20 (td, *J* = 7.75, 1.2 Hz, 1H), 6.99 (d, *J* = 8.7 Hz, 2H), 6.92-6.88 (m, 2H), 3.89 (s, 3H). ^{13}C NMR (125 MHz, $CDCl_3$) δ 170.7, 161.1, 141.5, 137.9, 131.7, 129.6, 127.3, 125.8, 122.8, 122.2, 121.9, 114.3, 110.3, 55.6. MS (m/z): $[M + H]^+$ calcd for $C_{16}H_{13}NO_2$, 252.09; found 252.1.

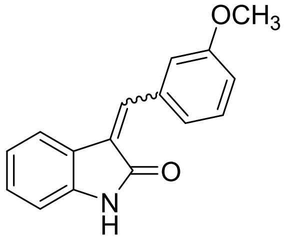

3-(3-methoxybenzylidene)indolin-2-one(OIC5)

Indolin-2-one (39.0 mg, 0.3 mmol) was dissolved in methanol (1 mL). 3-methoxybenzaldehyde (42.2 mg, 0.3 mmol) and piperidine (30.0 μL, 0.36 mmol) was added to reaction mixture sequentially. The reaction mixture was refluxed at 80 °C and stirred for 8 h. After completion of the reaction, the reaction mixture was cooled to room temperature (15-25 °C), and then the reaction mixture was concentrated under reduced pressure. The residue was dissolved in EA (50 mL), and H_2O (30 mL). The solution was transferred to separatory funnel, organic layers were separated. The organic layer was dried over Na_2SO_4, and filtered. The solvent was removed under reduced pressure and residue was purified by column chromatography to give the yellow solid (22.7 mg, yield = 30%). ^{1}H NMR (400 MHz, $CDCl_3$) δ 8.39 (s, 1H), 7.81 (s, 1H), 7.67 (d, *J* = 7.7 Hz, 1H), 7.39 (t, *J* = 7.85 Hz, 1H), 7.24-7.17 (m, 3H), 6.98 (dd, *J* = 8.25, 2.7 Hz, 1H), 6.90-6.96 (m, 2H), 3.84 (s, 3H). ^{13}C NMR (125 MHz, $CDCl_3$) δ 170.2, 159.8, 141.7, 137.6, 136.3, 130.1, 129.9, 123.5, 122.0, 121.9, 121.8, 115.8, 114.4, 110.3, 55.5. MS (m/z): $[M + H]^+$ calcd for $C_{16}H_{13}NO_2$, 252.09; found 252.1.

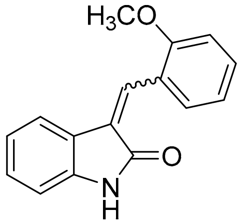

3-(2-methoxybenzylidene)indolin-2-one(OIC6)

Indolin-2-one (39.0 mg, 0.3 mmol) was dissolved in methanol (1 mL). 2-methoxybenzaldehyde (42.2 mg, 0.3 mmol) and piperidine (30.0 µL, 0.36 mmol) was added to reaction mixture sequentially. The reaction mixture was refluxed at 80 °C and stirred for 8 h. After completion of the reaction, the reaction mixture was cooled to room temperature (15-25 °C), and then the reaction mixture was concentrated under reduced pressure. The residue was dissolved in EA (50 mL), and H_2O (30 mL). The solution was transferred to separatory funnel, organic layers were separated. The organic layer was dried over Na_2SO_4, and filtered. The solvent was removed under reduced pressure and residue was purified by prep-LC (ACN / H_2O, 10% ACN to 100% ACN for 1 h, linear gradient) to give the yellow solid (21.8 mg, yield = 29%). ^{1}H NMR (400 MHz, $CDCl_3$) δ 8.25 (s, 1H), 7.97 (s, 1H), 7.73 (dd, J = 7.5, 1.7 Hz, 1H), 7.56 (d, J = 7.65 Hz, 1H), 7.43 (td, J = 7.9, 1.75 Hz, 1H), 7.19 (td, J = 7.65, 1.15 Hz, 1H), 7.03 (td, J = 7.5, 1 Hz, 1H), 6.98 (dd, J = 8.25, 1 Hz, 1H), 6.88 (d, J = 7.75, 1H), 6.85 (td, J = 7.65, 1.05 Hz, 1H). ^{13}C NMR (100 MHz, $CDCl_3$) δ 170.2, 158.3, 141.4, 134.2, 131.7, 130.1, 129.7, 127.2, 123.9, 123.2, 122.2, 121.8, 120.3, 111.1, 110.1, 55.7. MS (m/z): $[M + H]^+$ calcd for $C_{16}H_{13}NO_2$, 252.09; found 252.1.

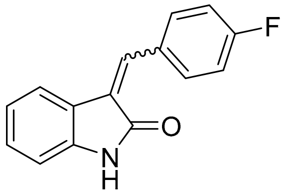

3-(4-fluorobenzylidene)indolin-2-one(OIC7)

Indolin-2-one (39.0 mg, 0.30 mmol) was dissolved in methanol (1 mL). 4-fluorobenzaldehyde (52.2 mg, 0.42 mmol) and 50% NaOH was added to reaction mixture sequentially. The reaction mixture was refluxed at 80 °C and stirred for 8 h. After completion of the reaction, the reaction mixture was cooled to room temperature (15-25 °C), and then the reaction mixture was concentrated under reduced pressure. The residue was dissolved in EA (50 mL), and H_2O (30 mL). The solution was transferred to separatory funnel, organic layers were separated. The organic layer was dried over Na_2SO_4, and filtered. The solvent was removed under reduced pressure and residue was purified by column chromatography to give the yellow solid (22.3 mg, yield = 30.1%). ^{1}H NMR (500 MHz, $CDCl_3$) δ 9.05 (s, 1H), 8.33 (t, J = 5.8 Hz, 1H), 7.78 (s, 1H), 7.66 (t, J = 5.4 Hz, 2H), 7.59 (d, J = 7.7 Hz, 1H), 7.50 (t, J = 4.35 Hz, 1H), 7.22 (td, J = 7.8, 1.3 Hz, 1H), 7.16 (t, J = 8.75 Hz, 2H), 6.92 (d, J = 7.8 Hz, 1H), 6.89 (t, J = 7.65 Hz, 1H). ^{13}C NMR (125 MHz, $CDCl_3$) δ 170.5, 162.4, 141.9, 139.9, 136.4, 131.6, 131.5, 130.2, 123.0, 122.0, 116.1, 115.9, 110.6. MS (m/z): $[M + H]^+$ calcd for $C_{15}H_{10}FNO$, 240.07; found 240.1.

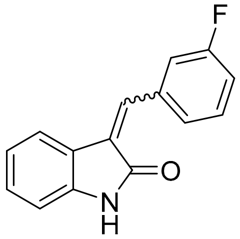

3-(3-fluorobenzylidene)indolin-2-one(OIC8)

Indolin-2-one (39.0 mg, 0.30 mmol) was dissolved in methanol (1 mL). 3-fluorobenzaldehyde (52.2 mg, 0.42 mmol) and piperidine (30.0 µL, 0.36 mmol) was added to reaction mixture sequentially. The reaction mixture was refluxed at 80 °C and stirred for 8 h. After completion of the reaction, the reaction mixture was cooled to room temperature (15-25 °C), and then the reaction mixture was concentrated under reduced pressure. The residue was dissolved in EA (50 mL), and H_2O (30 mL). The solution was transferred to separatory funnel, organic layers were separated. The organic layer was dried over Na_2SO_4, and filtered. The solvent was removed under reduced pressure and residue was purified by prep-LC (ACN / H_2O, 10% ACN to 100% ACN for 1 h, linear gradient) to give the yellow solid (26.8 mg, yield = 36.7%). ^{1}H NMR (400 MHz, $CDCl_3$) δ 8.23 (s, 1H), 7.75 (s, 1H), 7.58 (d, *J* = 7.55 Hz, 1H), 7.45-7.43 (m, 2H), 7.36-7.33 (m, 1H), 7.23 (td, *J* = 7.7, 1.15 Hz, 1H), 7.15-7.11 (m, 1H), 6.89 (t, *J* = 7.75 Hz, 2H). ^{13}C NMR (100 MHz, $CDCl_3$) δ 169.8, 163.9, 161.9, 141.7, 137.1, 135.8, 130.4, 128.5, 125.1, 123.3, 122.2, 121.5, 116.8, 116.2, 110.4. MS (m/z): $[M + H]^+$ calcd for $C_{15}H_{10}FNO$, 240.08; found 240.1.

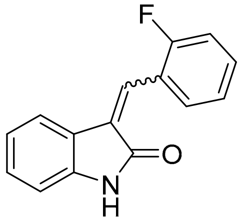

3-(2-fluorobenzylidene)indolin-2-one(OIC9)

Indolin-2-one (39.0 mg, 0.30 mmol) was dissolved in methanol (1 mL). 2-fluorobenzaldehyde (52.2 mg, 0.42 mmol) and piperidine (30.0 µL, 0.36 mmol) was added to reaction mixture sequentially. The reaction mixture was refluxed at 80 °C and stirred for 8 h. After completion of the reaction, the reaction mixture was cooled to room temperature (15-25 °C), and then the reaction mixture was concentrated under reduced pressure. The residue was dissolved in EA (50 mL), and H_2O (30 mL). The solution was transferred to separatory funnel, organic layers were separated. The organic layer was dried over Na_2SO_4, and filtered. The solvent was removed under reduced pressure and residue was purified by column chromatography to give the yellow solid (21.5 mg, 0.08 mmol, yield = 26.7%). ^{1}H NMR (400 MHz, $CDCl_3$) δ 8.17 (s, 1H), 7.81 (s, 1H), 7.71 (t, *J* = 7.5 Hz, 1H), 7.44 (d, *J* = 7.5 Hz, 2H), 7.24-7.17 (m, 3H), 6.89-6.84 (m, 2H). ^{13}C NMR (100 MHz, $CDCl_3$) δ 169.5, 161.7, 141.8, 131.8, 130.5, 130.4, 129.9, 124.2, 123.5, 122.1, 121.7, 120.1, 116.4, 110.3, 109.7. MS (m/z): $[M + H]^+$ calcd for $C_{15}H_{10}FNO$, 240.08; found 240.1.

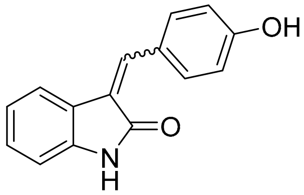

3-(4-hydroxybenzylidene)indolin-2-one(OIC10)

Indolin-2-one (39.0 mg, 0.3 mmol) was dissolved in methanol (1 mL). 4-hydroxybenzaldehyde (36.6 mg, 0.3 mmol) and piperidine (30.0 µL, 0.36 mmol) was added to reaction mixture sequentially. The reaction mixture was refluxed at 80 °C and stirred for 8 h. After completion of the reaction, the reaction mixture was cooled to room temperature (15-25 °C), and then the reaction mixture was concentrated under reduced pressure. The residue was dissolved in EA (50 mL), and H_2O (30 mL). The solution was transferred to separatory funnel, organic layers were separated. The organic layer was dried over Na_2SO_4, and filtered. The solvent was removed under reduced pressure and residue was purified by column chromatography to give the yellow solid (24.6 mg, 0.10 mmol, yield = 33.3%). ^{1}H NMR (500 MHz, DMSO-d_6) δ 10.55 (s, 1H), 7.68 (s, 1H), 7.60 (dd, *J* = 7.75, 1.75 Hz, 1H), 7.48 (d, *J* = 7.15 Hz, 1H), 7.30 (td, *J* = 6.75, 1.75 Hz, 1H), 7.20 (td, *J* = 7.75, 1.15 Hz, 1H), 6.96 (d, *J* = 8.25, 1H), 6.91 (td, *J* = 7.5, 1.1 Hz, 1H), 6.87 (d, *J* = 8 Hz, 1H), 6.84 (td, *J* = 7.65, 1.1 Hz, 1H). ^{13}C NMR (125 MHz, DMSO-d_6) δ 169.1,

156.7, 142.7, 132.8, 131.9, 129.9, 129.8, 126.7, 122.5, 121.5, 121.5, 121.3, 119.1, 116.2, 110.2. MS (m/z): [M + H]$^+$ calcd for $C_{15}H_{11}NO_2$, 238.08; found 238.1.

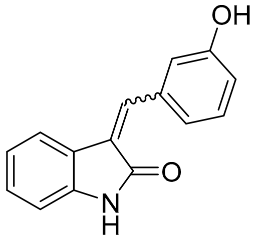

3-(3-hydroxybenzylidene)indolin-2-one(OIC11)

Indolin-2-one (39.0 mg, 0.3 mmol) was dissolved in methanol (1 mL). 3-hydroxybenzaldehyde (36.6 mg, 0.3 mmol) and piperidine (30.0 µL, 0.36 mmol) was added to reaction mixture sequentially. The reaction mixture was refluxed at 80 °C and stirred for 8 h. After completion of the reaction, the reaction mixture was cooled to room temperature (15-25 °C), and then the reaction mixture was concentrated under reduced pressure. The residue was dissolved in EA (50 mL), and H_2O (30 mL). The solution was transferred to separatory funnel, organic layers were separated. The organic layer was dried over Na_2SO_4, and filtered. The solvent was removed under reduced pressure and residue was purified by column chromatography to give the yellow solid (23.4 mg, 0.10 mmol, yield = 33.3%). ^{1}H NMR (400 MHz, DMSO-d_6) δ 10.60 (s, 1H), 7.68 (s, 1H), 7.57 (d, J = 7.65 Hz, 1H), 7.53 (s, 1H), 7.31 (t, J = 7.85 Hz, 1H), 7.24-7.20 (m, 2H), 7.08 (t, J = 8.3 Hz, 2H), 6.88-6.84 (m, 2H). ^{13}C NMR (100 MHz, DMSO-d_6) δ 169.0, 157.6, 143.0, 136.3, 135.8, 130.4, 130.2, 127.6, 122.8, 121.4, 121.1, 120.4, 117.1, 115.7, 110.4. MS (m/z): [M + H]$^+$ calcd for $C_{15}H_{11}NO_2$, 238.08; found 238.1.

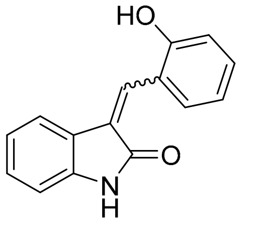

3-(2-hydroxybenzylidene)indolin-2-one(OIC12)

Indolin-2-one (39.0 mg, 0.3 mmol) was dissolved in methanol (1 mL). 2-hydroxybenzaldehyde (36.6 mg, 0.3 mmol) and piperidine (30.0 µL, 0.36 mmol) was added to reaction mixture sequentially. The reaction mixture was refluxed at 80 °C and stirred for 8 h. After completion of the reaction, the reaction mixture was cooled to room temperature (15-25 °C), and then the reaction mixture was concentrated under reduced pressure. The residue was dissolved in EA (50 mL), and H_2O (30 mL). The solution was transferred to separatory funnel, organic layers were separated. The organic layer was dried over Na_2SO_4, and filtered. The solvent was removed under reduced pressure and residue was purified by prep-LC (ACN / H_2O, 10% ACN to 100% ACN for 1 h, linear gradient) to give the yellow solid (26.6 mg, 0.11 mmol, yield = 36.7%). ^{1}H NMR (400 MHz, DMSO-d_6) δ 10.52 (s, 1H), 7.68 (s, 1H), 7.60 (d, J = 7.65 Hz, 1H), 7.49 (m, 1H), 7.25 (m, 1H), 7.20 (m.1H), 6.90-6.86 (m, 3H), 6.81 (t, J = 7.6 Hz, 1H). ^{13}C NMR (100 MHz, DMSO-d_6) δ 169.4, 159.6, 142.6, 136.9, 135.0, 132.1, 129.7, 125.1, 124.8, 122.2, 121.5, 121.3, 115.9, 115.4, 110.2. MS (m/z): [M + H]$^+$ calcd for $C_{15}H_{11}NO_2$, 238.08; found 238.1.

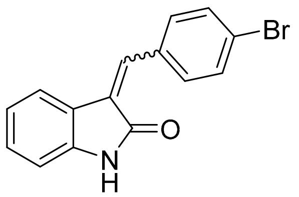

3-(4-bromobenzylidene) indolin-2-one(OIC13)

Indolin-2-one (39.0 mg, 0.3 mmol) was dissolved in methanol (1 mL). 4-bromobenzaldehyde (55.2 mg, 0.3 mmol) and piperidine (30.0 µL, 0.36 mmol) was added to reaction mixture sequentially. The reaction mixture was refluxed at 80 °C and stirred for 8 h. After completion of the reaction, the reaction mixture was cooled to room temperature (15-25 °C), and then the reaction mixture was concentrated under reduced pressure. The residue was dissolved in EA (50 mL), and H_2O (30 mL). The solution was transferred to separatory funnel, organic layers were separated. The organic layer was dried over Na_2SO_4, and filtered. The solvent was removed under reduced pressure and residue was purified by column chromatography to give the yellow solid (25.7 mg, yield = 28.7%). ^1H NMR (400 MHz, $CDCl_3$) δ 8.38 (s, 1H), 8.15 (d, J = 8.35 Hz, 1H), 7.72 (s, 1H), 7.61 (m, 4H), 7.57 (d, J = 7.95 Hz, 2H), 7.23 (t, J = 7.8 Hz, 1H), 6.90-6.88 (m, 2H). ^{13}C NMR (100 MHz, $CDCl_3$) δ 170.0, 142.0, 136.1, 134.2, 132.4, 131.3, 130.7, 124.3, 123.5, 122.4, 110.9. MS (m/z): $[M + H]^+$ calcd for $C_{15}H_{10}BrNO$, 299.99; found 300.0.

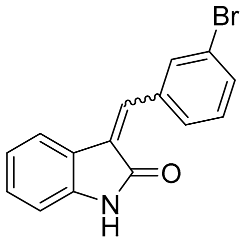

3-(3-bromobenzylidene) indolin-2-one(OIC14)

Indolin-2-one (39.0 mg, 0.3 mmol) was dissolved in methanol (1 mL). 3-bromobenzaldehyde (55.2 mg, 0.3 mmol) and piperidine (30.0 µL, 0.36 mmol) was added to reaction mixture sequentially. The reaction mixture was refluxed at 80 °C and stirred for 8 h. After completion of the reaction, the reaction mixture was cooled to room temperature (15-25 °C), and then the reaction mixture was concentrated under reduced pressure. The residue was dissolved in EA (50 mL), and H_2O (30 mL). The solution was transferred to separatory funnel, organic layers were separated. The organic layer was dried over Na_2SO_4, and filtered. The solvent was removed under reduced pressure and residue was purified by prep-LC (ACN / H_2O, 10% ACN to 100% ACN for 1 h, linear gradient) to give the yellow solid (20.7 mg, yield = 23.7%). ^1H NMR (400 MHz, $CDCl_3$) δ 8.86 (s, 1H), 7.68 (s, 1H), 7.62 (s, 1H), 7.47-7.42 (m, 3H), 7.24 (t, J = 7.9 Hz, 1H), 7.13 (t, J = 7.7 Hz, 1H), 6.83-6.77 (m, 2H). ^{13}C NMR (100 MHz, $CDCl_3$) δ 169.9, 141.9, 137.1, 135.5, 132.6, 132.0, 130.5, 130.4, 128.8, 127.9, 123.3, 122.9, 122.2, 121.5, 110.5. MS (m/z): $[M + H]^+$ calcd for $C_{15}H_{10}BrNO$, 299.99; found 300.0.

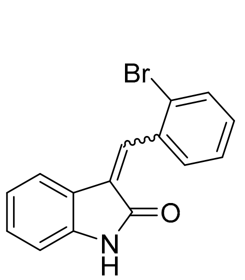

3-(2-bromobenzylidene)indolin-2-one(OIC15)

Indolin-2-one (39.0 mg, 0.3 mmol) was dissolved in methanol (1 mL). 2-bromobenzaldehyde (55.2 mg, 0.3 mmol) and piperidine (30.0 µL, 0.36 mmol) was added to reaction mixture sequentially. The reaction mixture was

refluxed at 80 °C and stirred for 8 h. After completion of the reaction, the reaction mixture was cooled to room temperature (15-25 °C), and then the reaction mixture was concentrated under reduced pressure. The residue was dissolved in EA (50 mL), and H_2O (30 mL). The solution was transferred to separatory funnel, organic layers were separated. The organic layer was dried over Na_2SO_4, and filtered. The solvent was removed under reduced pressure and residue was purified by prep-LC (ACN / H_2O, 10% ACN to 100% ACN for 1 h, linear gradient) to give the yellow solid (28.1 mg, yield = 31.3%). ^{1}H NMR (400 MHz, $CDCl_3$) δ 8.71 (s, 1H), 7.81 (s, 1H), 7.71 (d, J = 8.05 Hz, 2H), 7.40 (t, J = 7.5 Hz, 1H), 7.32-7.27 (m, 2H), 7.21 (t, J = 7.65 Hz, 1H), 6.92 (d, J = 7.75 Hz, 1H), 6.82 (t, J = 6.55 Hz, 1H). ^{13}C NMR (100 MHz, $CDCl_3$) δ 169.8, 141.9, 136.0, 135.6, 133.4, 131.0, 129.0, 127.4, 124.3, 123.4, 122.1, 121.5, 110.5. MS (m/z): $[M + H]^+$ calcd for $C_{15}H_{10}BrNO$, 299.99; found 300.0.

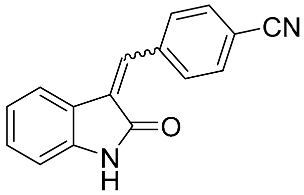

4-((2-oxoindolin-3-ylidene)methyl)benzonitrile(OIC16)

Indolin-2-one (39.0 mg, 0.3 mmol) was dissolved in methanol (1 mL). 4-formylbenzonitrile (39.3 mg, 0.3 mmol) and piperidine (30.0 μL, 0.36 mmol) was added to reaction mixture sequentially. The reaction mixture was refluxed at 80 °C and stirred for 8 h. After completion of the reaction, the reaction mixture was cooled to room temperature (15-25 °C), and then the reaction mixture was concentrated under reduced pressure. The residue was dissolved in EA (50 mL), and H_2O (30 mL). The solution was transferred to separatory funnel, organic layers were separated. The organic layer was dried over Na_2SO_4, and filtered. The solvent was removed under reduced pressure and residue was purified by prep-LC (ACN / H_2O, 10% ACN to 100% ACN for 1 h, linear gradient) to give the yellow solid (23.5 mg, yield = 31.7%). ^{1}H NMR (400 MHz, $CDCl_3$) δ 8.18 (s, 1H), 7.77-7.73 (m, 5H), 7.44 (d, J = 7.7 Hz, 1H), 7.26 (t, J = 6.65 Hz, 1H), 6.89 (t, J = 7.85 Hz, 2H). ^{13}C NMR (100 MHz, $CDCl_3$) δ 169.4, 142.1, 139.8, 134.3, 132.6, 131.1, 129.9, 123.3, 122.3, 121.1, 118.5, 113.0, 110.6. MS (m/z): $[M + H]^+$ calcd for $C_{16}H_{10}N_2O$, 247.08; found 247.1.

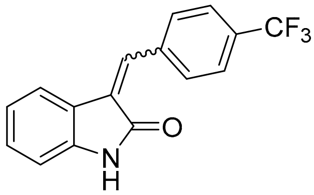

3-(4-(trifluoromethyl)benzylidene)indolin-2-one(OIC17)

Indolin-2-one (39.0 mg, 0.3 mmol) was dissolved in methanol (1 mL). 4-(trifluoromethyl) benzaldehyde (52.2 mg, 0.3 mmol) and piperidine (30.0 μL, 0.36 mmol) was added to reaction mixture sequentially. The reaction mixture was refluxed at 80 °C and stirred for 8 h. After completion of the reaction, the reaction mixture was cooled to room temperature (15-25 °C), and then the reaction mixture was concentrated under reduced pressure. The residue was dissolved in EA (50 mL), and H_2O (30 mL). The solution was transferred to separatory funnel, organic layers were separated. The organic layer was dried over Na_2SO_4, and filtered. The solvent was removed under reduced pressure and residue was purified by prep-LC (ACN / H_2O, 10% ACN to 100% ACN for 1 h, linear gradient) to give the yellow solid (24.0 mg, yield = 27.7%). ^{1}H NMR (400 MHz, $CDCl_3$) δ 8.86 (s, 1H), 7.80 (s, 1H), 7.75 (q, J = 8.45 Hz, 4H), 7.49 (d, J = 7.65 Hz, 1H), 7.24 (t, J = 5.7Hz, 1H), 6.93 (d, J = 7.8 Hz, 1H), 6.88 (td, J = 7.6, 1.05 Hz, 1H). ^{13}C NMR (100 MHz, $CDCl_3$) δ 169.7, 142.0, 138.7, 135.2, 130.7, 129.6, 129.3, 125.9, 123.3, 122.2, 121.3, 110.5 MS (m/z): $[M + H]^+$ calcd for $C_{16}H_{10}F_3NO$, 290.07; found 290.1.

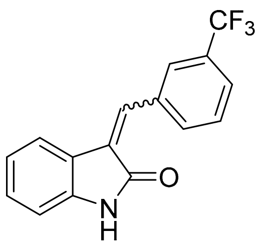

3-(3-(trifluoromethyl)benzylidene)indolin-2-one(OIC18)

Indolin-2-one (39.0 mg, 0.3 mmol) was dissolved in methanol (1 mL). 3-(trifluoromethyl) benzaldehyde (52.2 mg, 0.3 mmol) and piperidine (30.0 μL, 0.36 mmol) was added to reaction mixture sequentially. The reaction mixture was refluxed at 80 °C and stirred for 8 h. After completion of the reaction, the reaction mixture was cooled to room temperature (15-25 °C), and then the reaction mixture was concentrated under reduced pressure. The residue was dissolved in EA (50 mL), and H_2O (30 mL). The solution was transferred to separatory funnel, organic layers were separated. The organic layer was dried over Na_2SO_4, and filtered. The solvent was removed under reduced pressure and residue was purified by column chromatography to give the yellow solid (26.8 mg, yield = 31%). ^{1}H NMR (400 MHz, $CDCl_3$) δ 8.44 (s, 1H), 7.92 (s, 1H), 7.81 (t, *J* = 7.65 Hz, 2H), 7.69 (d, *J* = 7.85 Hz, 1H), 7.61 (t, *J* = 7.8 Hz, 1H), 7.47 (d, *J* = 7.7 Hz, 1H), 7.24 (t, *J* = 7.75 Hz, 1H), 6.92-6.86 (m, 2H). ^{13}C NMR (100 MHz, $CDCl_3$) δ 169.4, 142.1, 139.8, 134.3, 132.6, 131.1, 129.9, 123.3, 122.3, 121.1, 118.5, 113.0, 110.6. MS (m/z): $[M + H]^+$ calcd for $C_{16}H_{10}F_3NO$, 290.07; found 290.1.

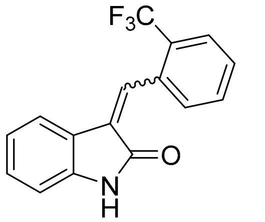

3-(2-(trifluoromethyl)benzylidene)indolin-2-one(OIC19)

Indolin-2-one (39.0 mg, 0.3 mmol) was dissolved in methanol (1 mL). 2-(trifluoromethyl) benzaldehyde (52.2 mg, 0.3 mmol) and piperidine (30.0 μL, 0.36 mmol) was added to reaction mixture sequentially. The reaction mixture was refluxed at 80 °C and stirred for 8 h. After completion of the reaction, the reaction mixture was cooled to room temperature (15-25 °C), and then the reaction mixture was concentrated under reduced pressure. The residue was dissolved in EA (50 mL), and H_2O (30 mL). The solution was transferred to separatory funnel, organic layers were separated. The organic layer was dried over Na_2SO_4, and filtered. The solvent was removed under reduced pressure and residue was purified by column chromatography to give the yellow solid (25.2 mg, yield = 29%). ^{1}H NMR (400 MHz, $CDCl_3$) δ 8.61 (s, 1H), 7.97 (s, 1H), 7.81 (d, *J* = 7.8 Hz, 1H), 7.71 (d, *J* = 7.55 Hz, 1H), 7.62 (t, *J* = 7.6 Hz, 1H), 7.56 (t, *J* = 8.4 Hz, 1H), 7.20 (t, *J* = 6.55 Hz, 1H), 6.96 (d, *J* = 7.7 Hz, 1H), 6.91 (d, *J* = 7.75 Hz, 1H), 6.77 (t, *J* = 7.6 Hz, 1H). ^{13}C NMR (100 MHz, $CDCl_3$) δ 169.5, 142.1, 133.4, 132.0, 130.5, 130.3, 129.3, 126.6, 123.5, 122.1, 121.4, 110.5. MS (m/z): $[M + H]^+$ calcd for $C_{16}H_{10}F_3NO$, 290.07; found 290.1.

^{1}H-NMR(CDCl3) of OIC1

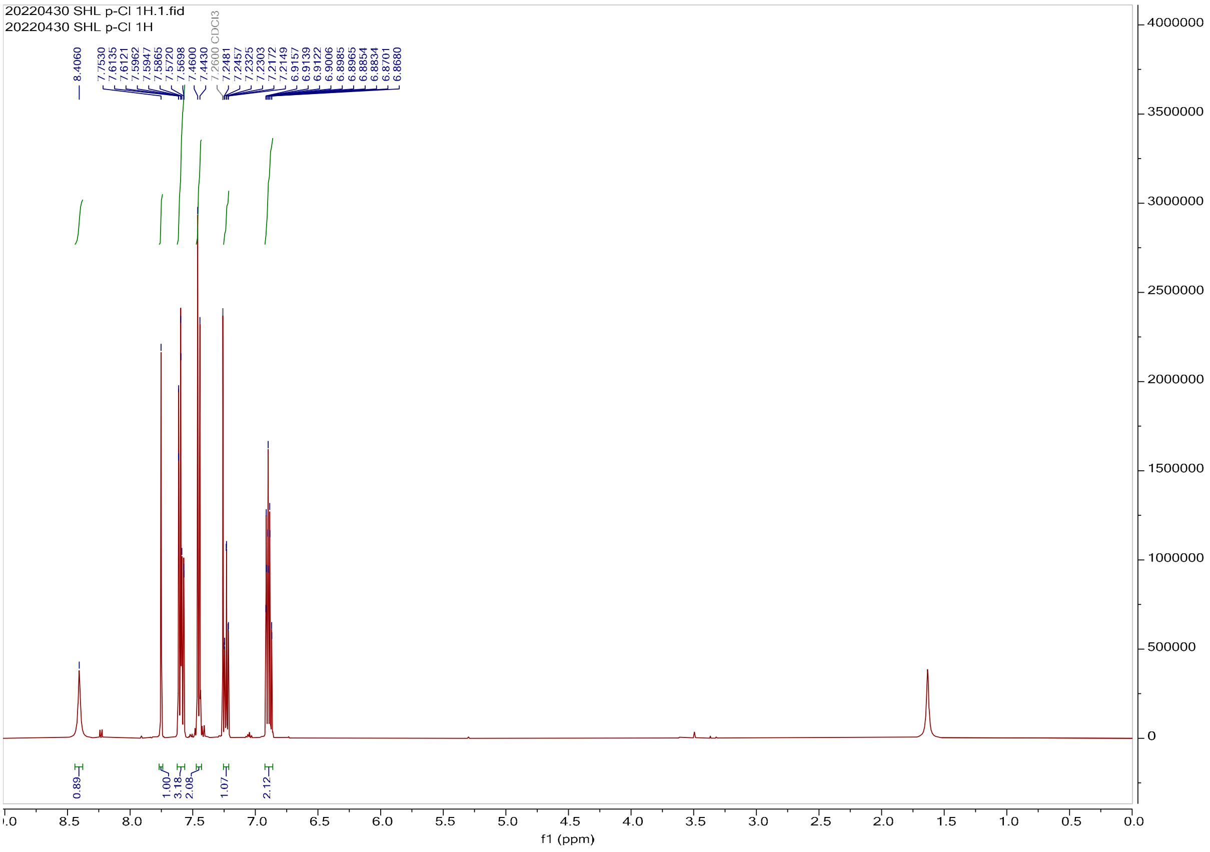

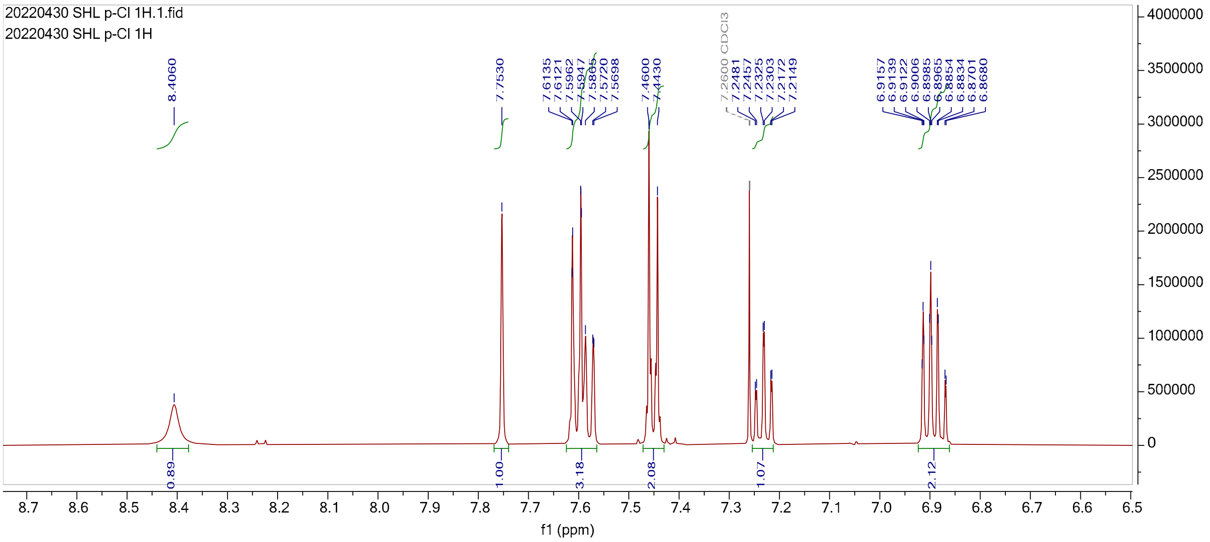

^{13}C-NMR($CDCl_3$) of OIC1

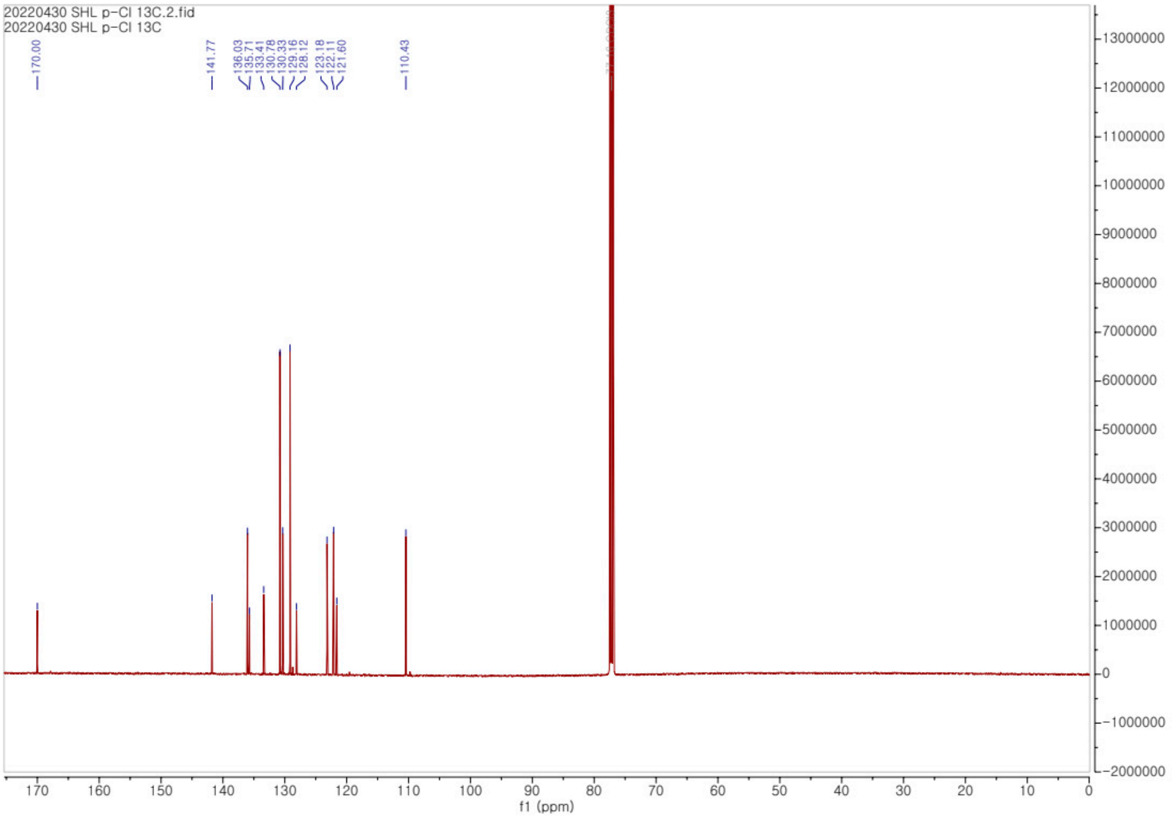

The mass data of OIC1

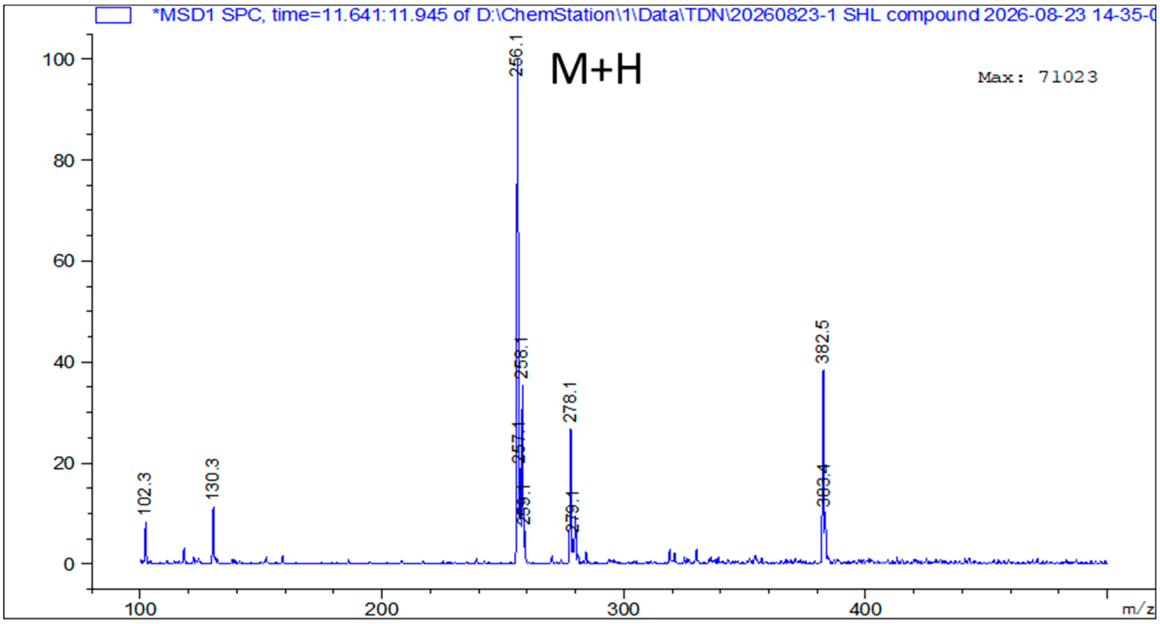

^{1}H-NMR($CDCl_3$) of OIC2

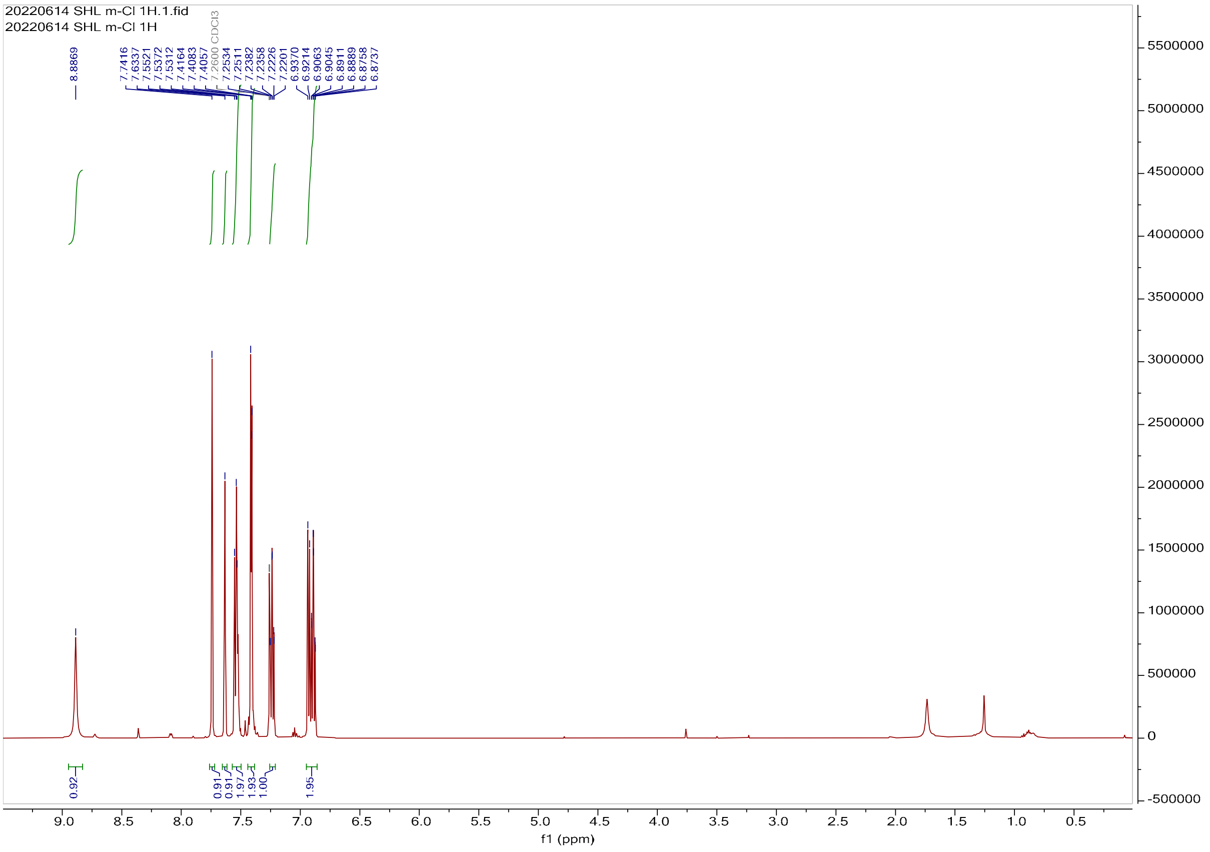

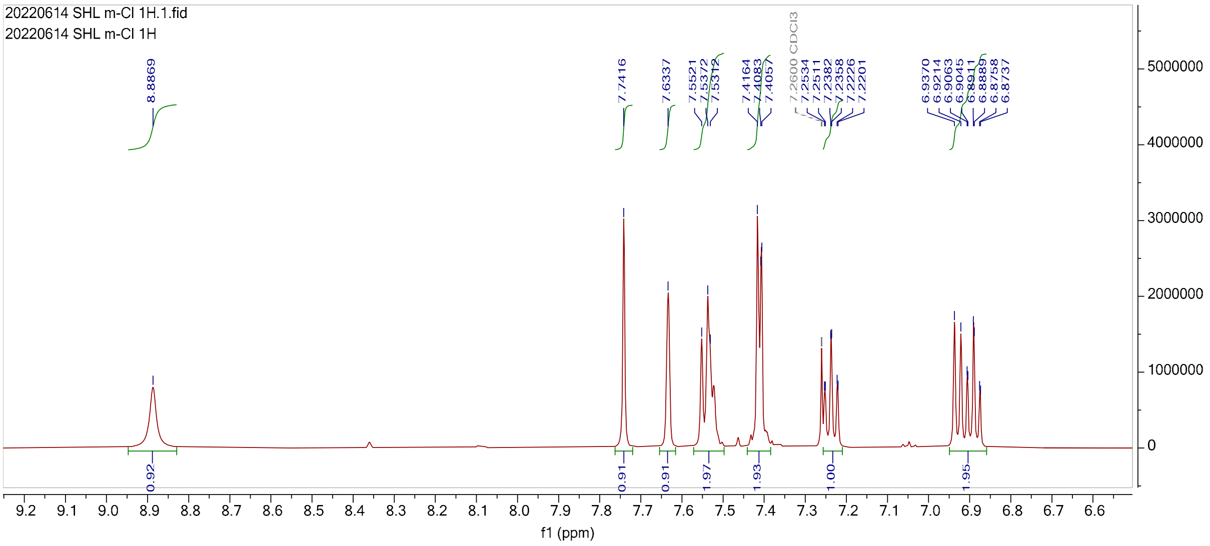

^{13}C-NMR($CDCl_3$) of OIC2

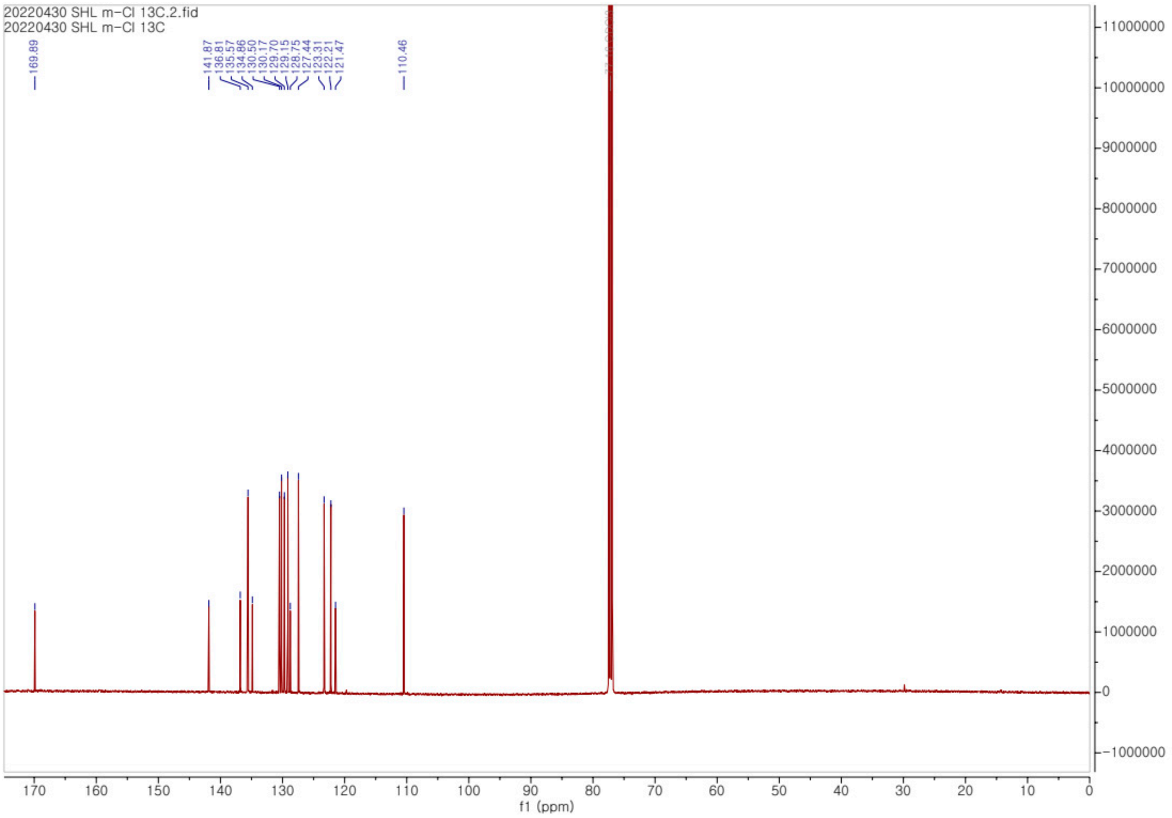

The mass data of OIC2

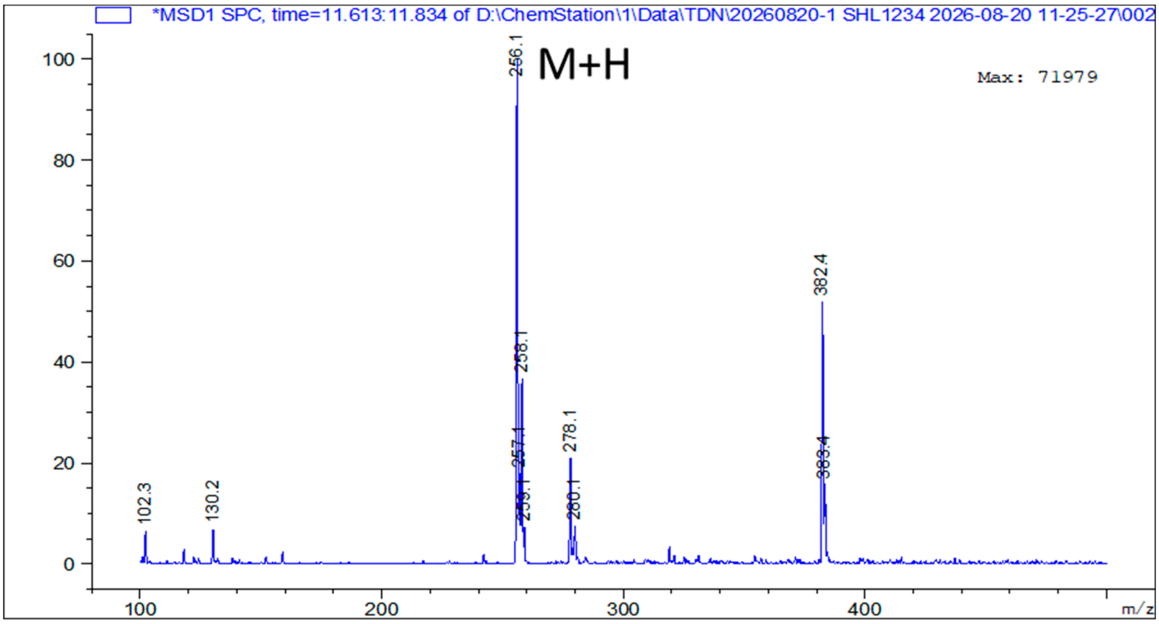

^{1}H-NMR($CDCl_3$) of OIC3

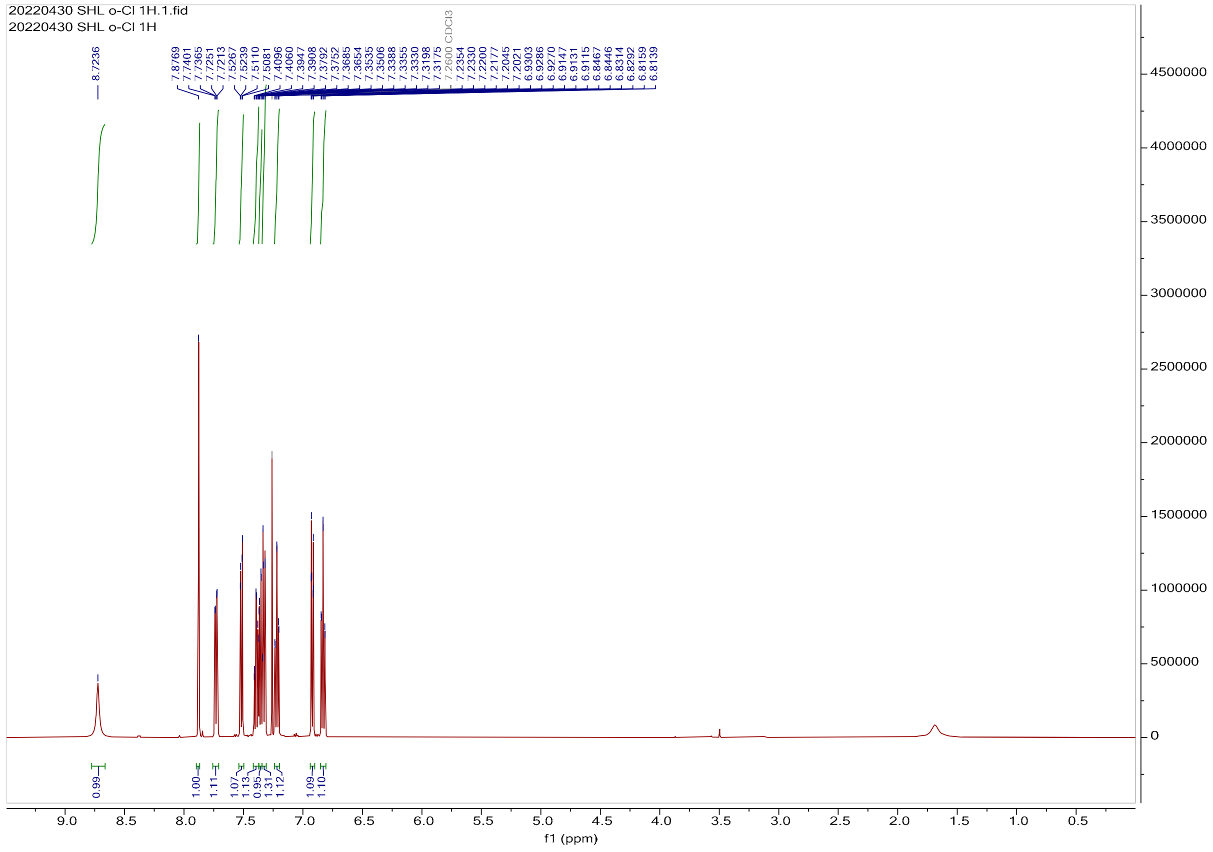

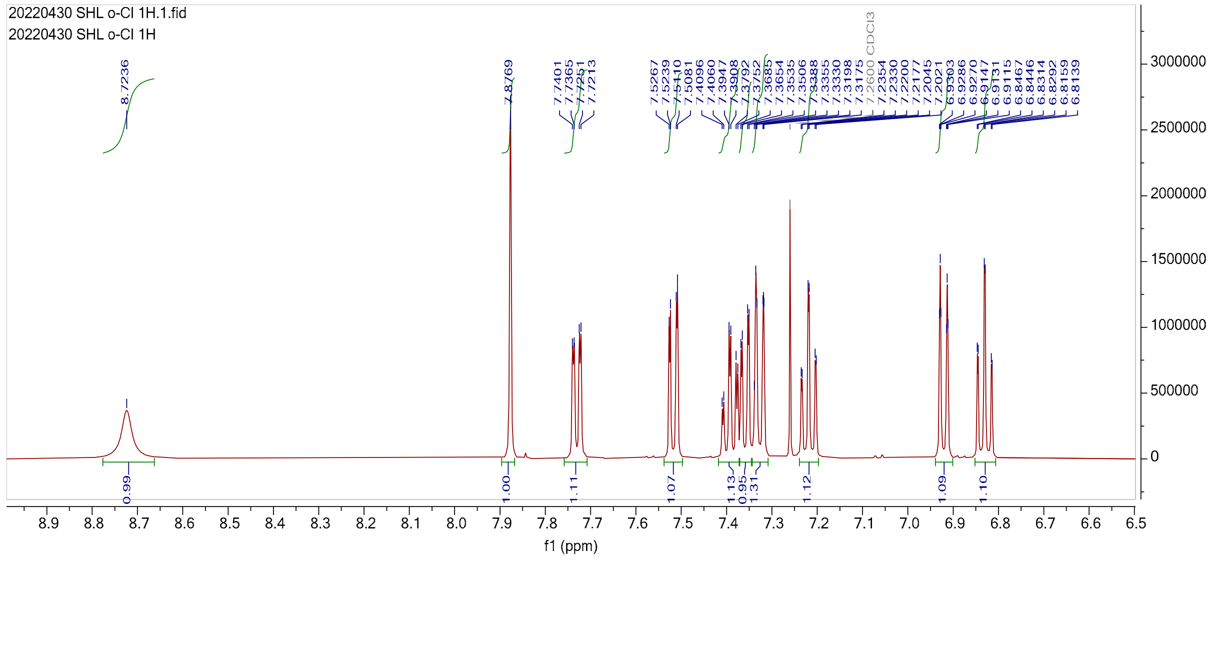

^{13}C-NMR($CDCl_3$) of OIC3

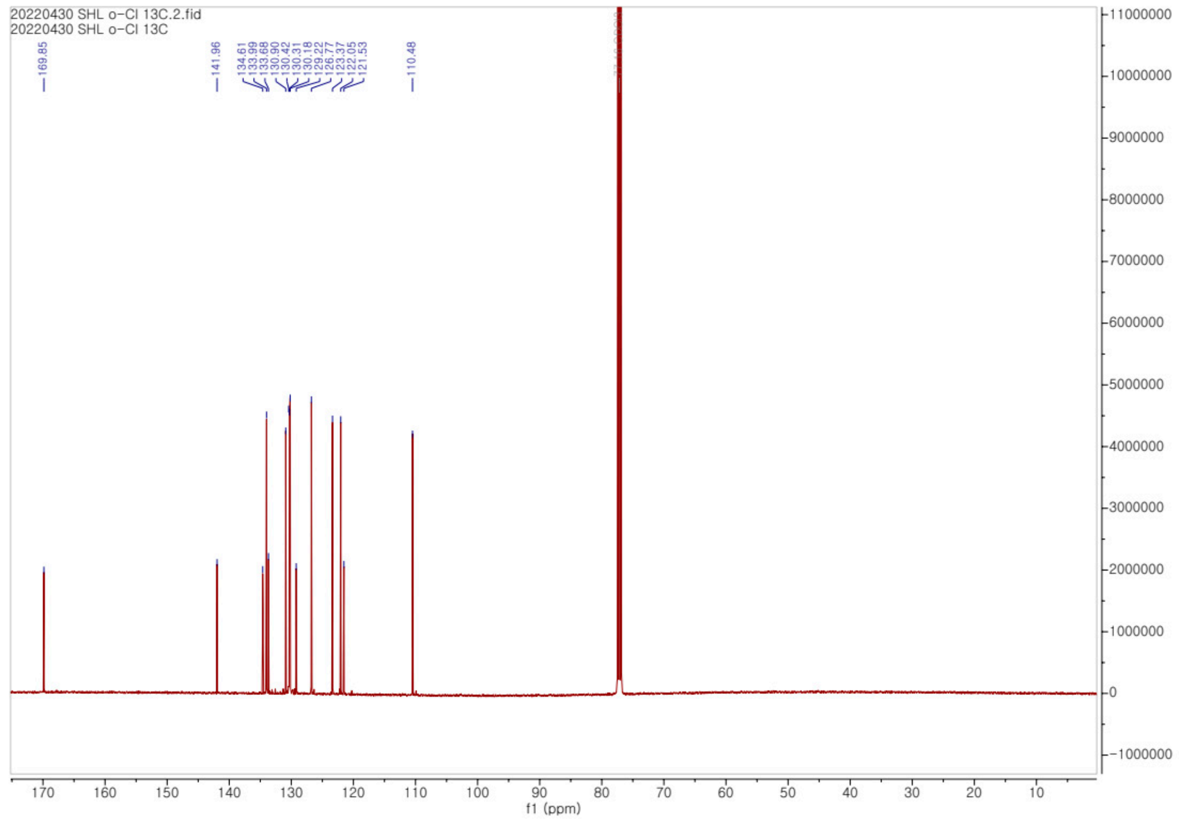

The mass data of OIC3

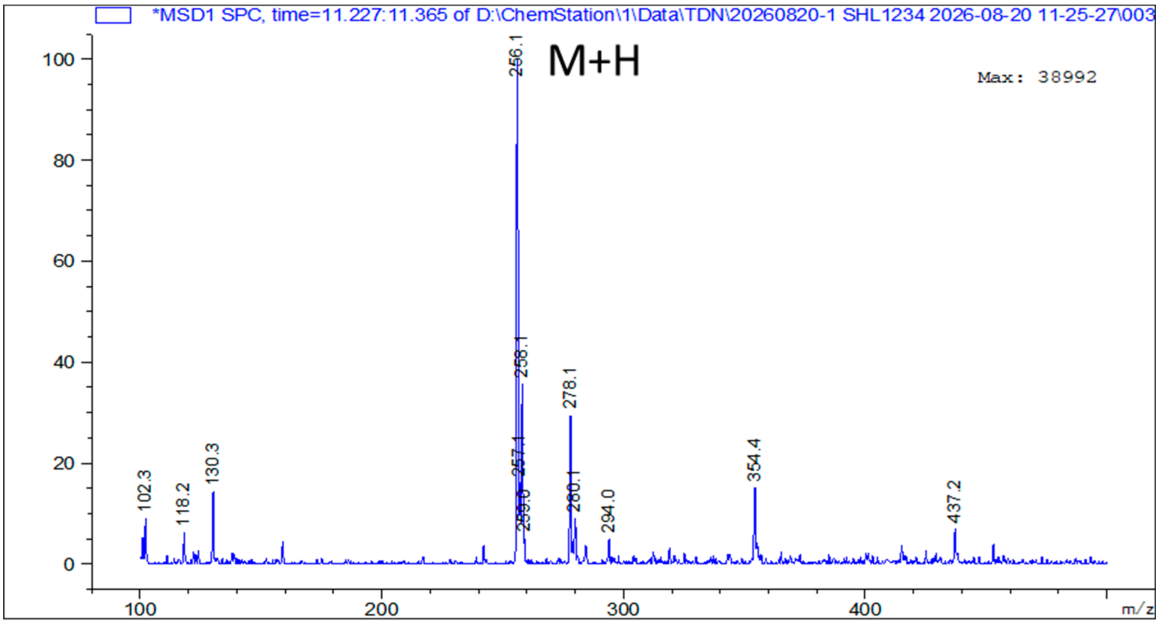

^{1}H-NMR(CDCl3) of OIC4

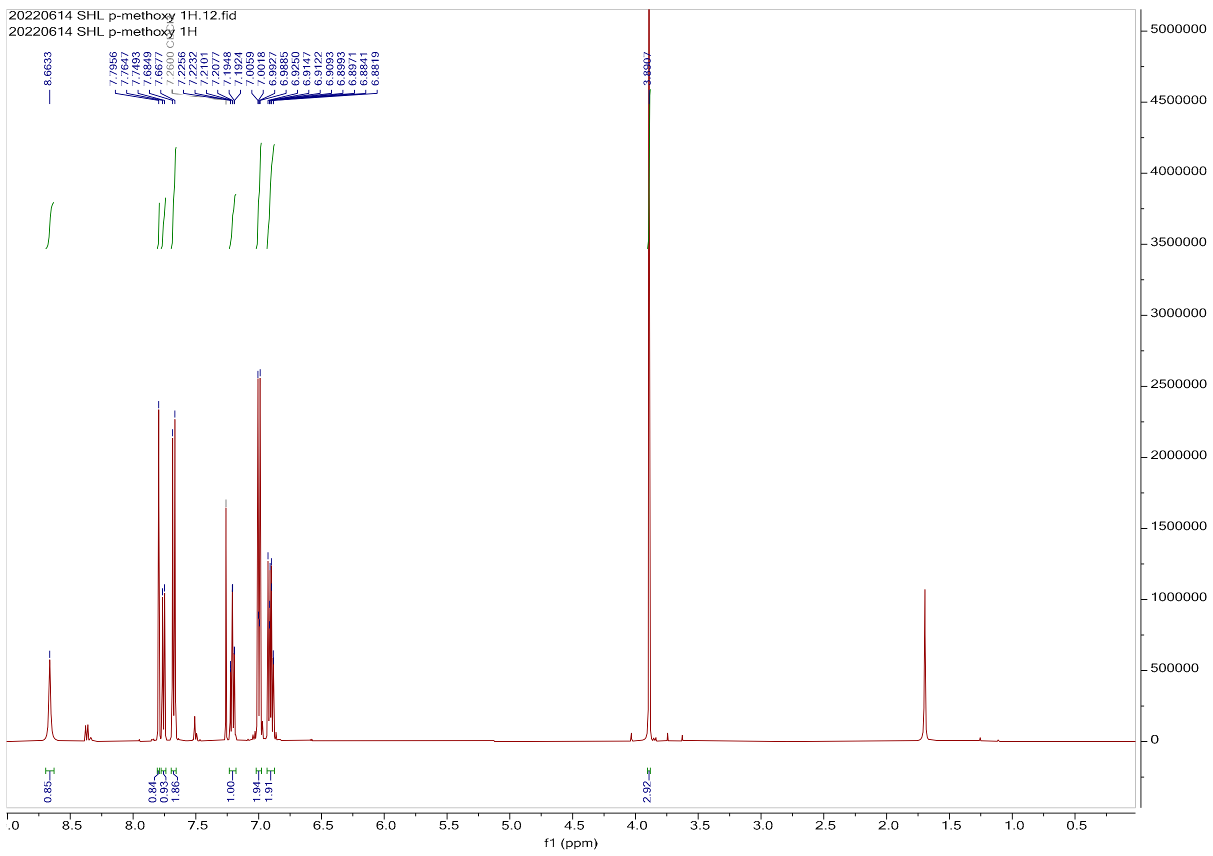

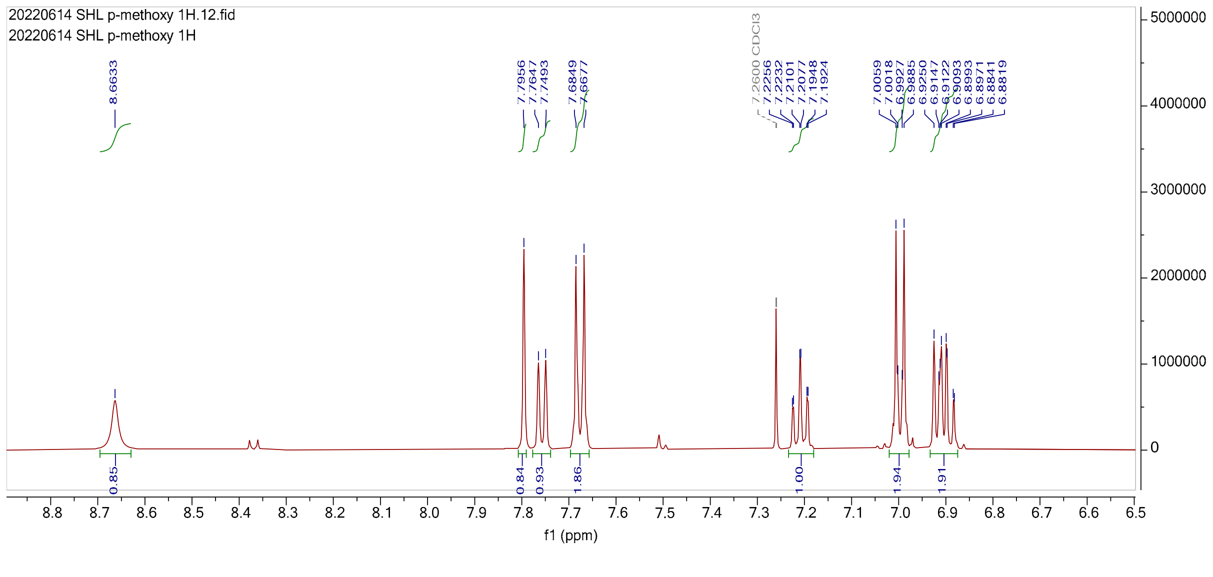

^{13}C-NMR($CDCl_3$) of OIC4

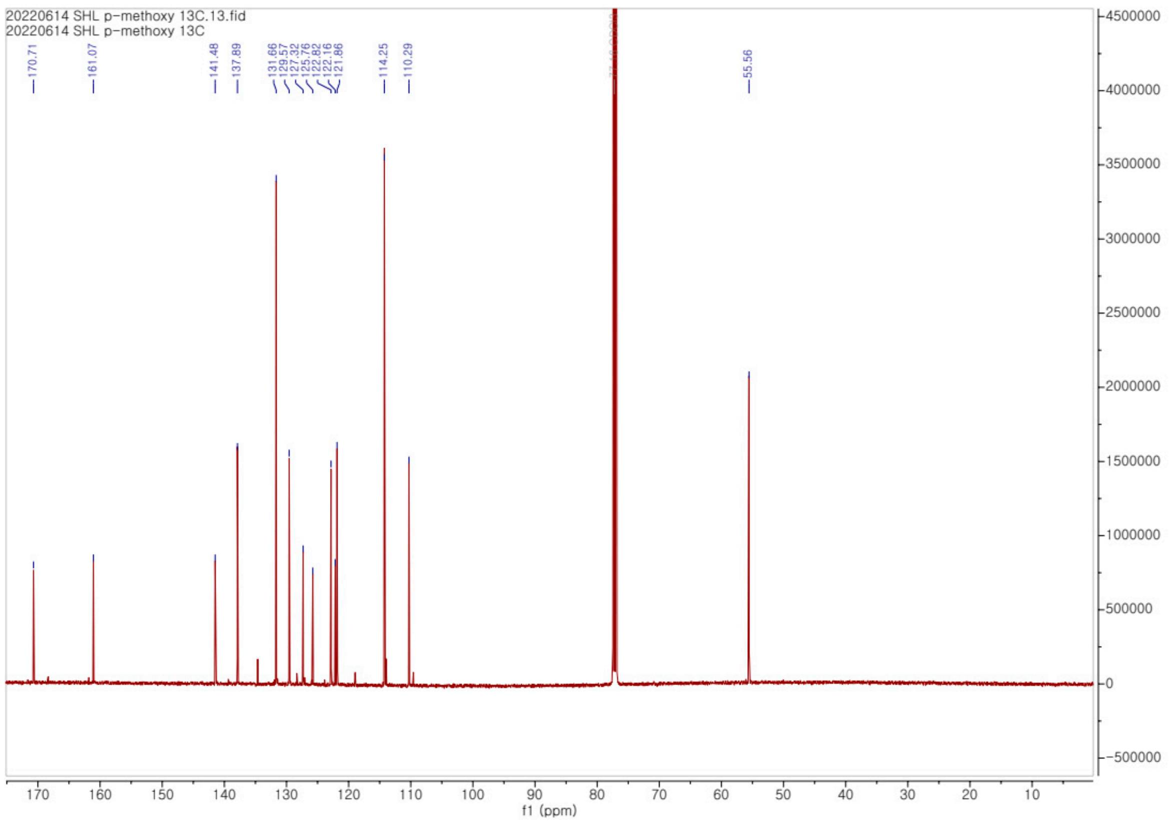

The mass data of OIC4

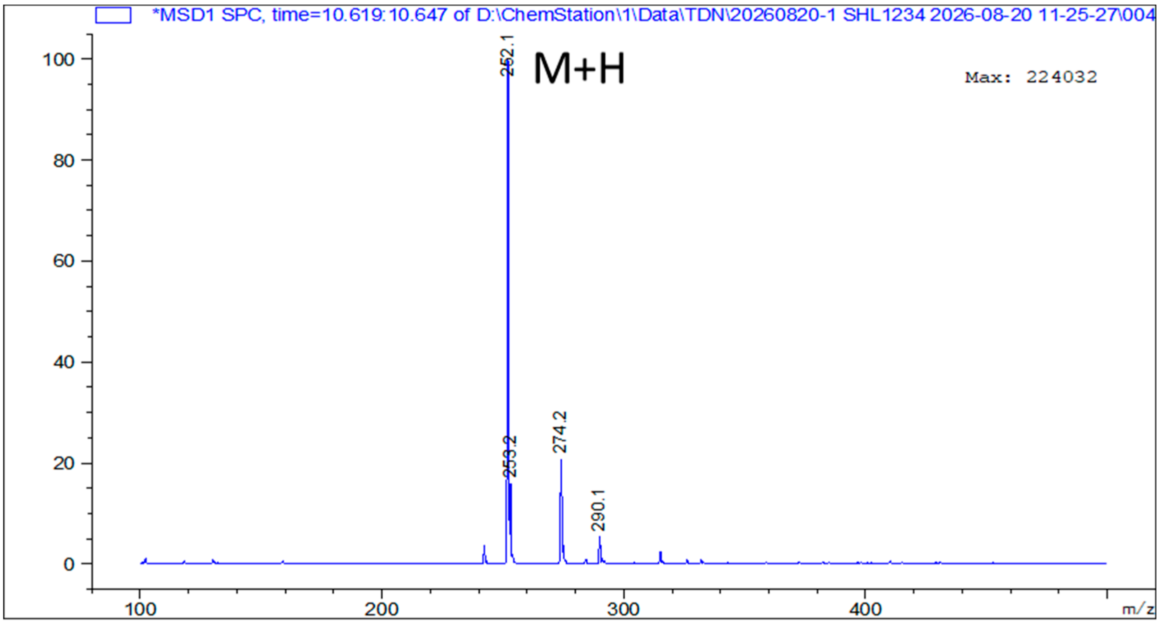

^{1}H-NMR ($CDCl_3$) of OIC5

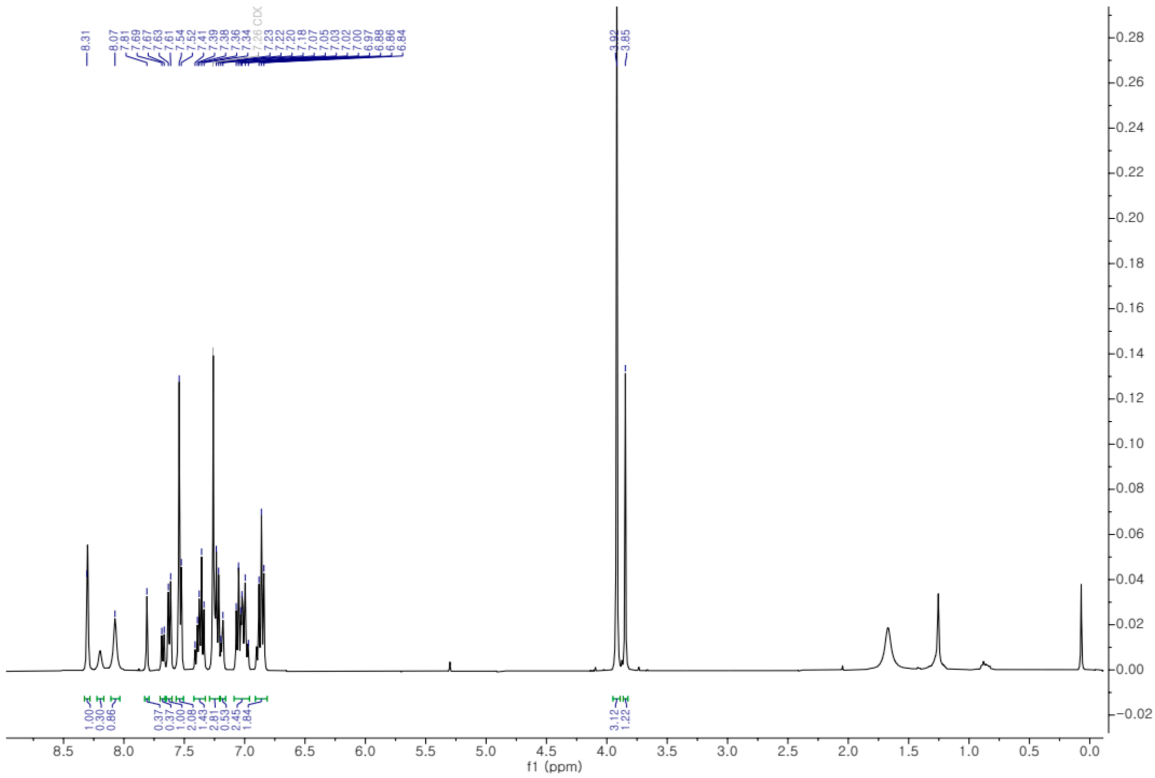

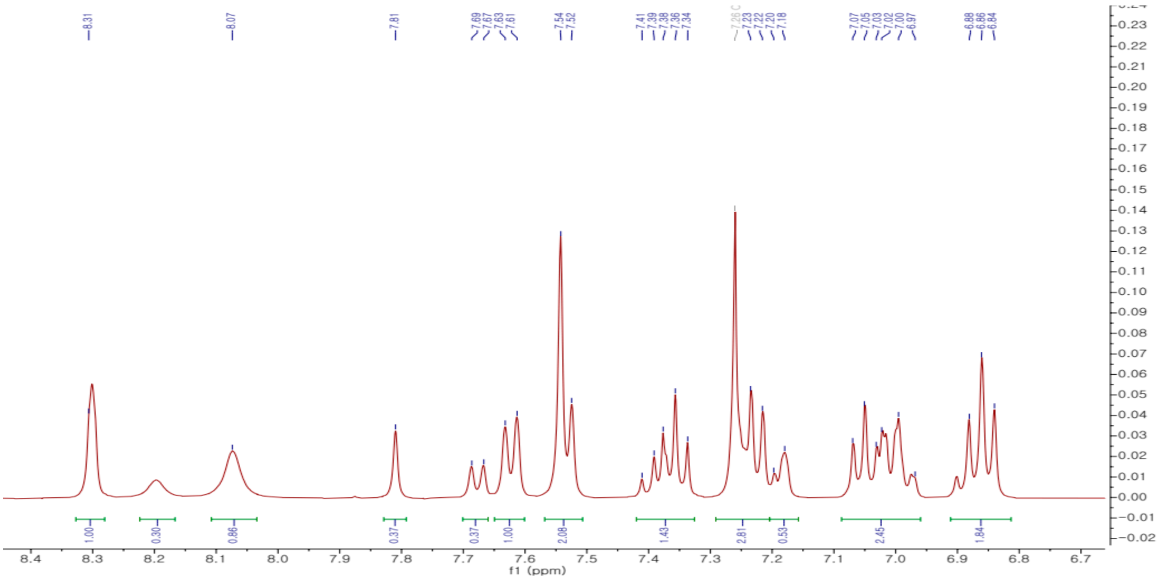

^{13}C-NMR ($CDCl_3$) of OIC5

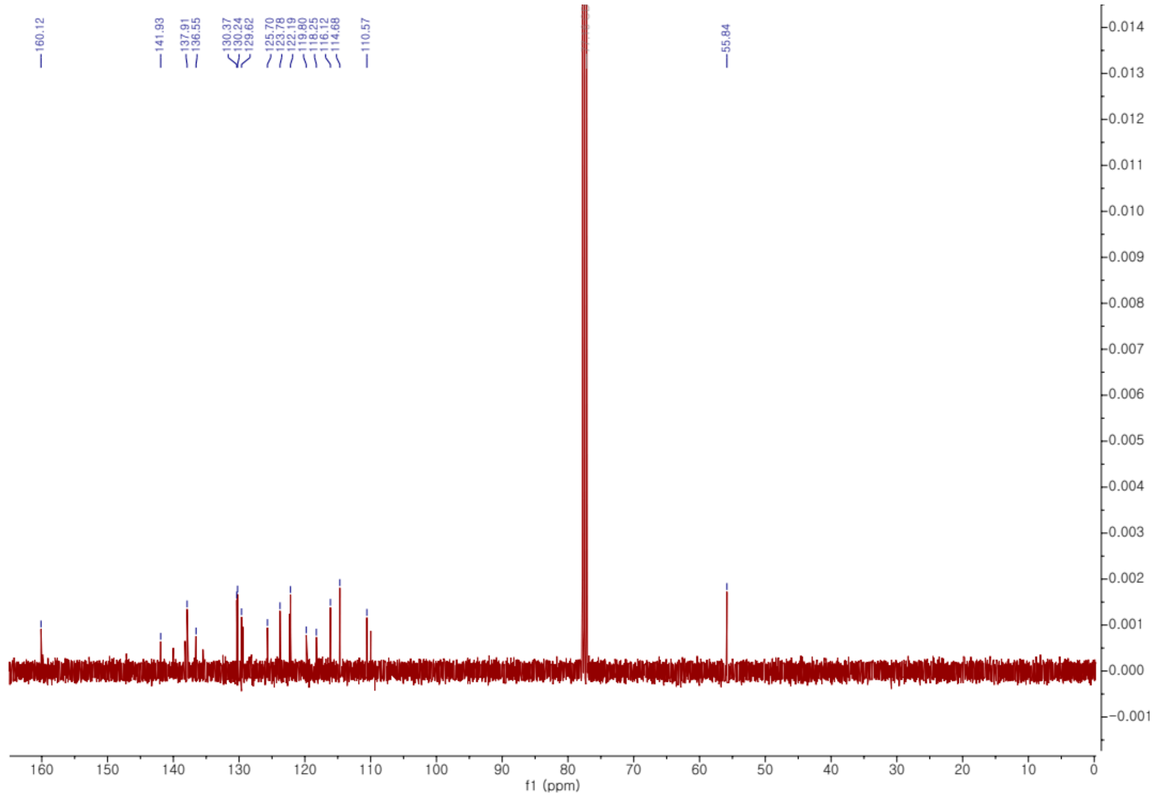

The mass data of OIC5

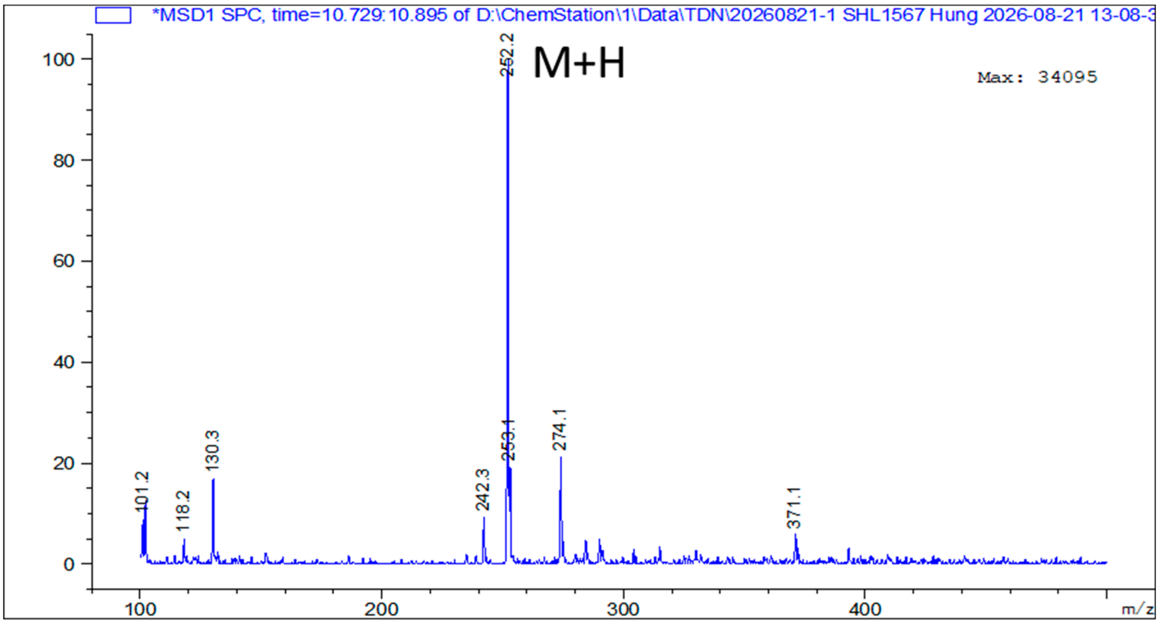

^{1}H-NMR($CDCl_3$) of OIC6

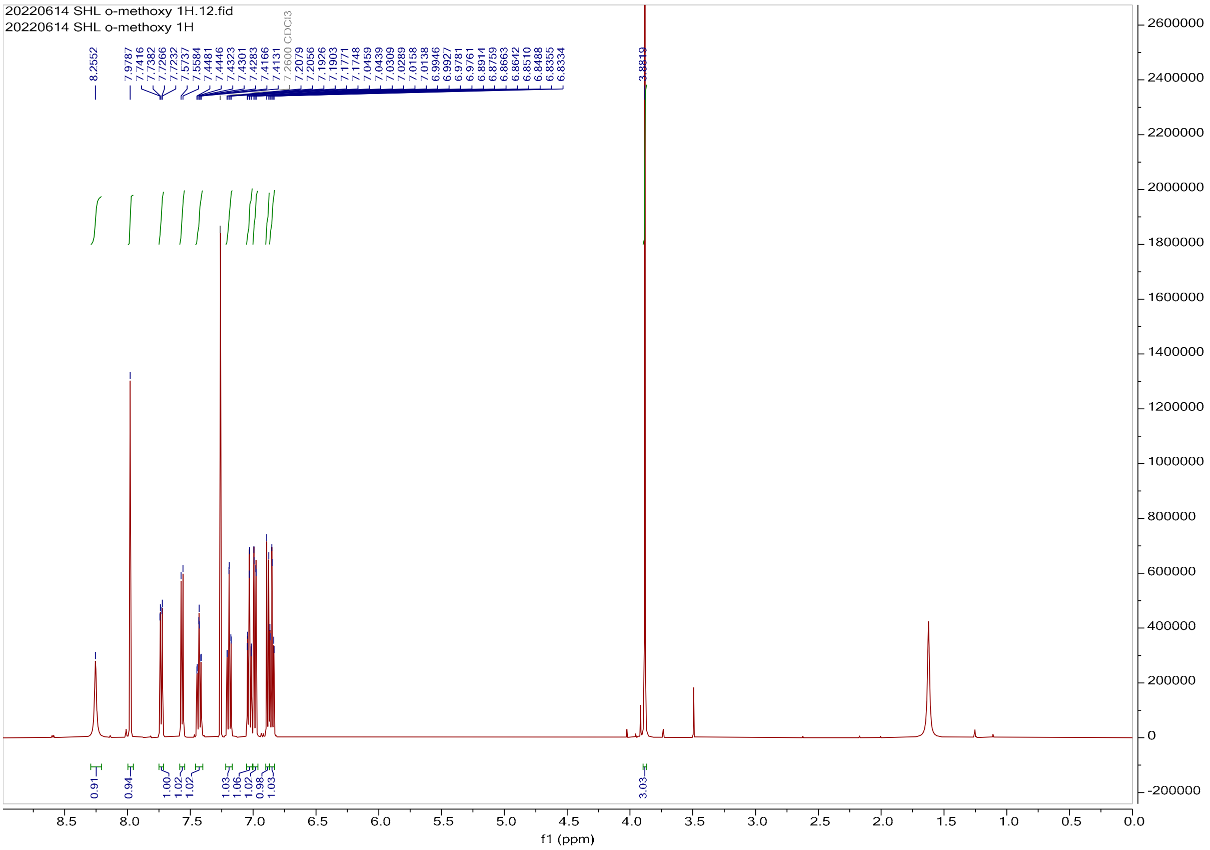

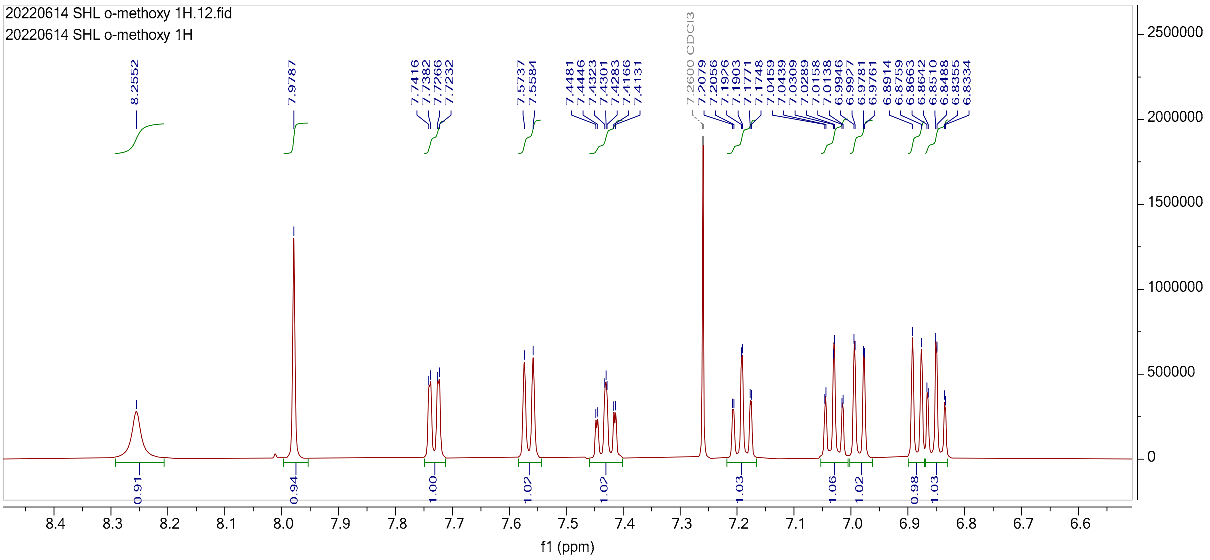

^{13}C-NMR(CDCl$_3$) of OIC6

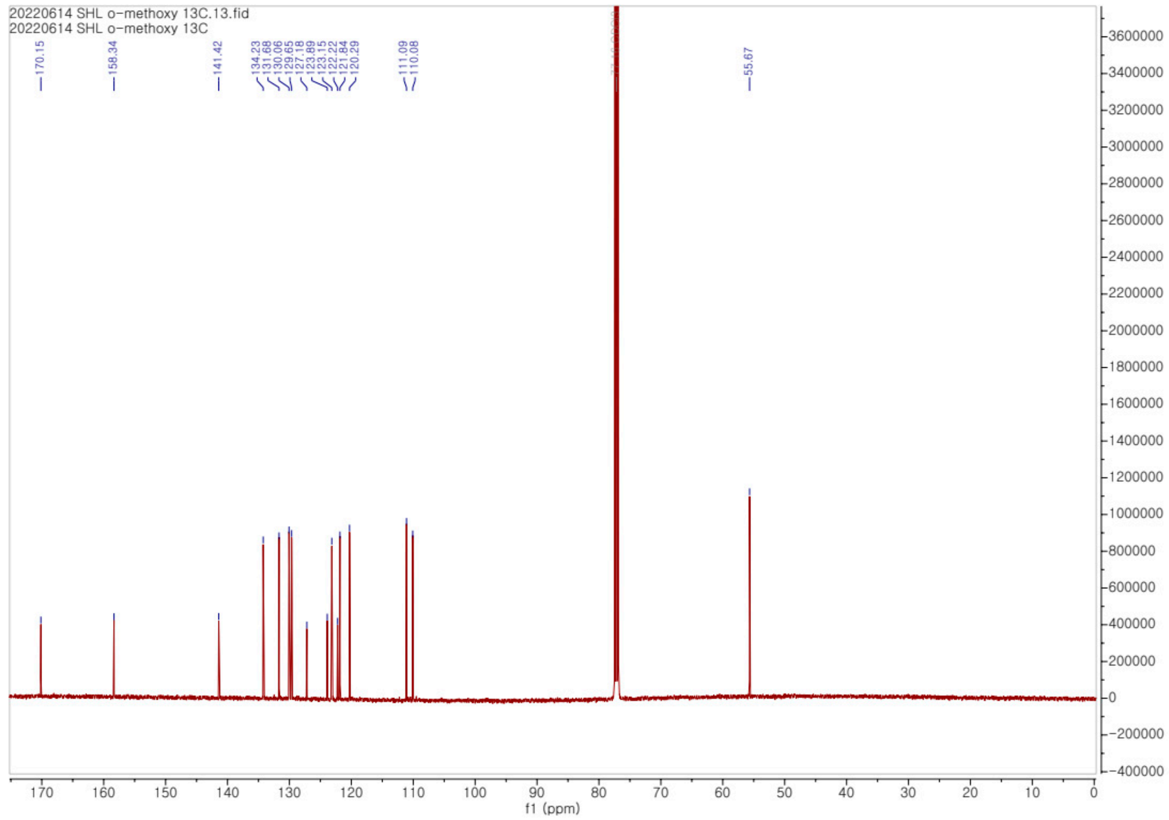

The mass data of OIC6

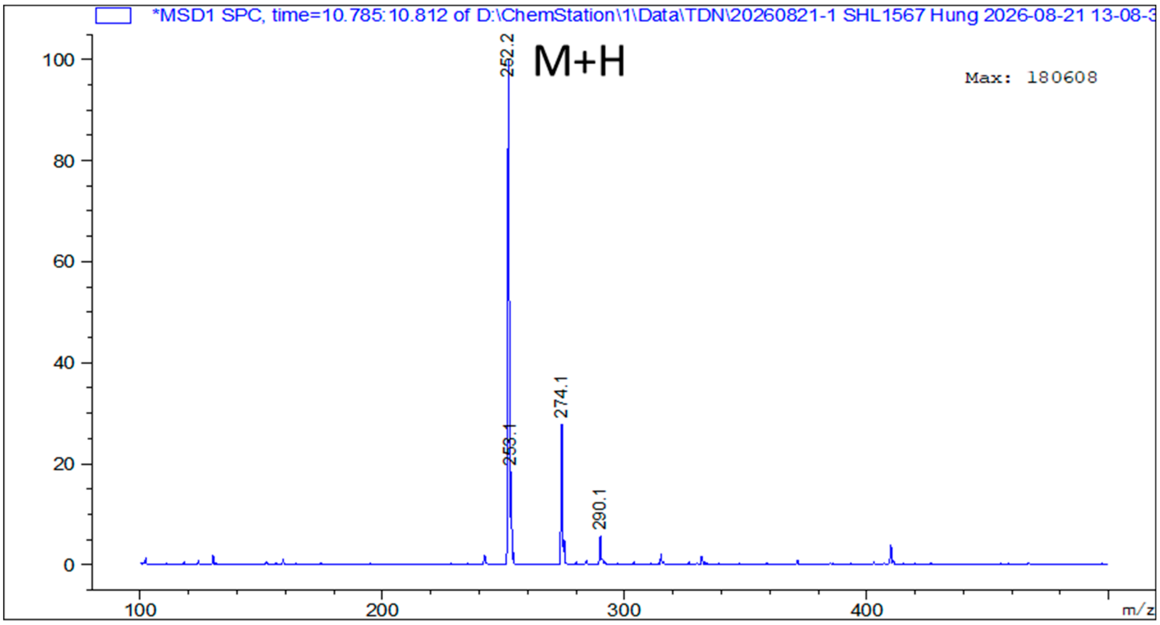

^{1}H-NMR (CDCl3) of OIC7

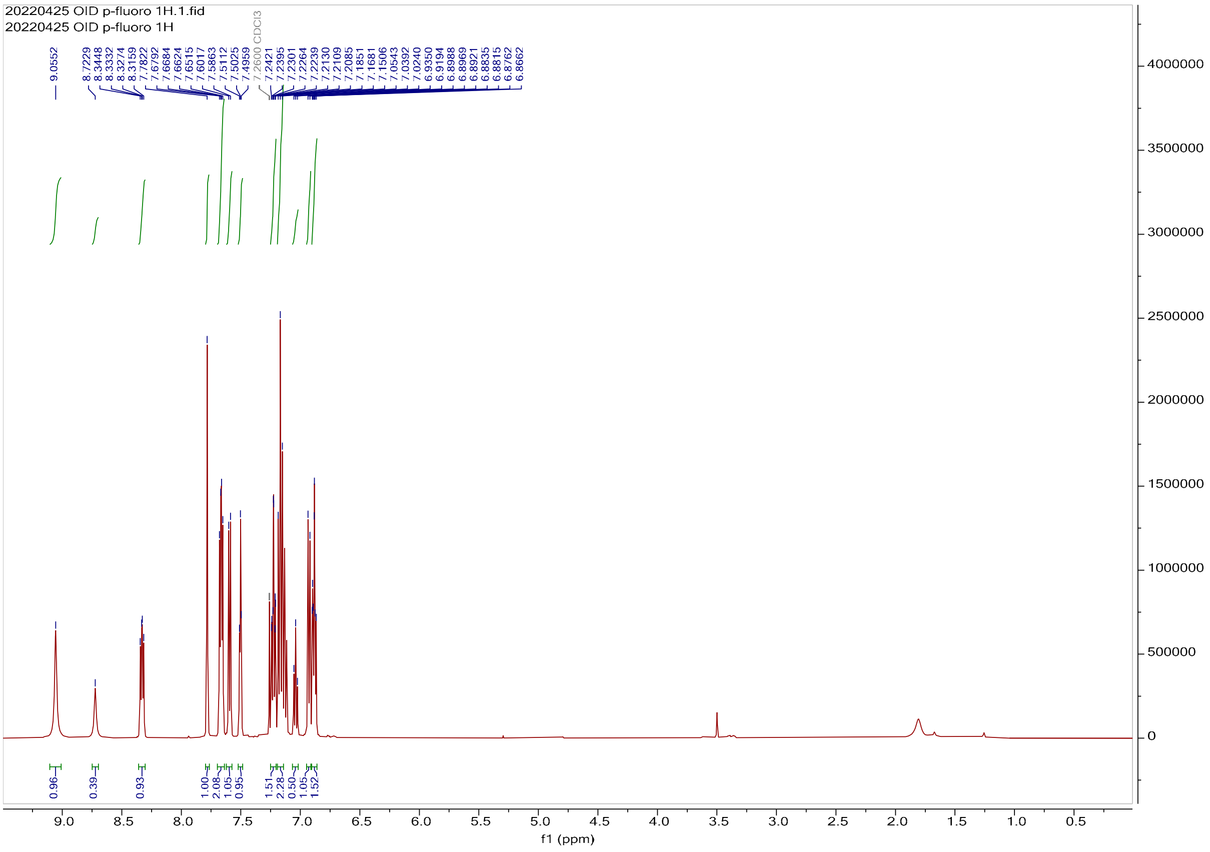

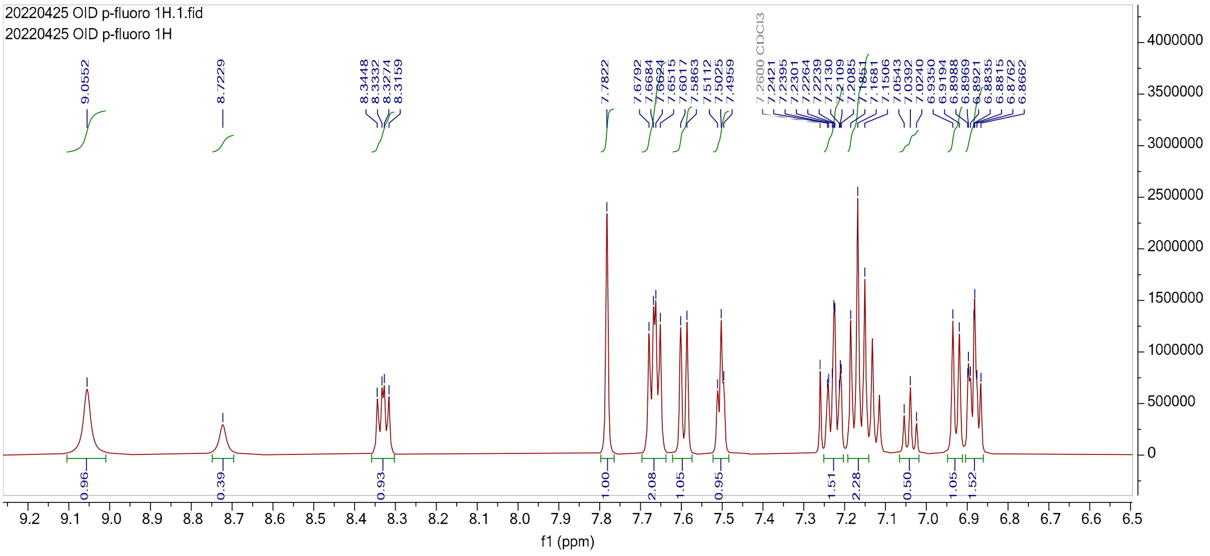

^{13}C-NMR ($CDCl_3$) of OIC7

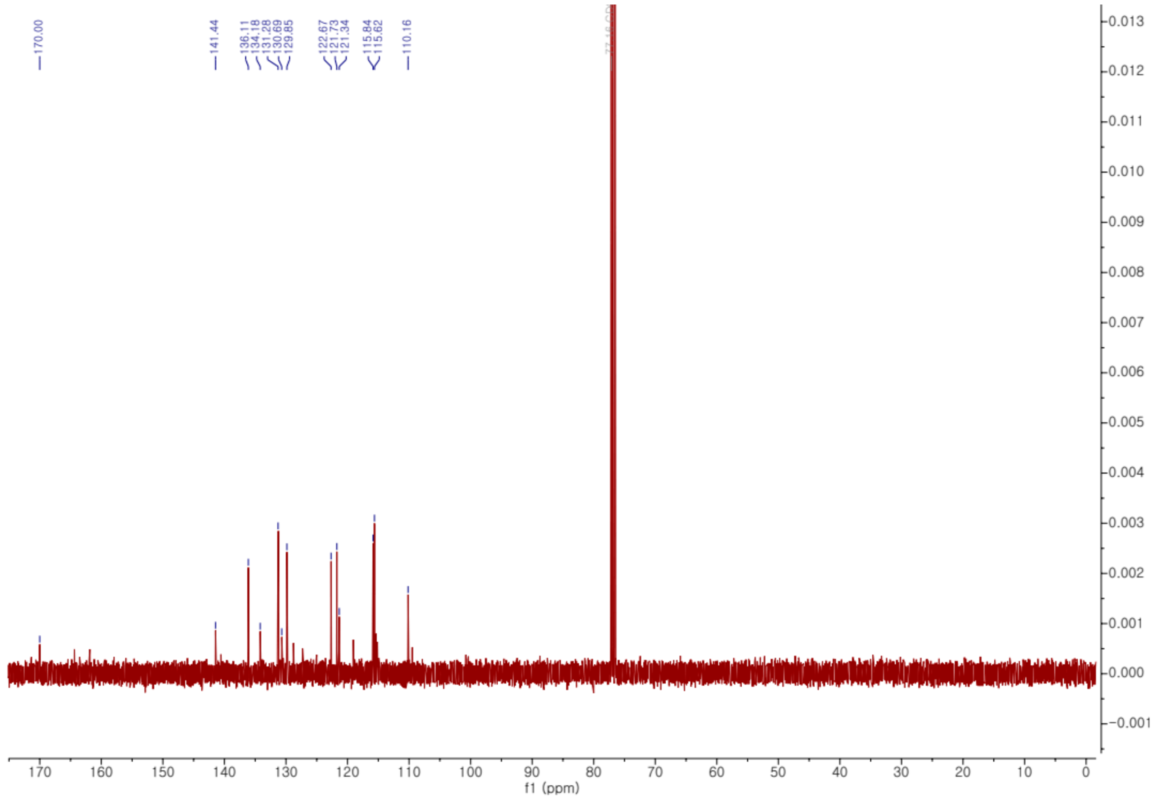

The mass data of OIC7

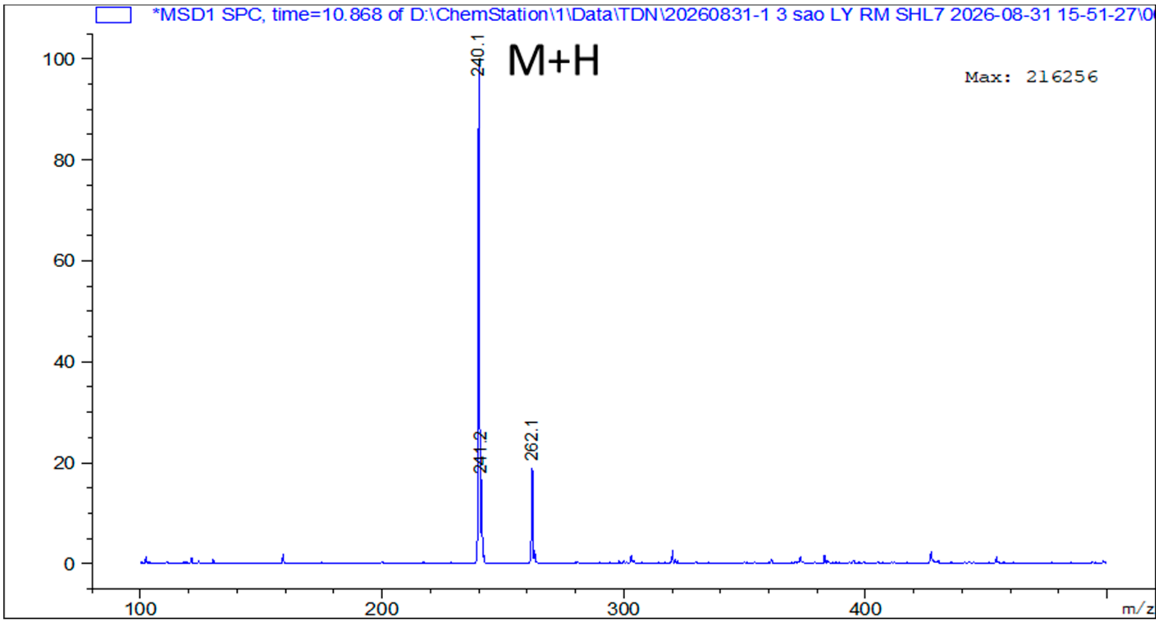

^{1}H-NMR(CDCl3) of OIC8

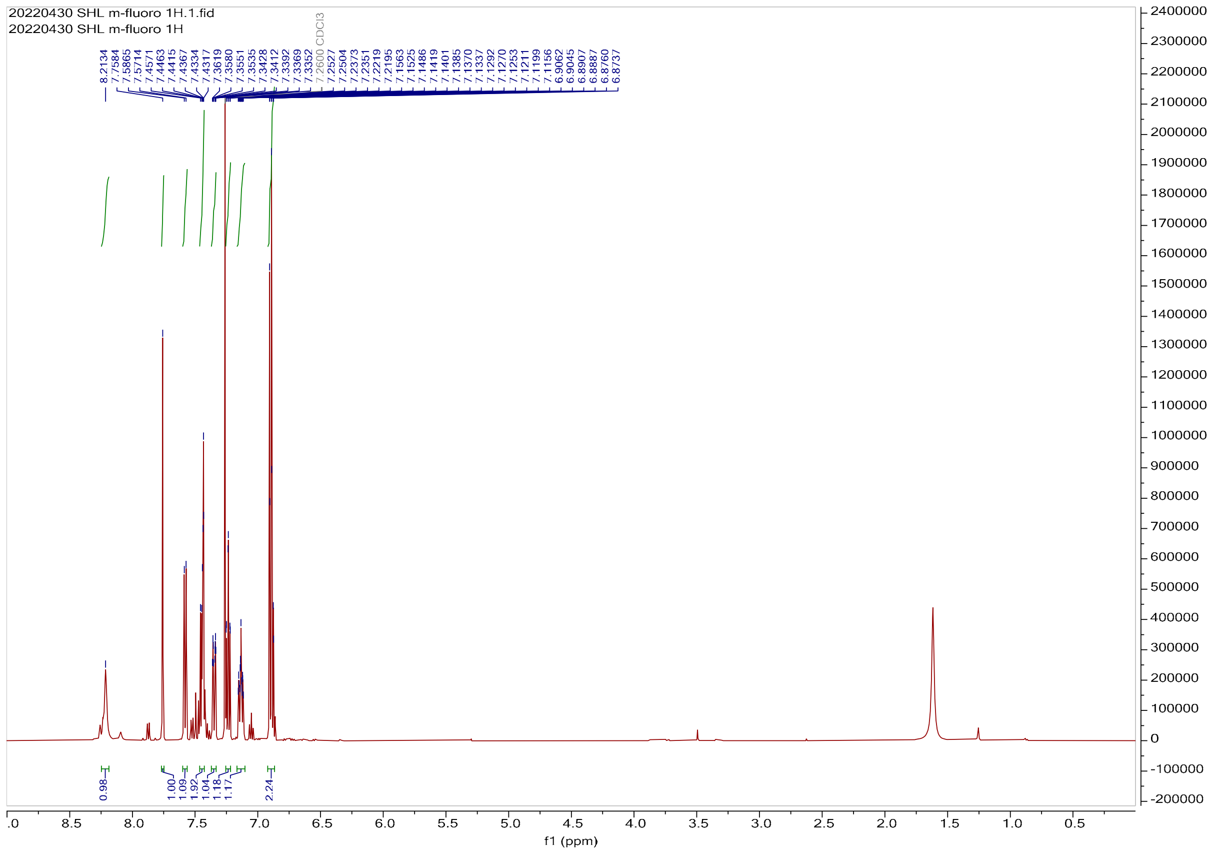
20220430 SHL m-fluoro 1H.1.fid
20220430 SHL m-fluoro 1H
CDCl3
f1 (ppm)

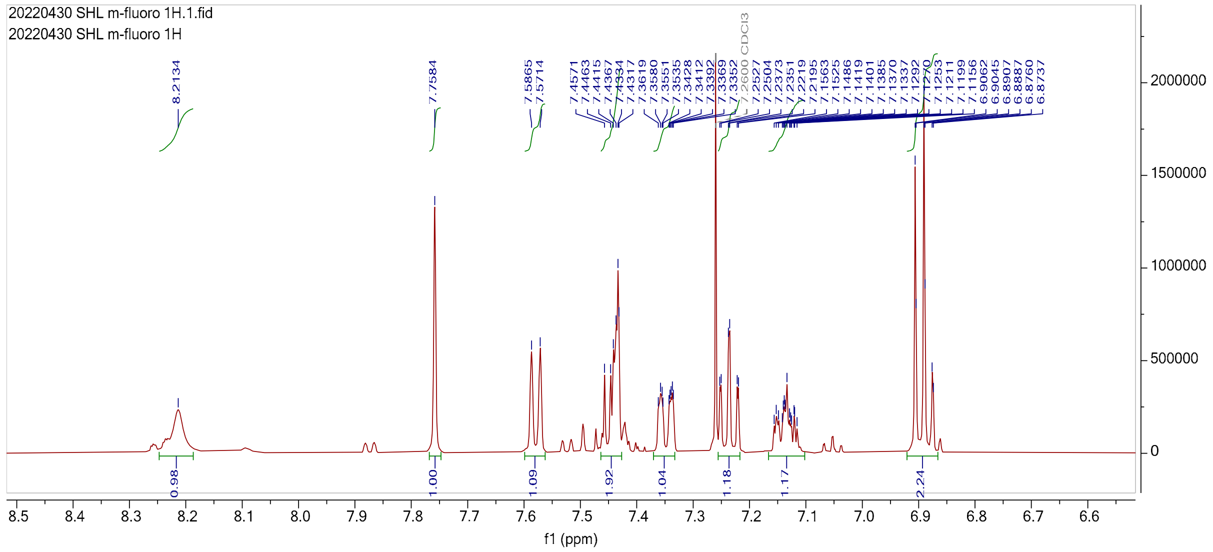
20220430 SHL m-fluoro 1H.1.fid
20220430 SHL m-fluoro 1H
8.2134
7.7584
7.5865
7.5714
CDCl3
f1 (ppm)

^{13}C-NMR($CDCl_3$) of OIC8

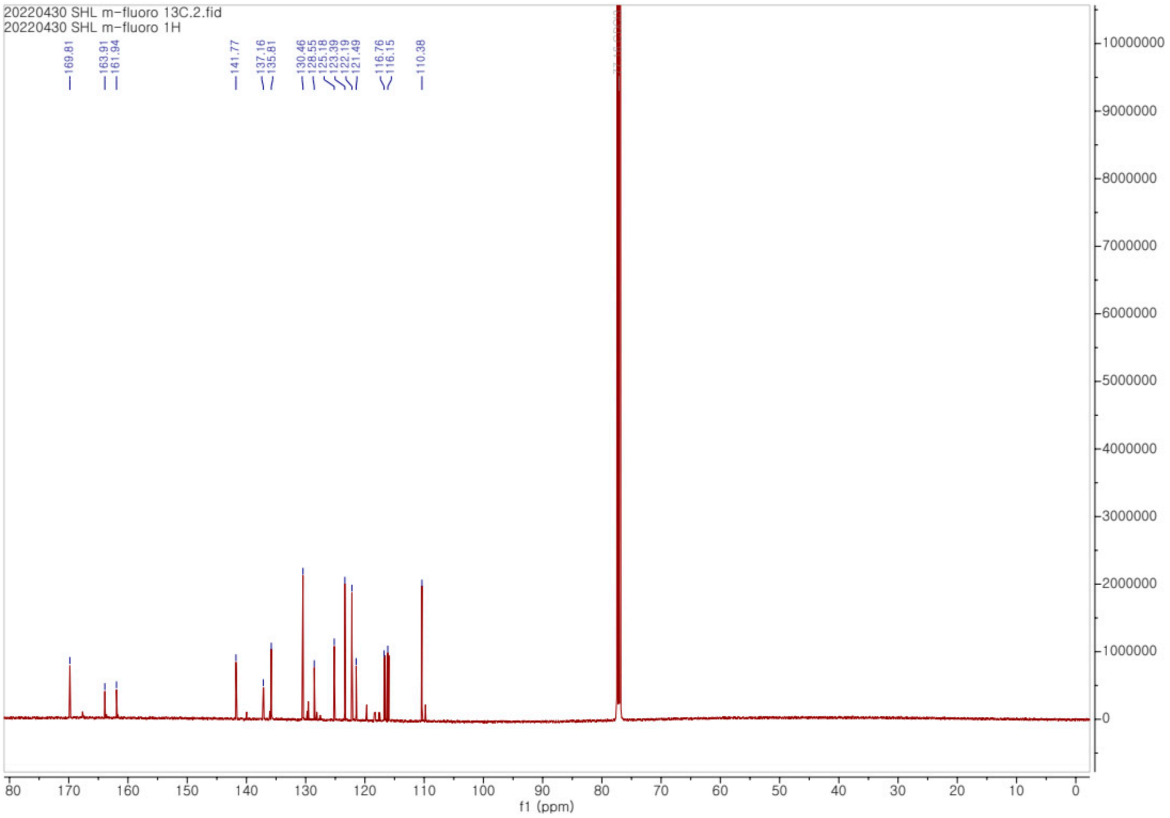

The mass data of OIC8

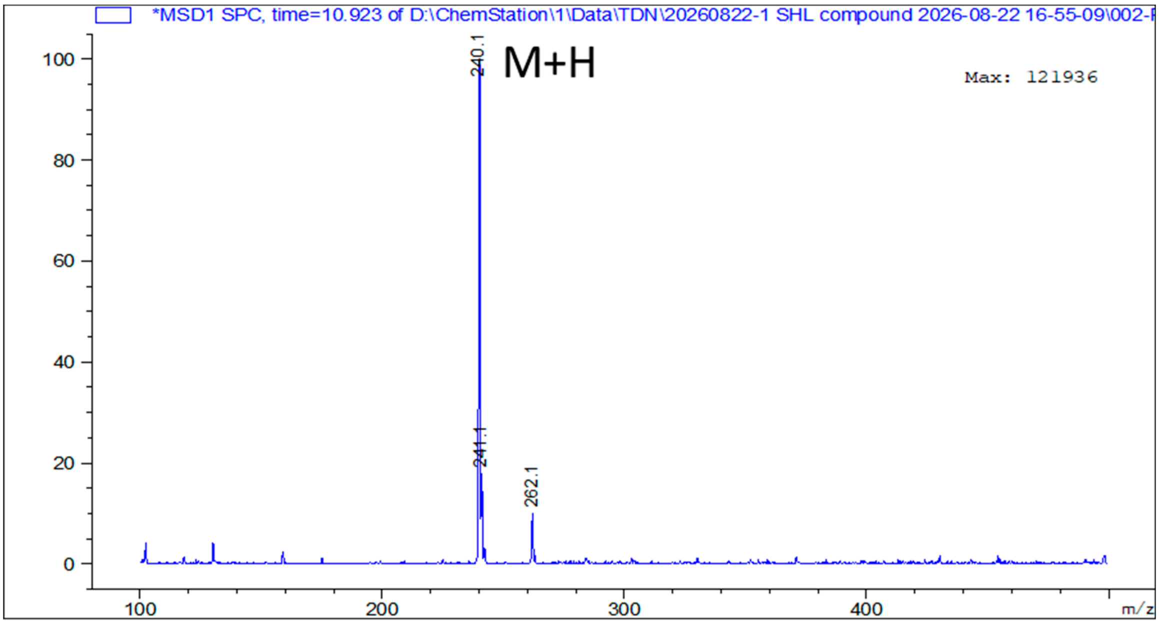

^{1}H-NMR($CDCl_3$) of OIC9

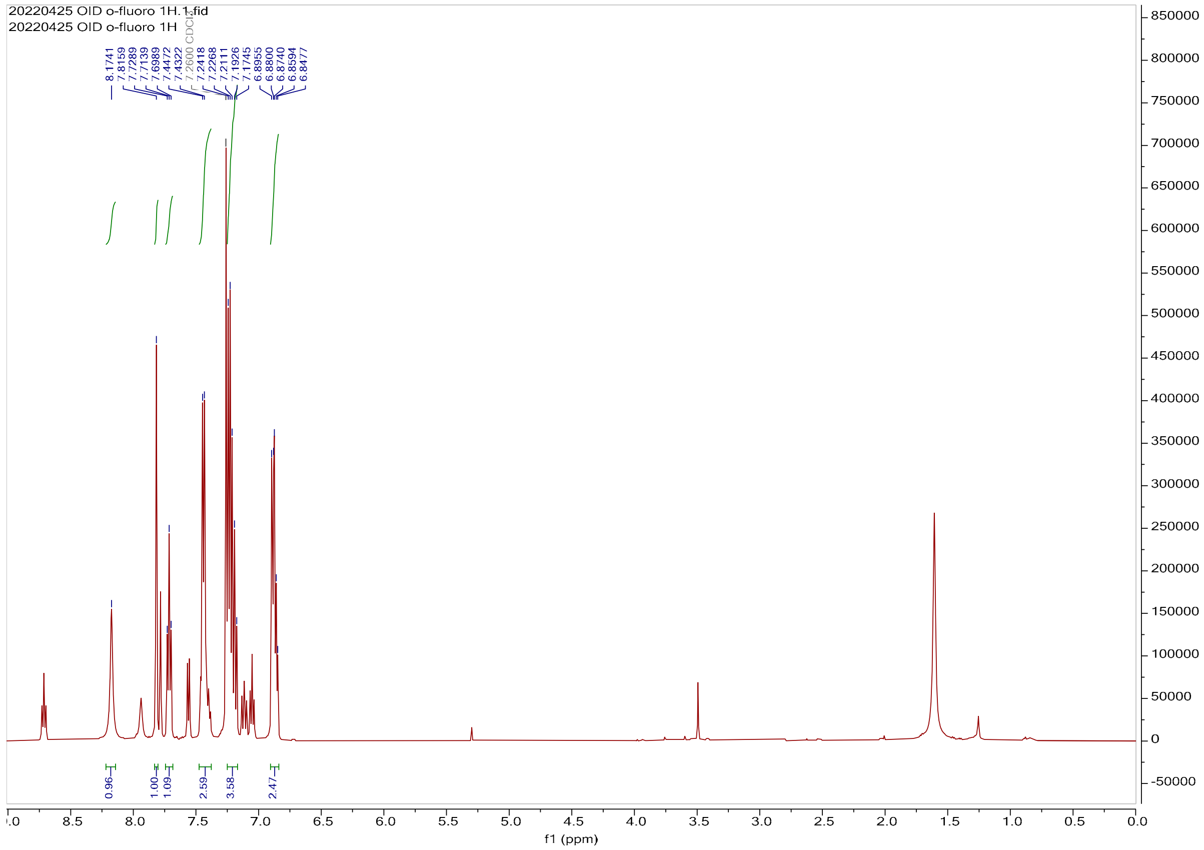

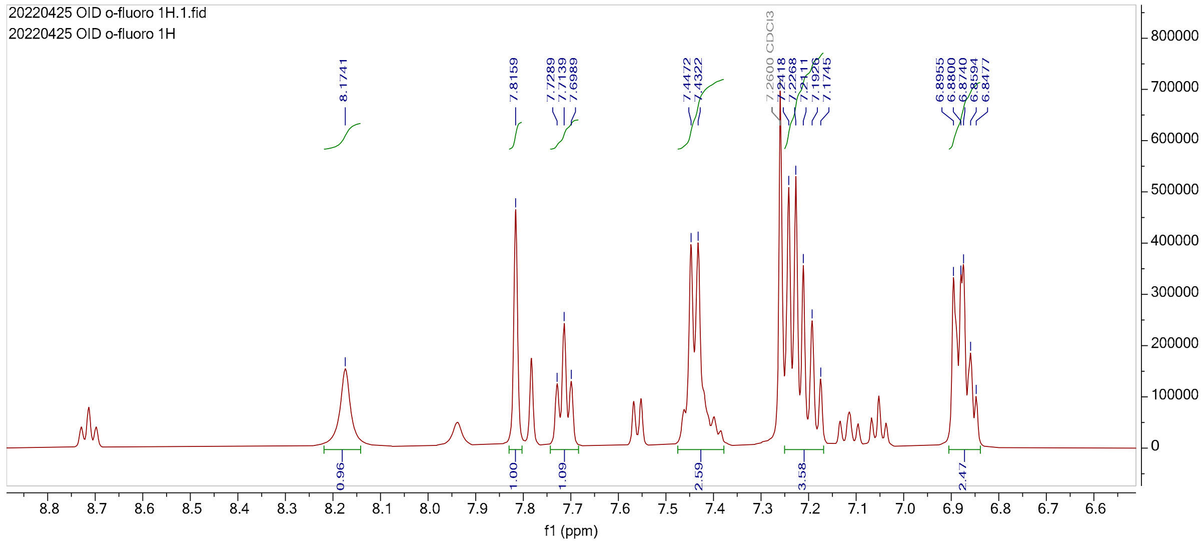

^{13}C-NMR($CDCl_3$) of OIC9

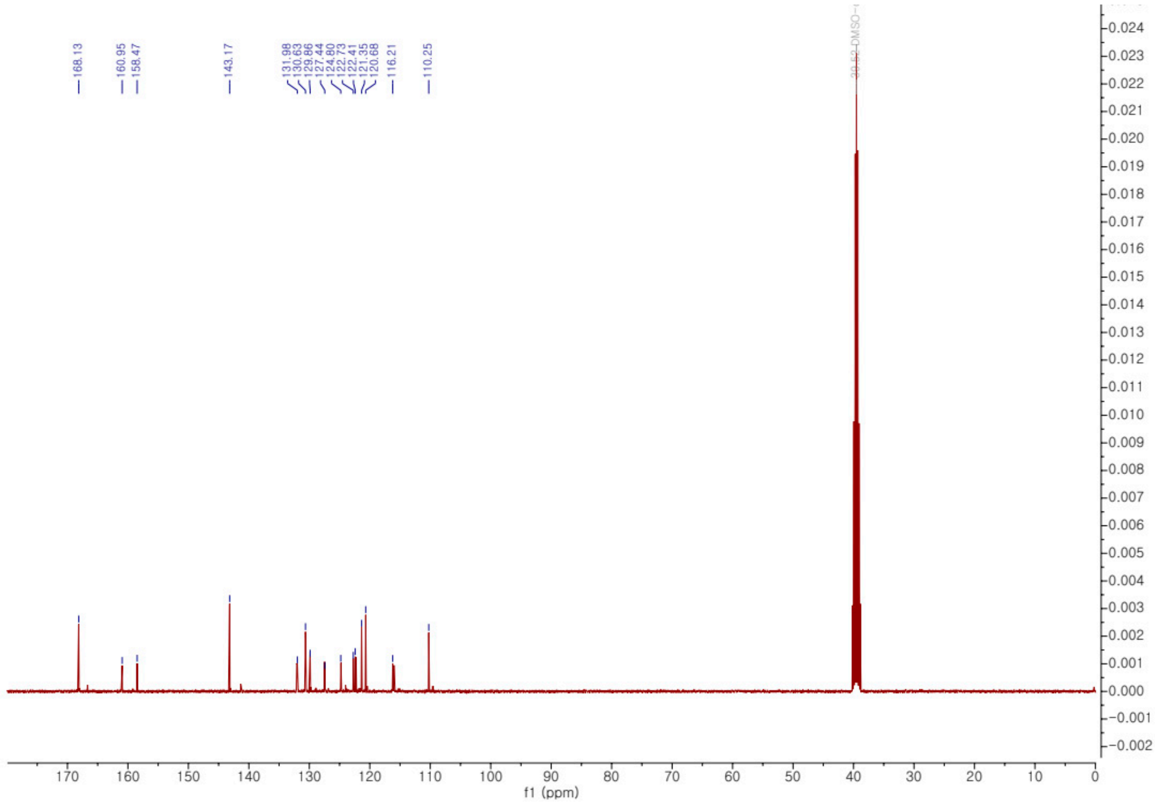

The mass data of OIC9

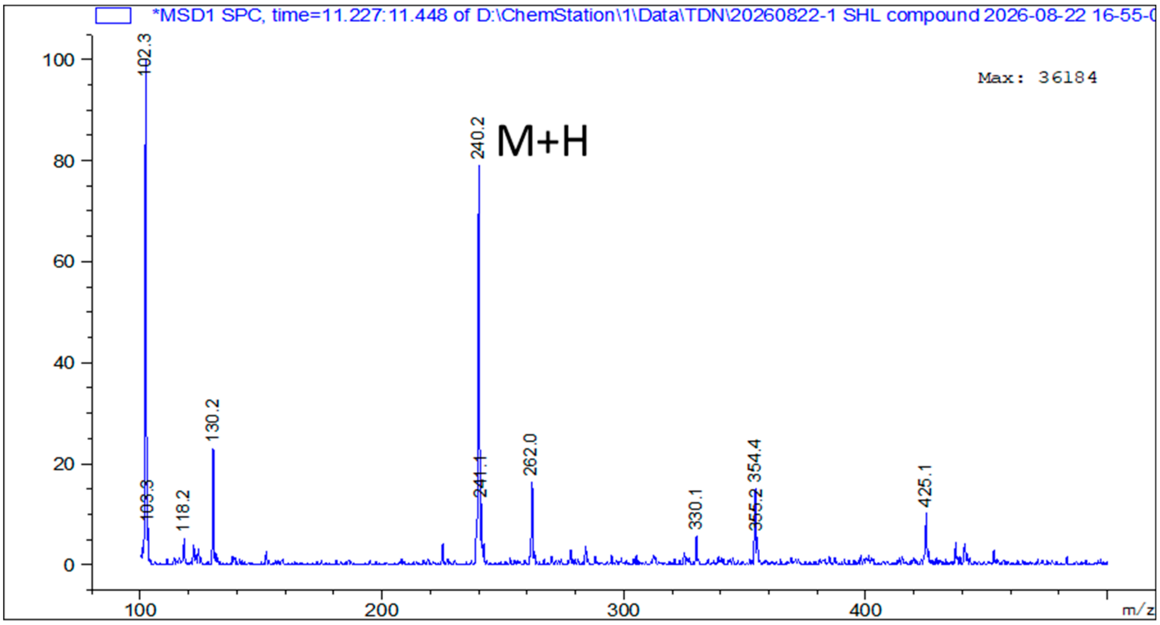

^{1}H-NMR(DMSO-d_6) of OIC10

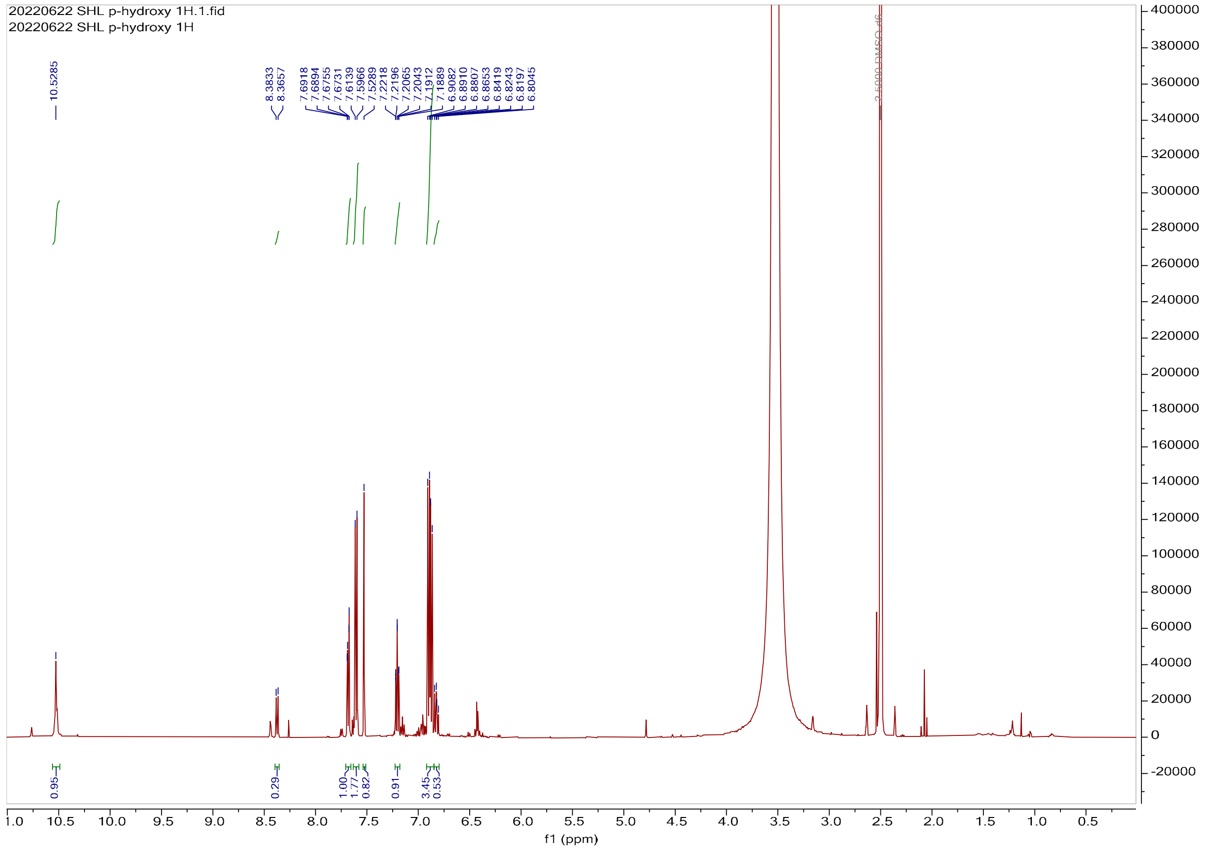

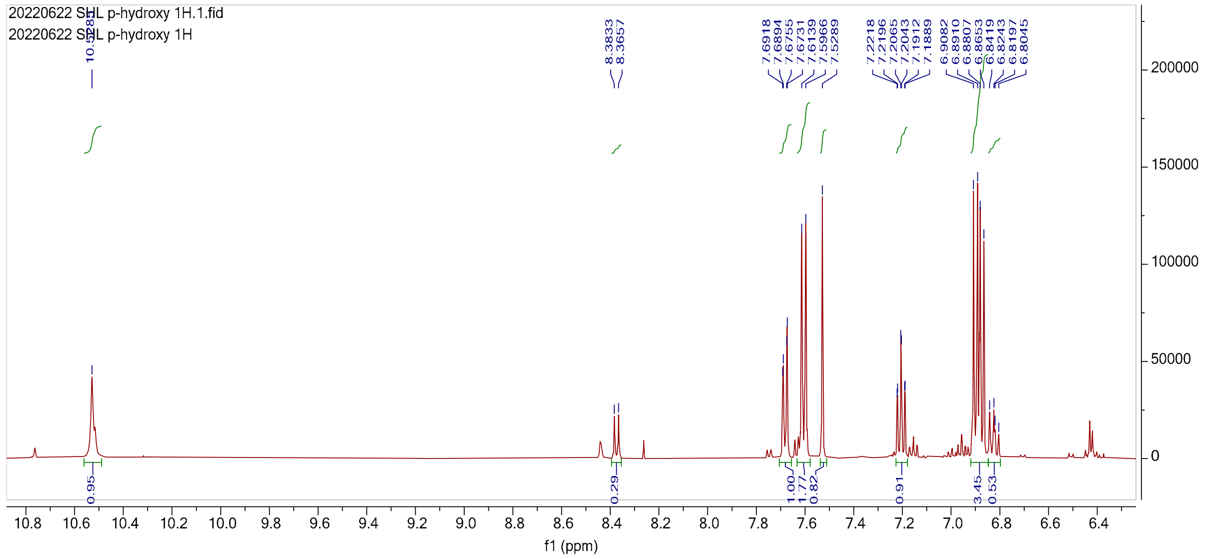

^{13}C-NMR(DMSO-d_6) of OIC10

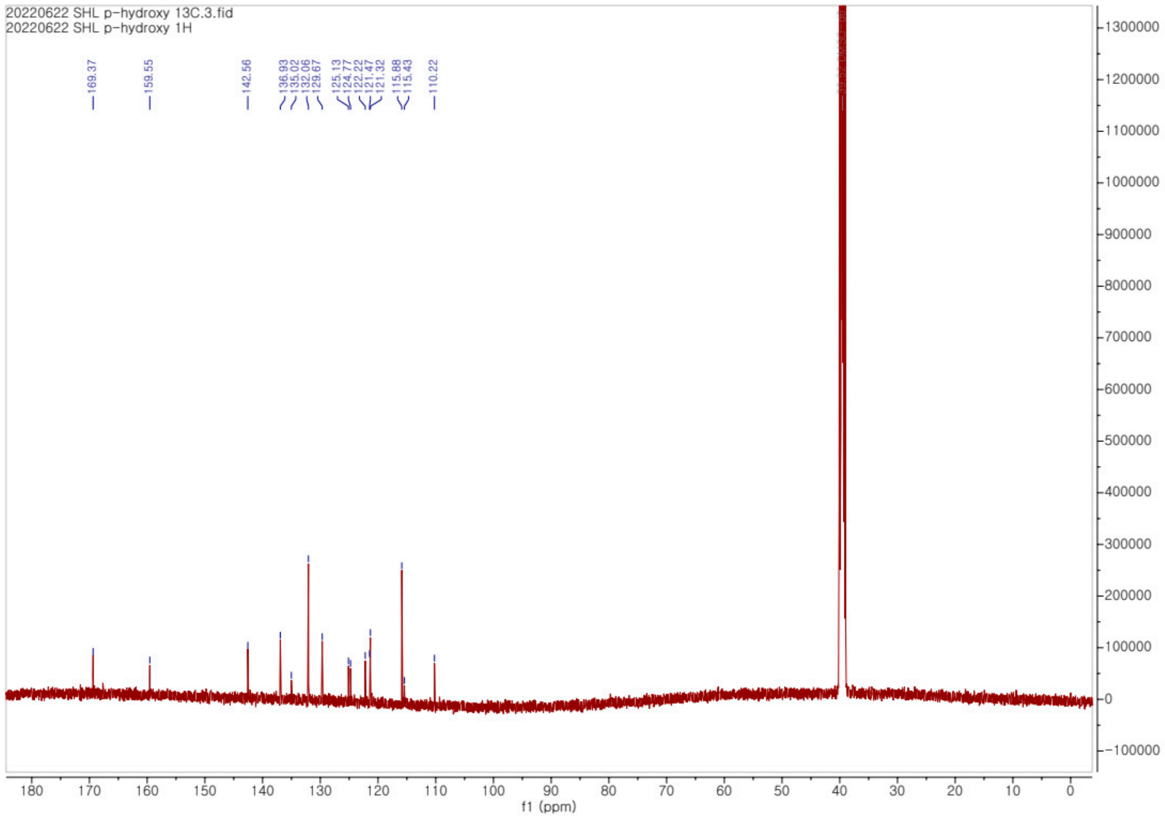

The mass data of OIC10

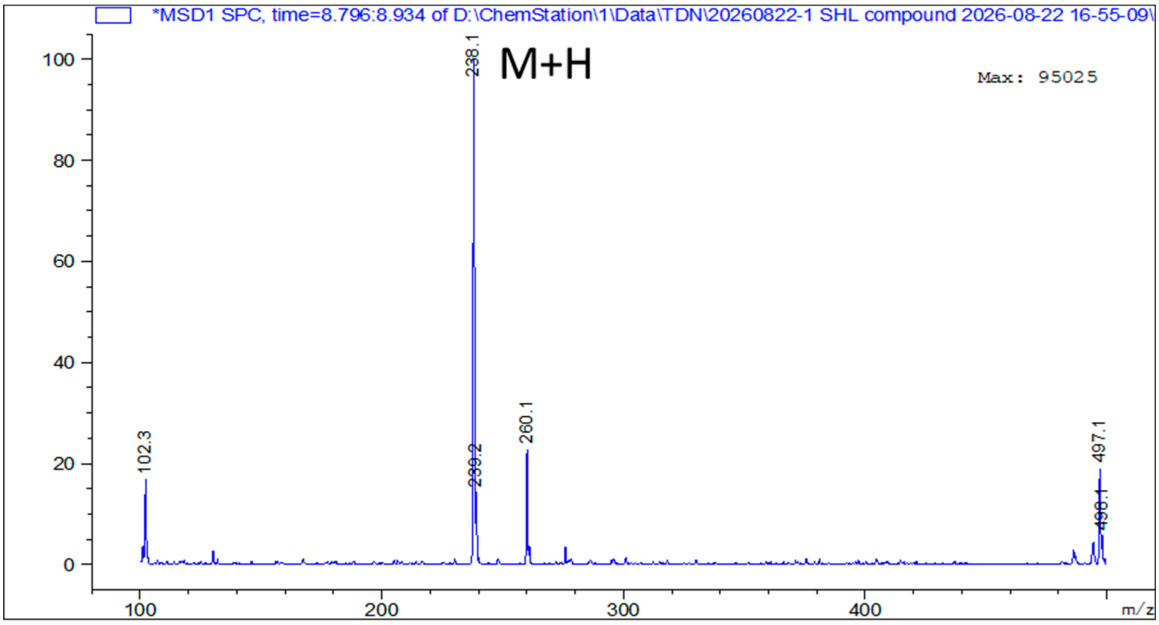

^{1}H-NMR(DMSO-d_6) of OIC11

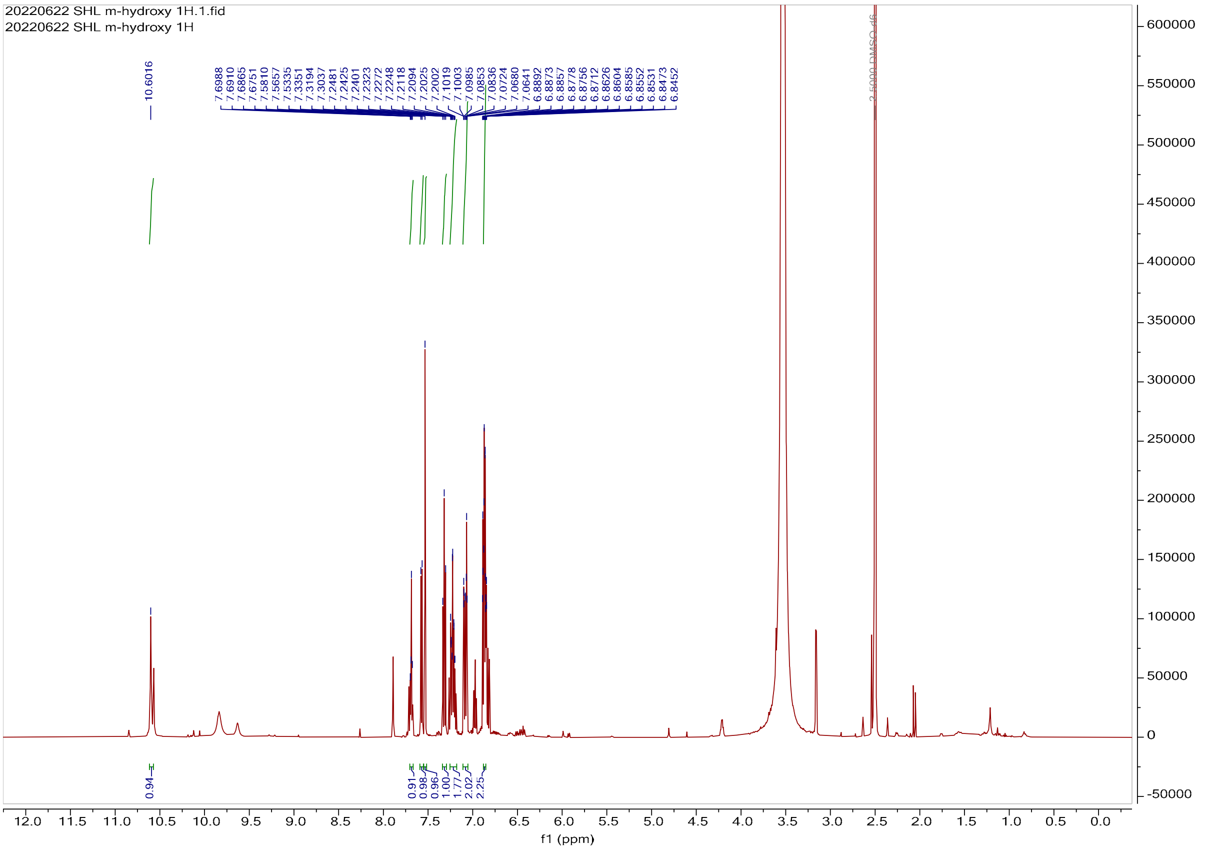

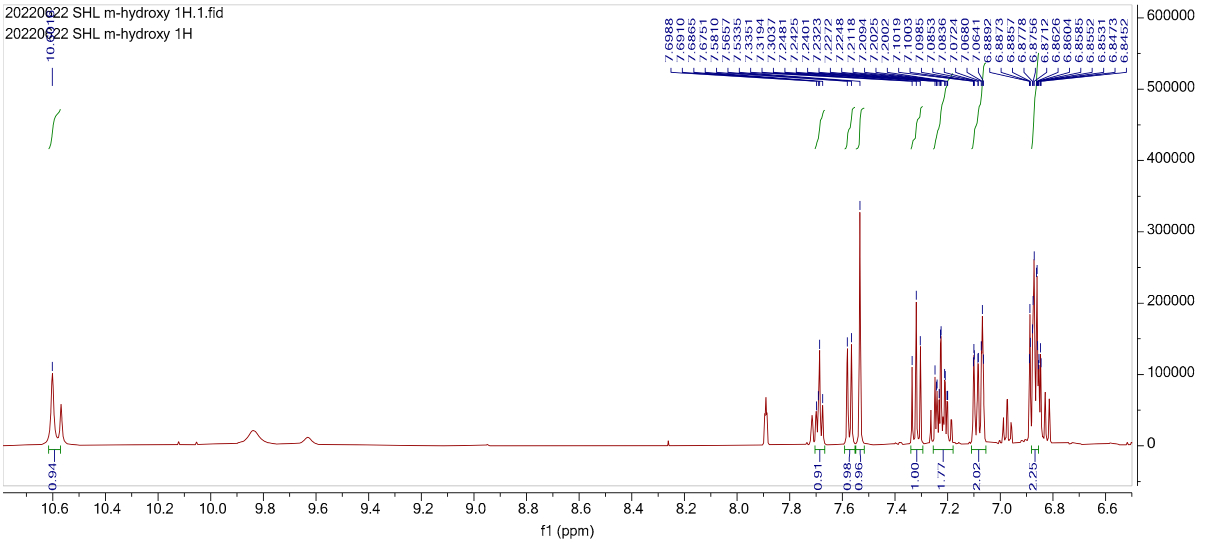

^{13}C-NMR(DMSO-d6) of OIC11

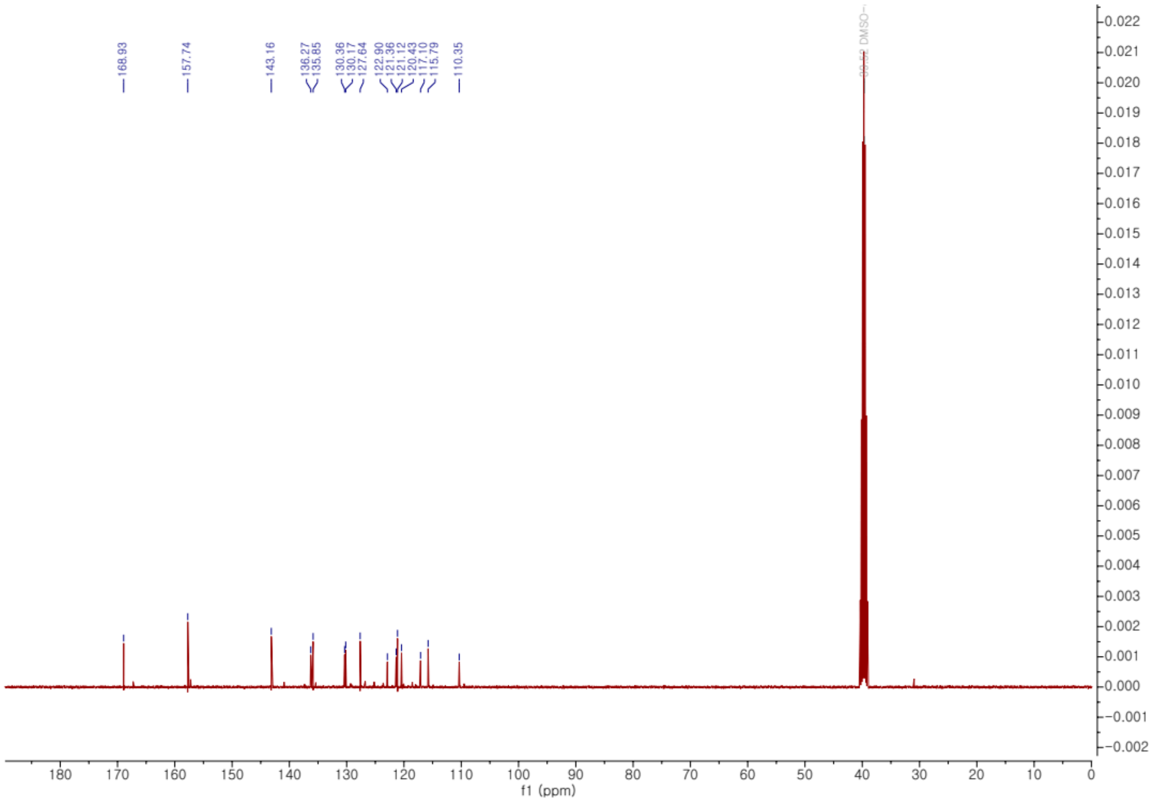

The mass data of OIC11

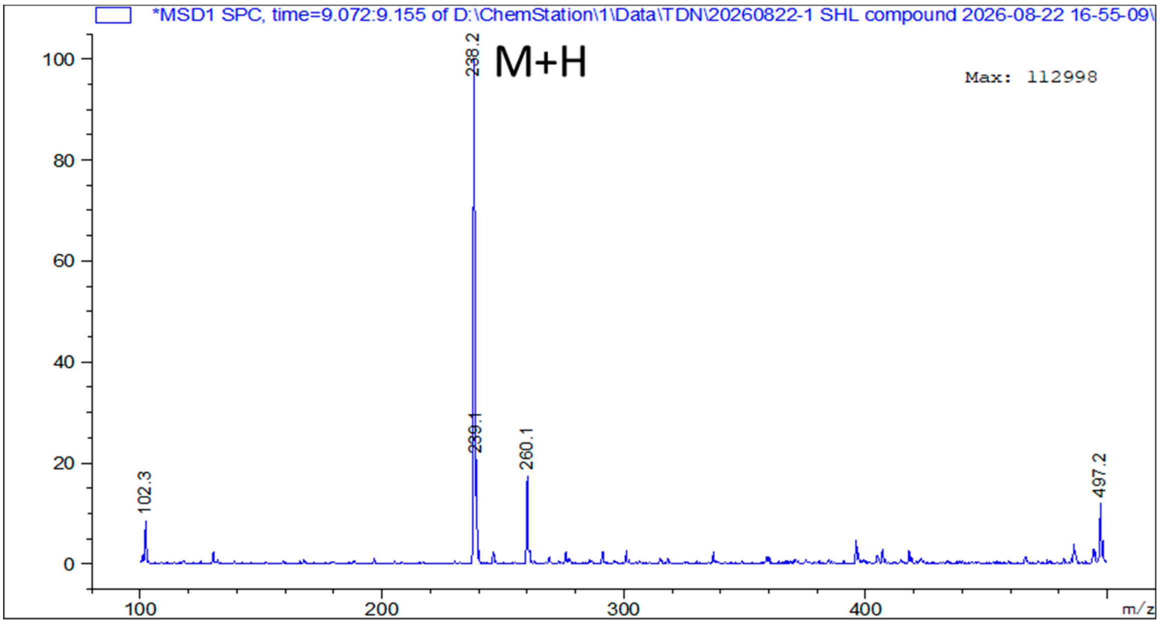

^{1}H-NMR(DMSO-d_6) of OIC12

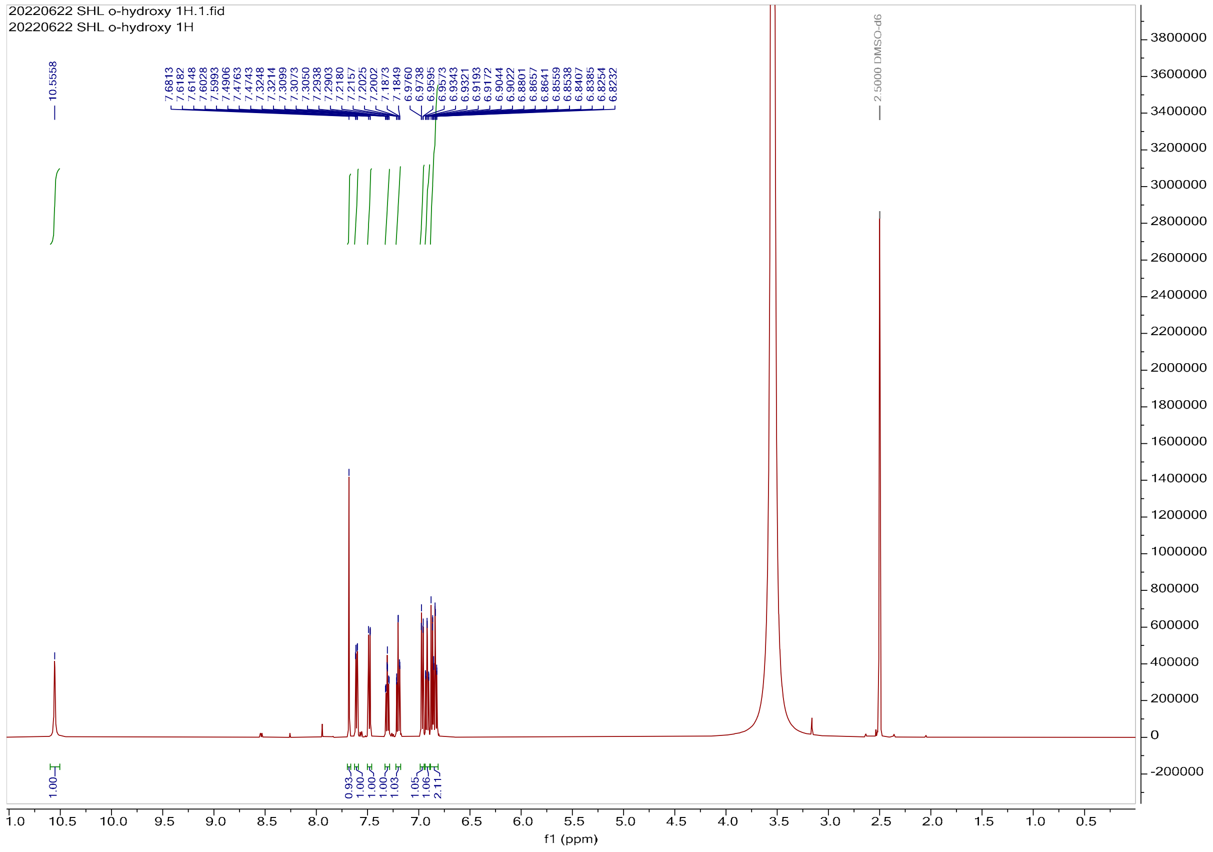

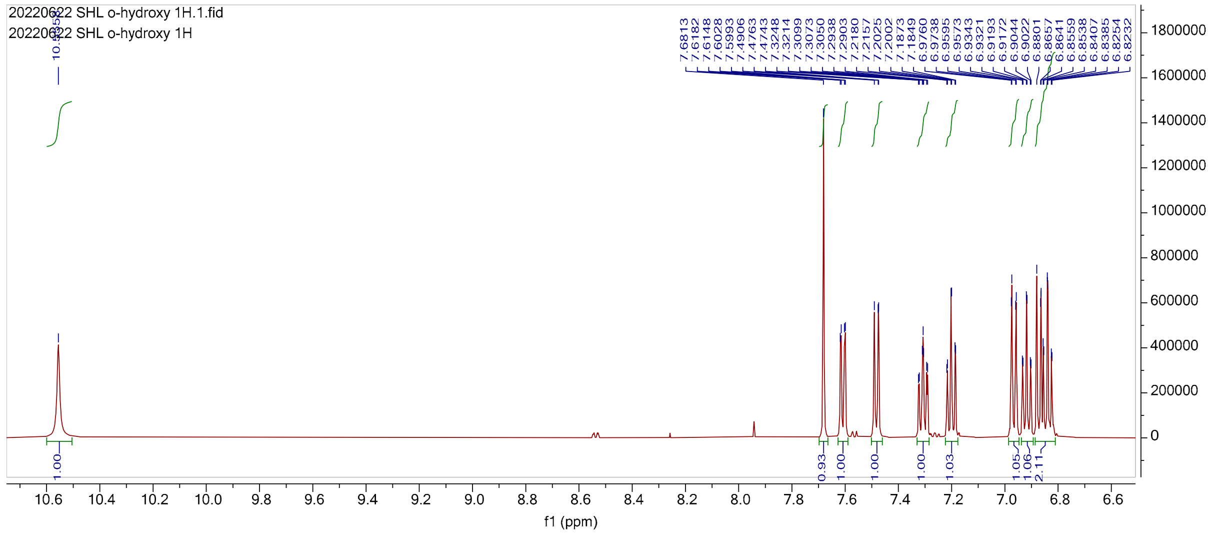

^{13}C-NMR(DMSO-d$_6$) of OIC12

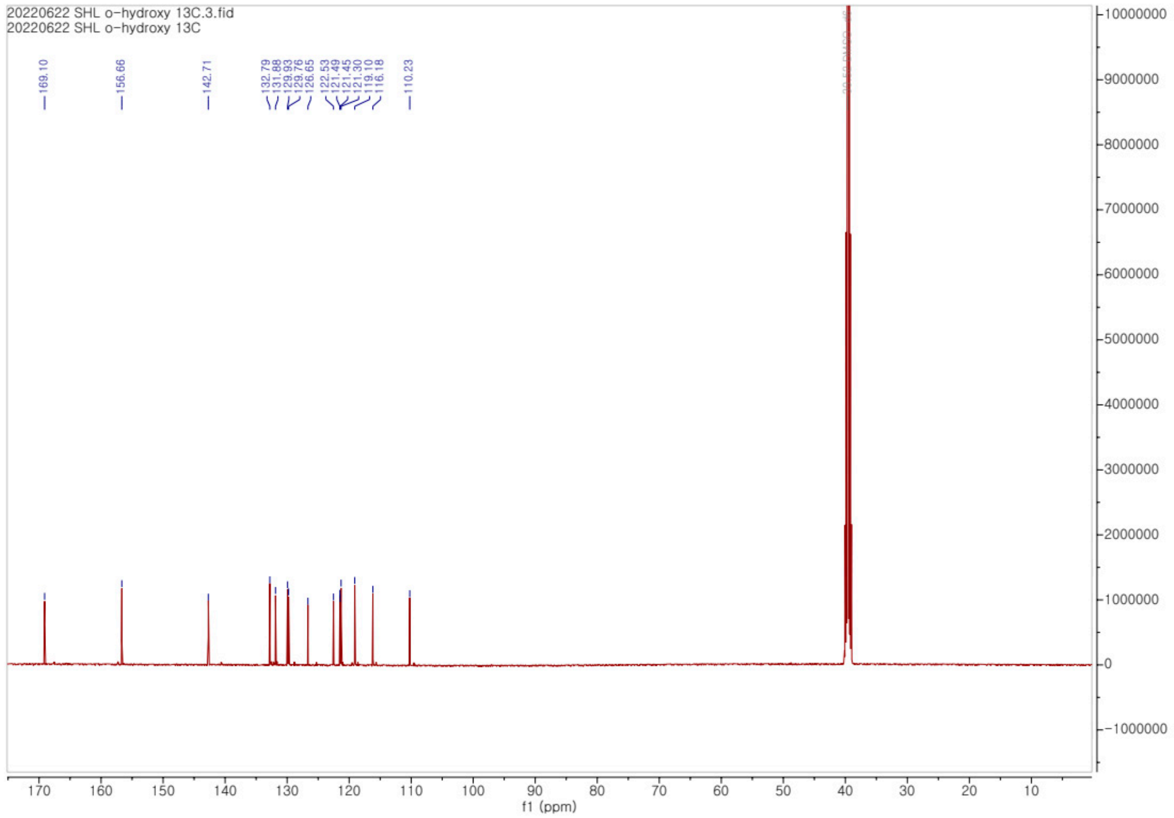

The mass data of OIC12

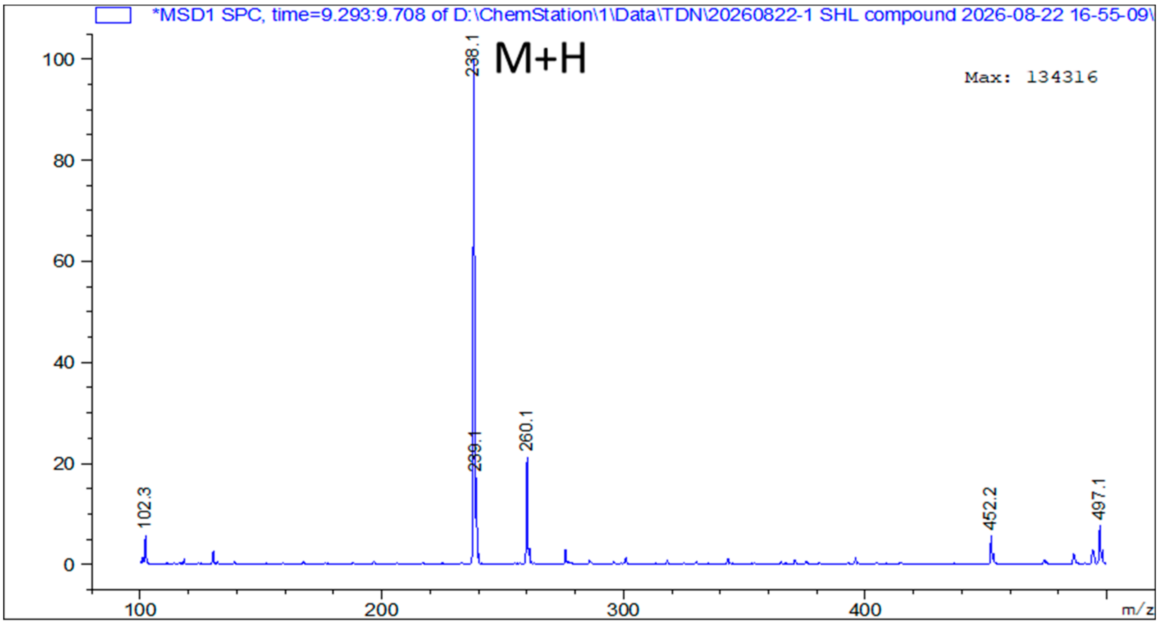

^{1}H-NMR($CDCl_3$) of OIC13

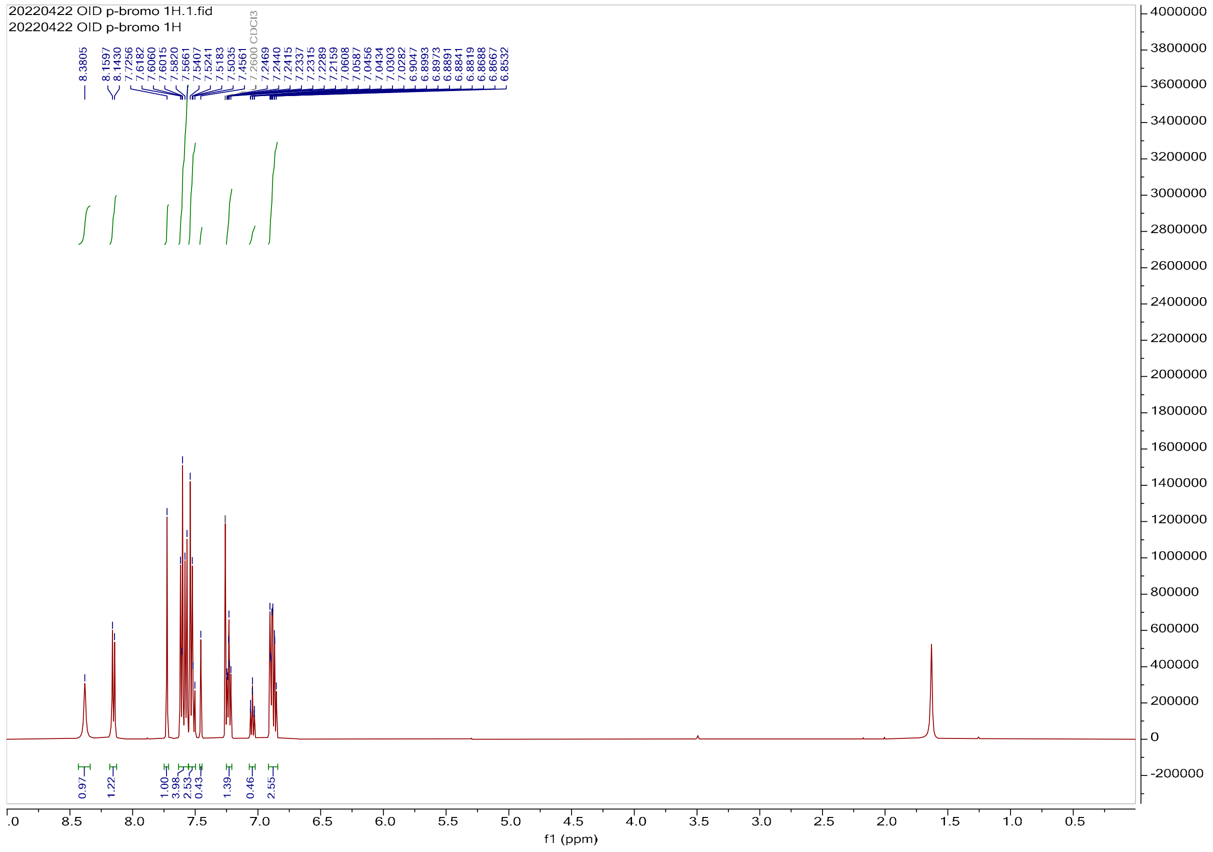

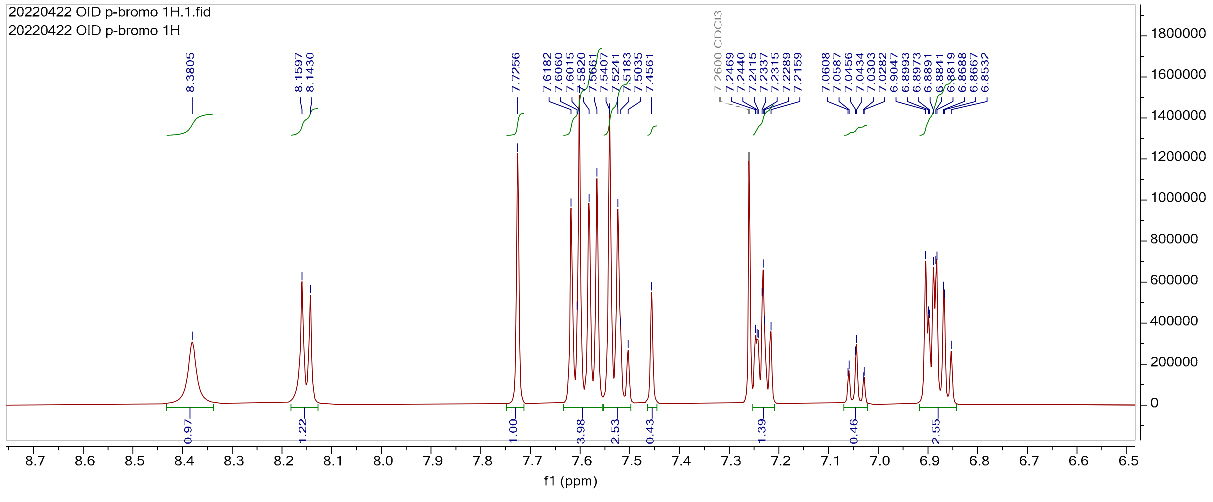

^{13}C-NMR($CDCl_3$) of OIC13

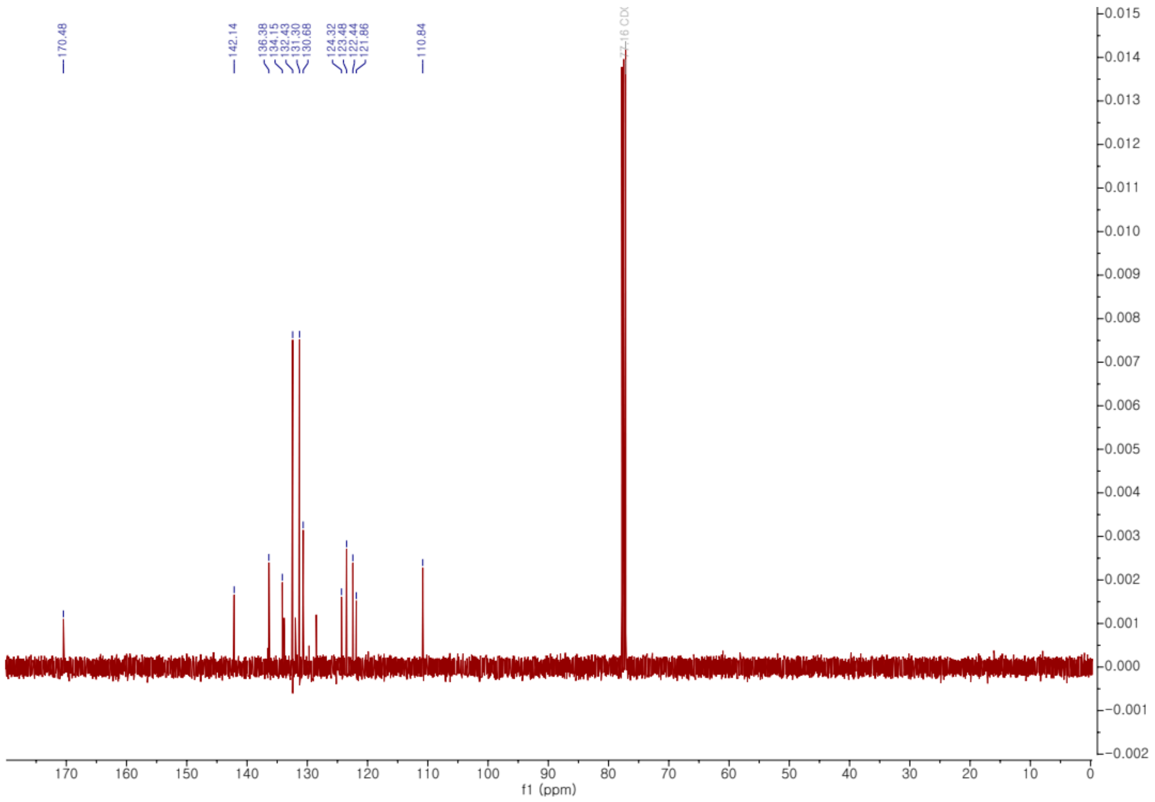

The mass data of OIC13

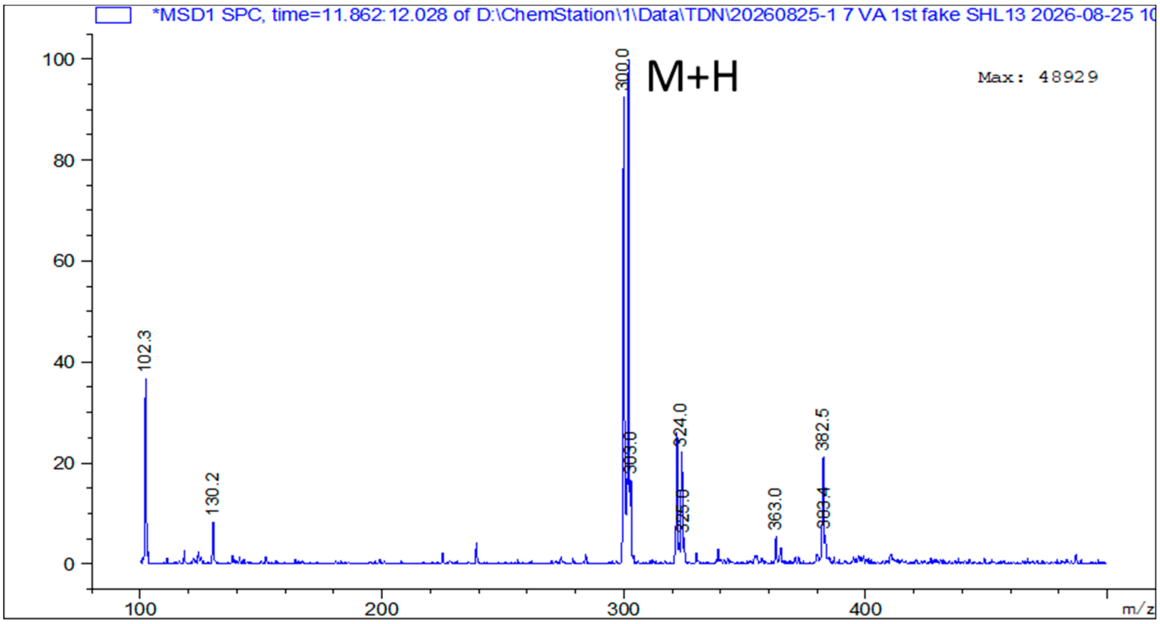

^{1}H-NMR($CDCl_3$) of OIC14

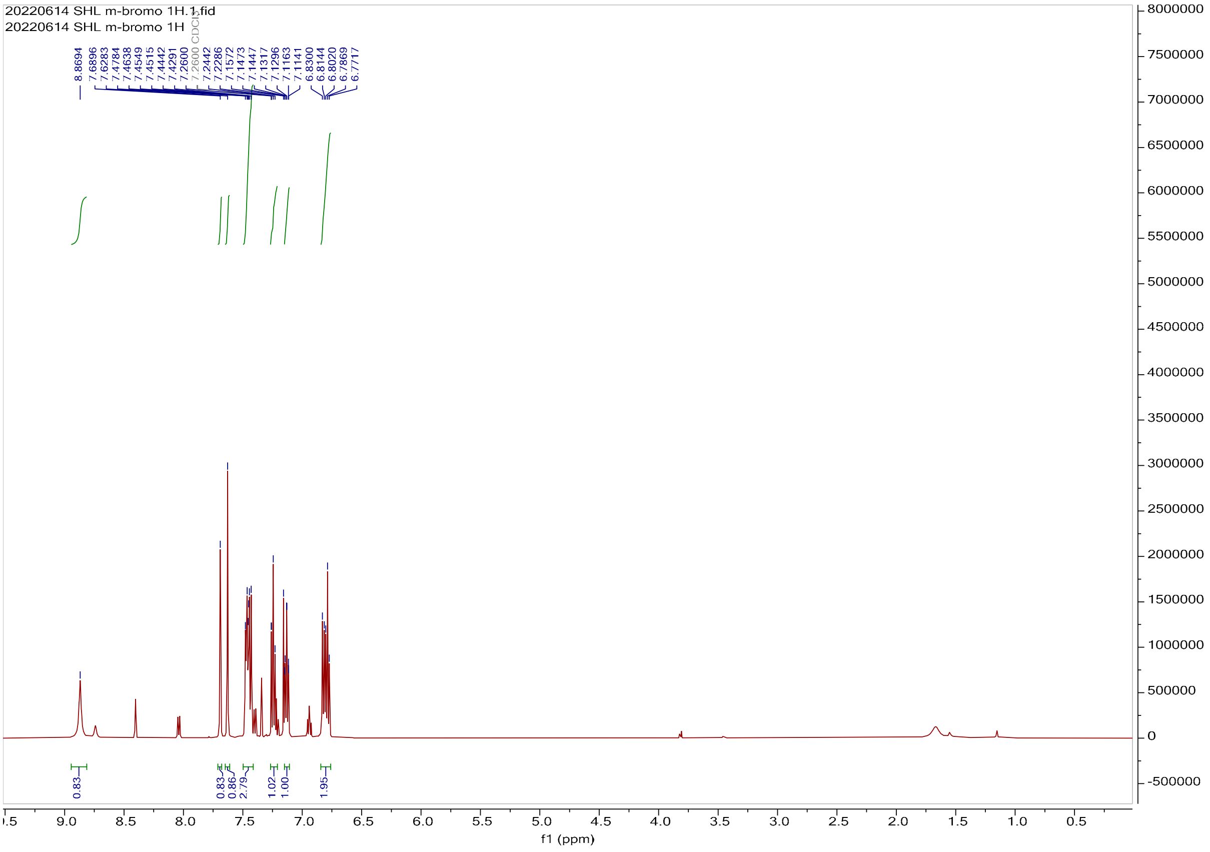

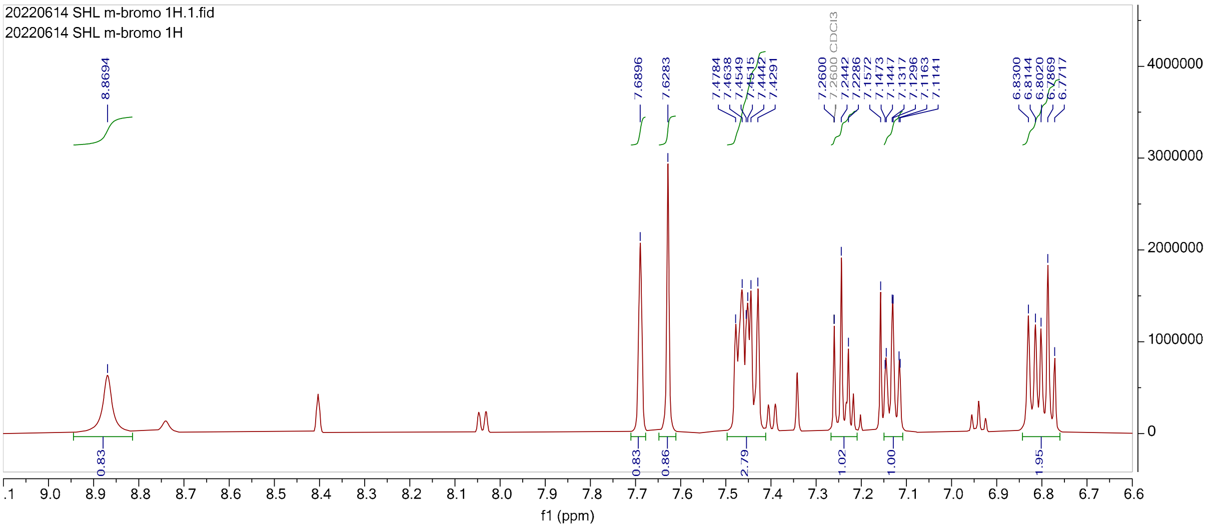

^{13}C-NMR($CDCl_3$) of OIC14

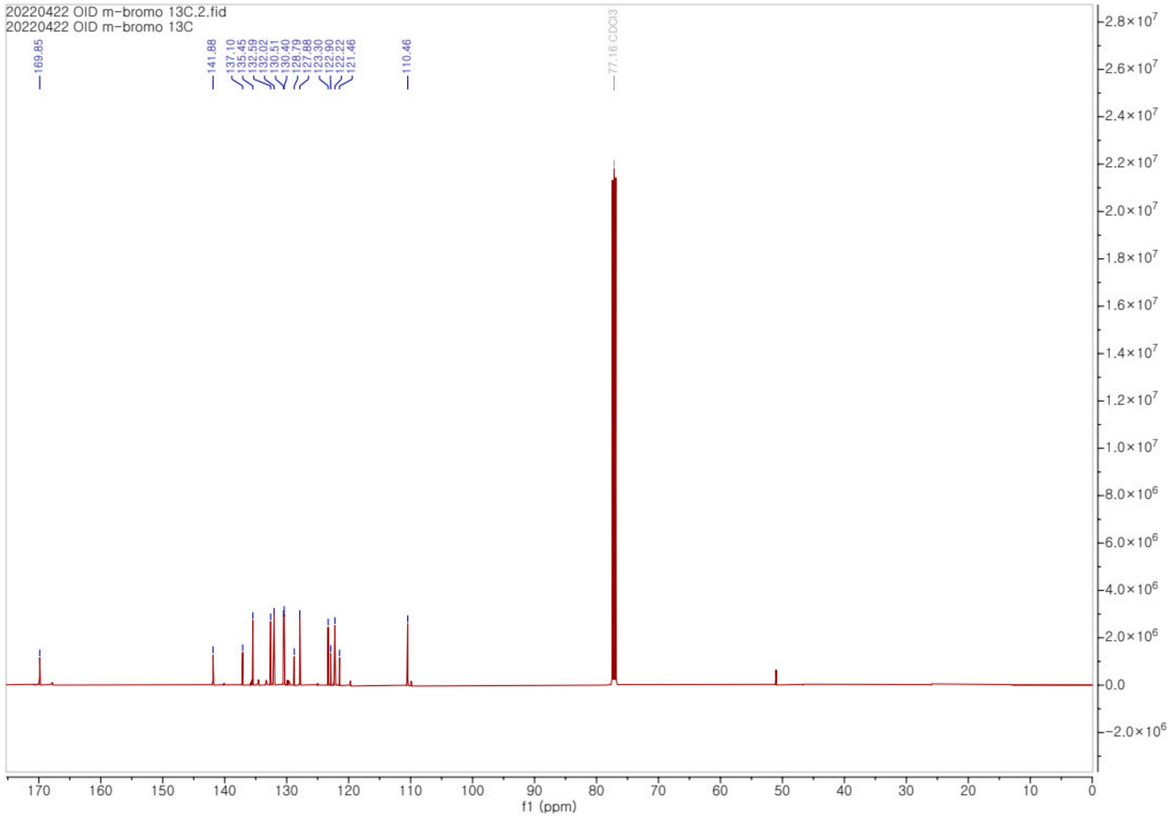

The mass data of OIC14

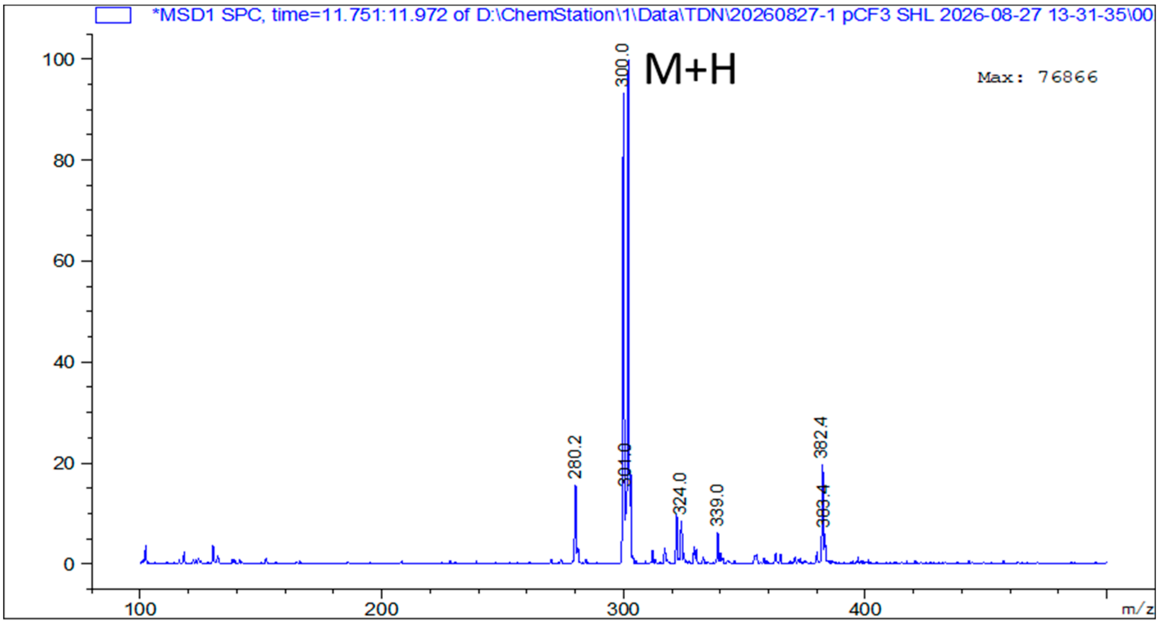

^{1}H-NMR($CDCl_3$) of OIC15

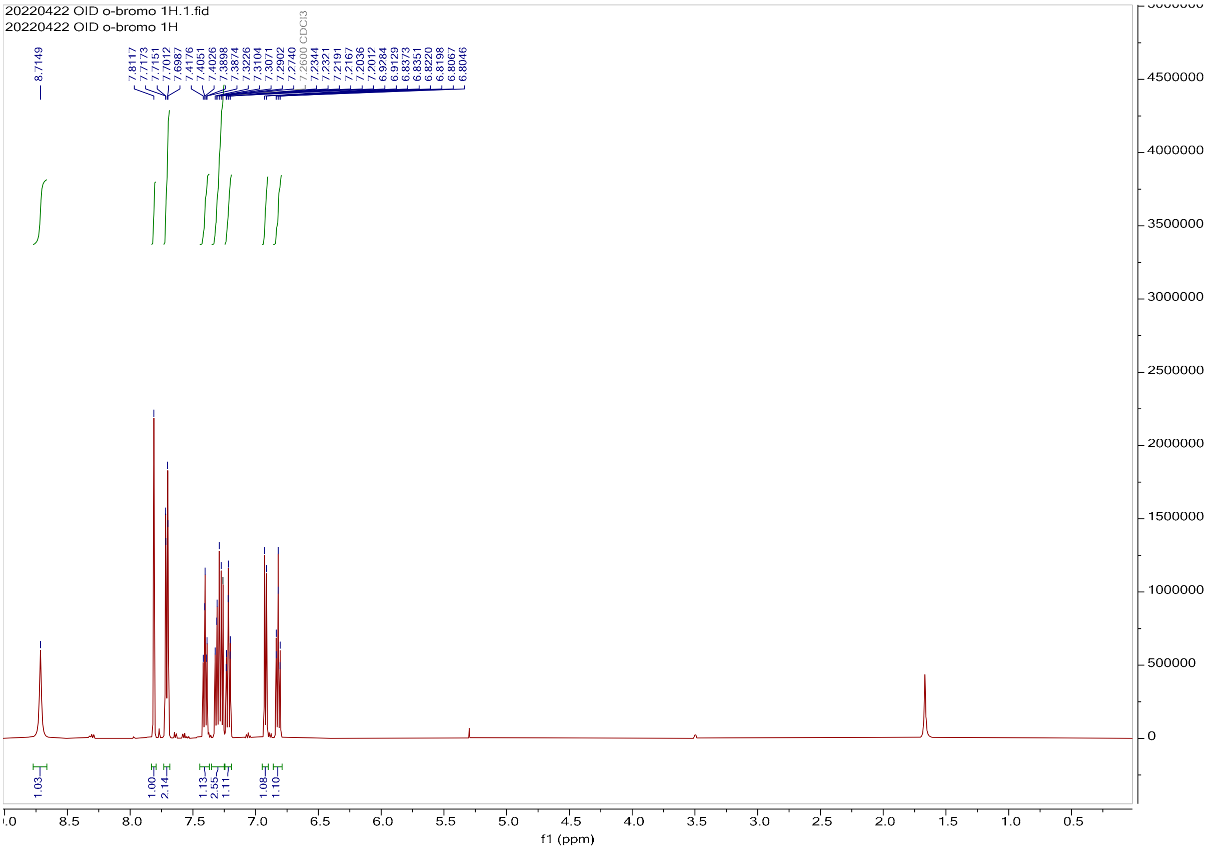

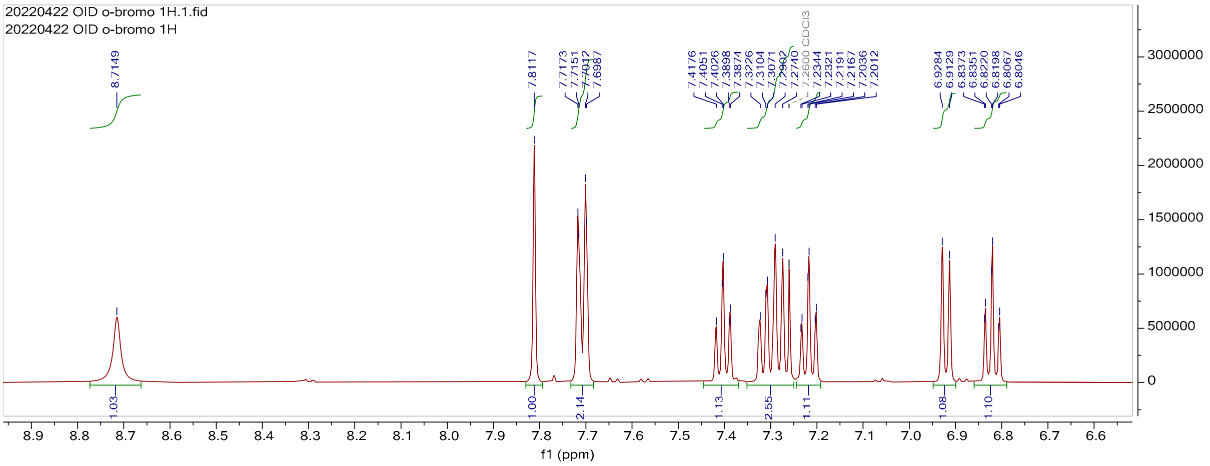

^{13}C-NMR(CDCl$_3$) of OIC15

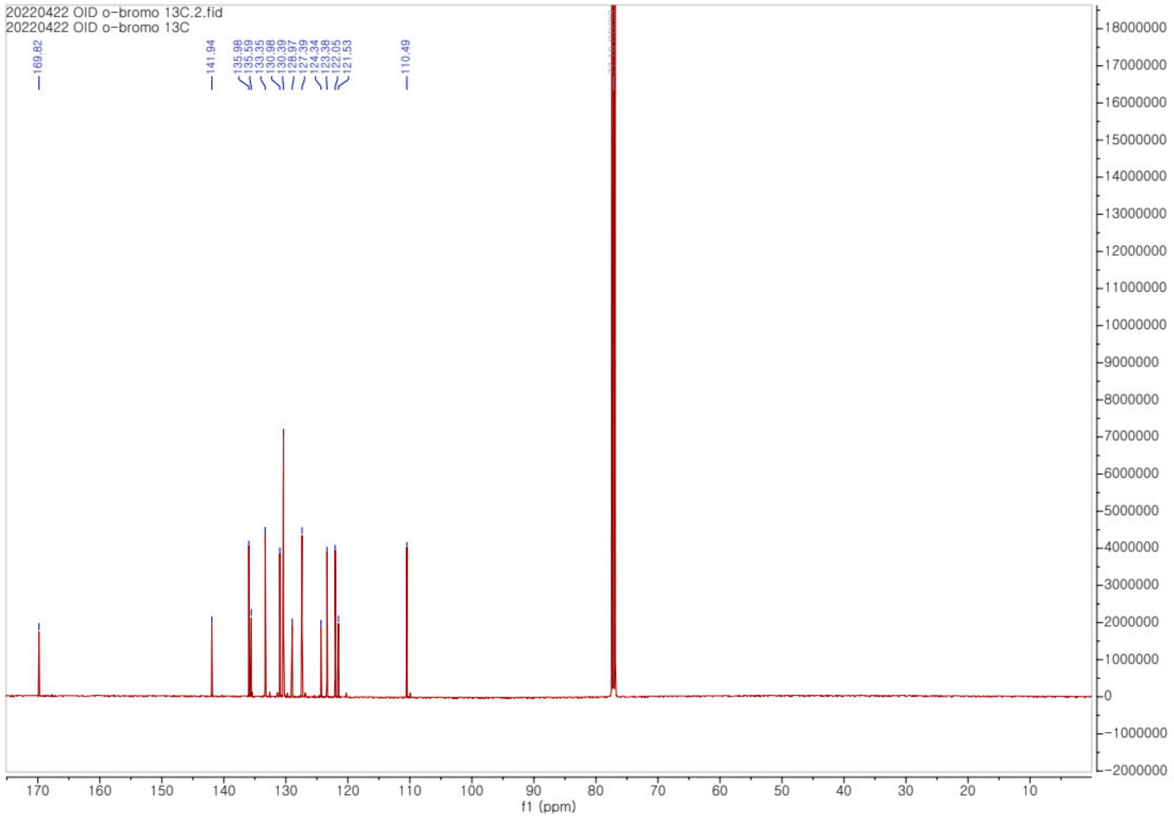

The mass data of OIC15

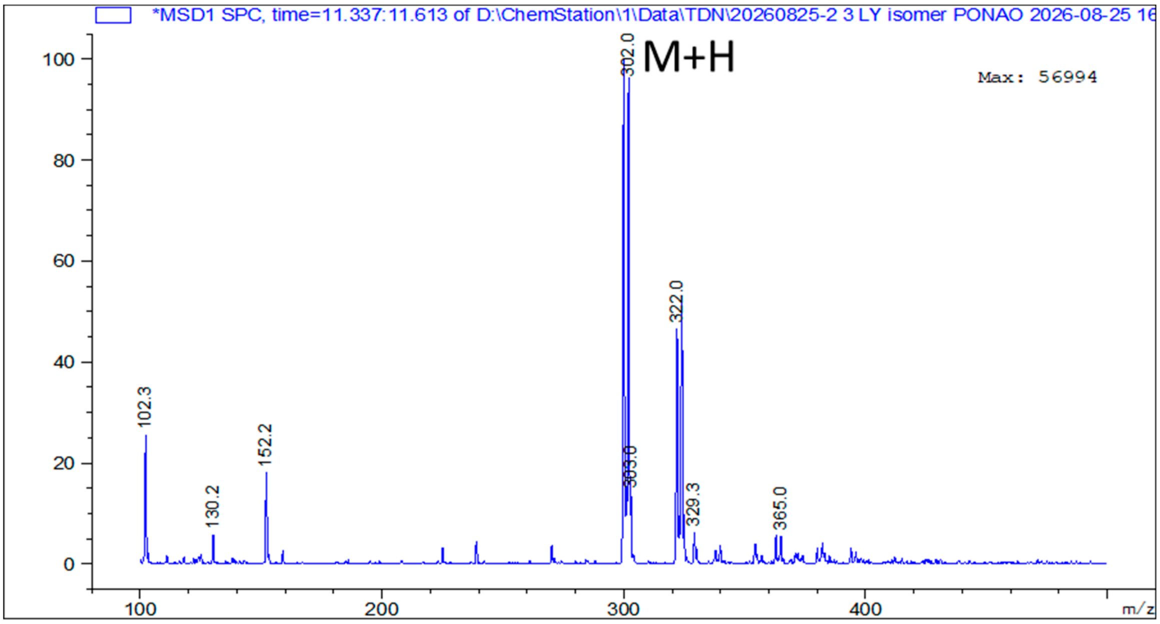

^{1}H-NMR($CDCl_3$) of OIC16

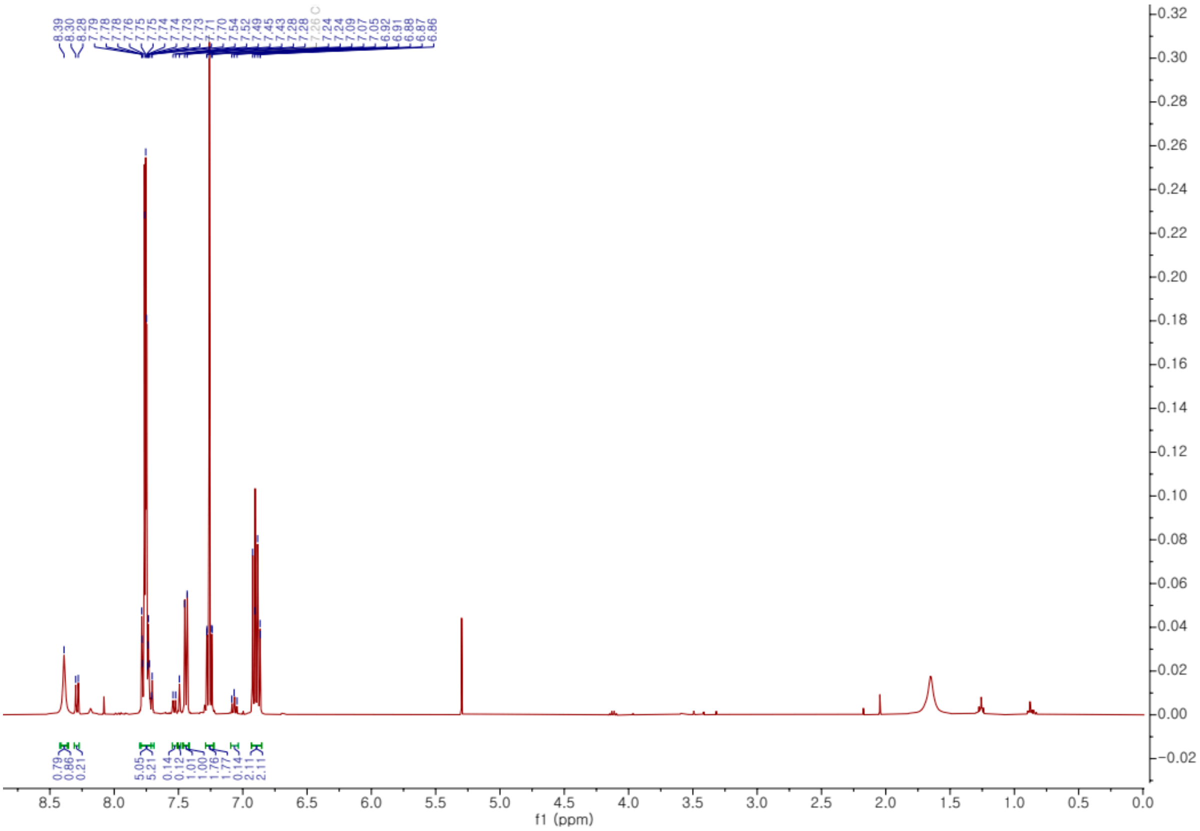

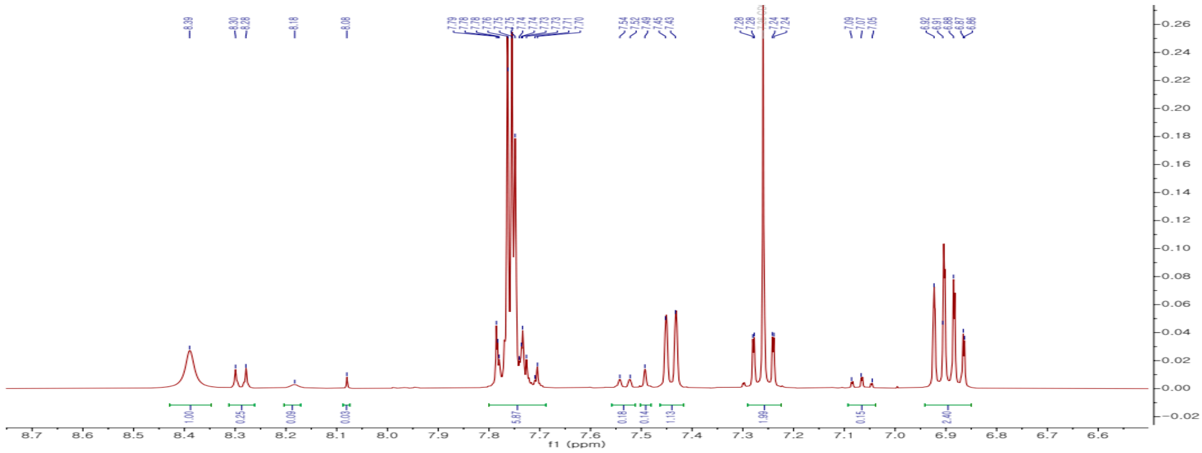

^{13}C-NMR($CDCl_3$) of OIC16

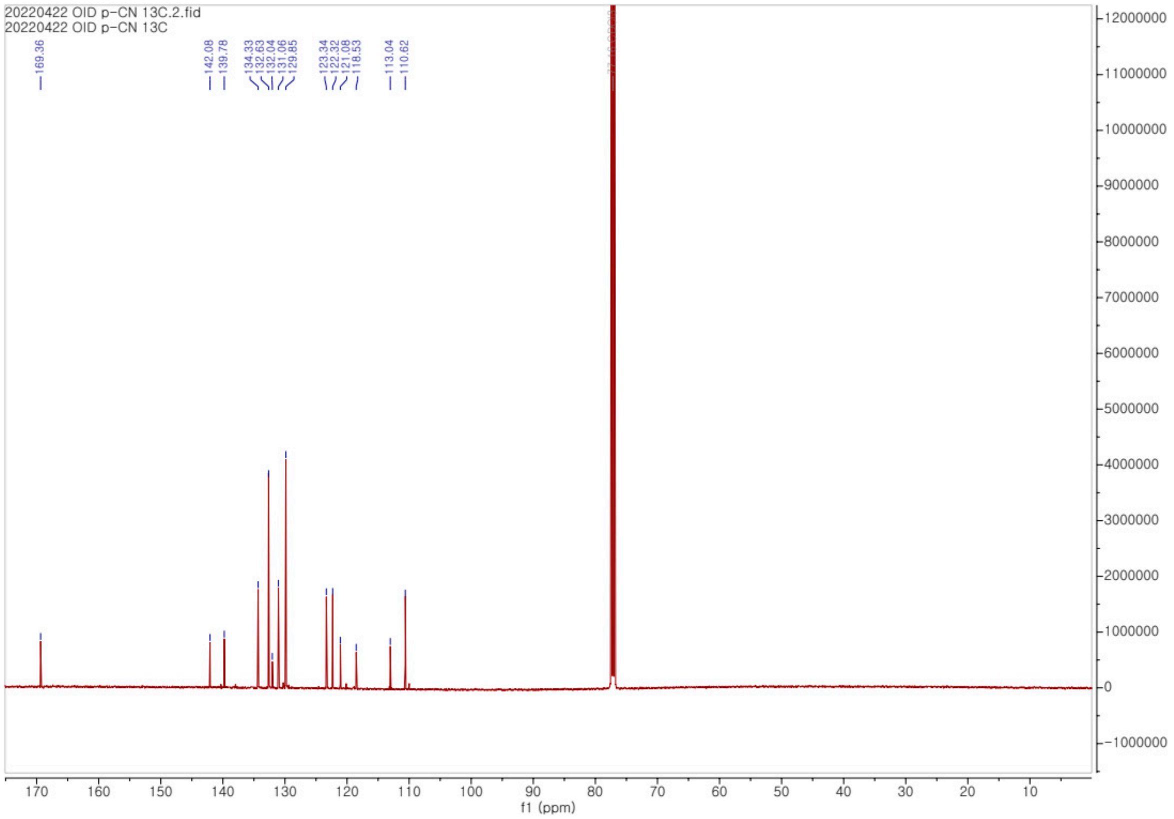

The mass data of OIC16

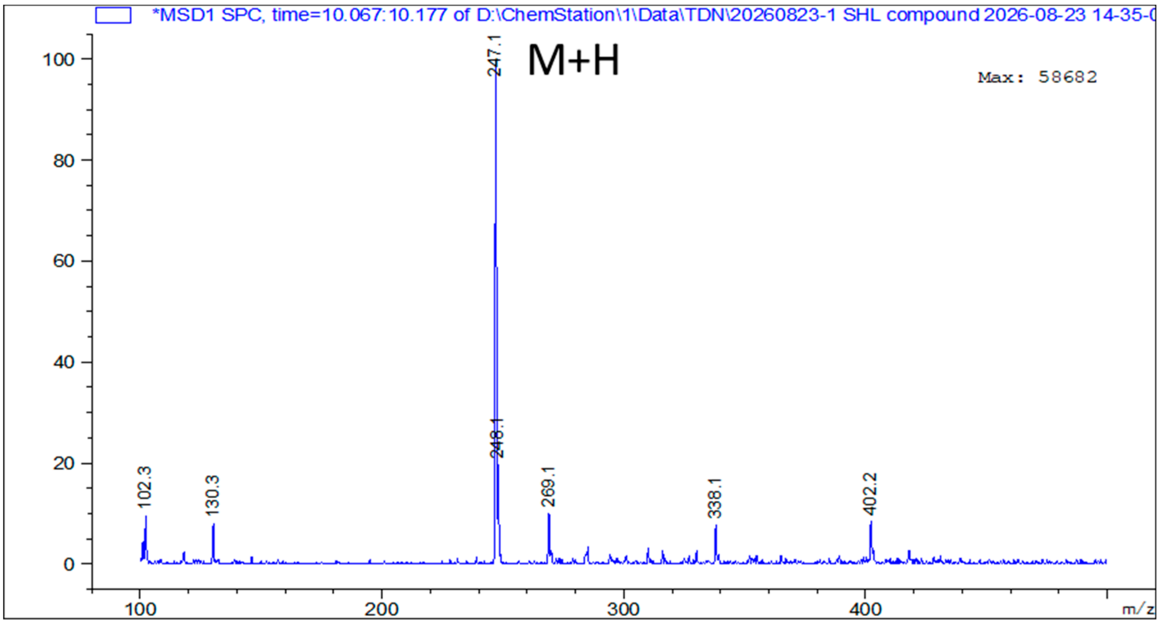

^{1}H-NMR($CDCl_3$) of OIC17

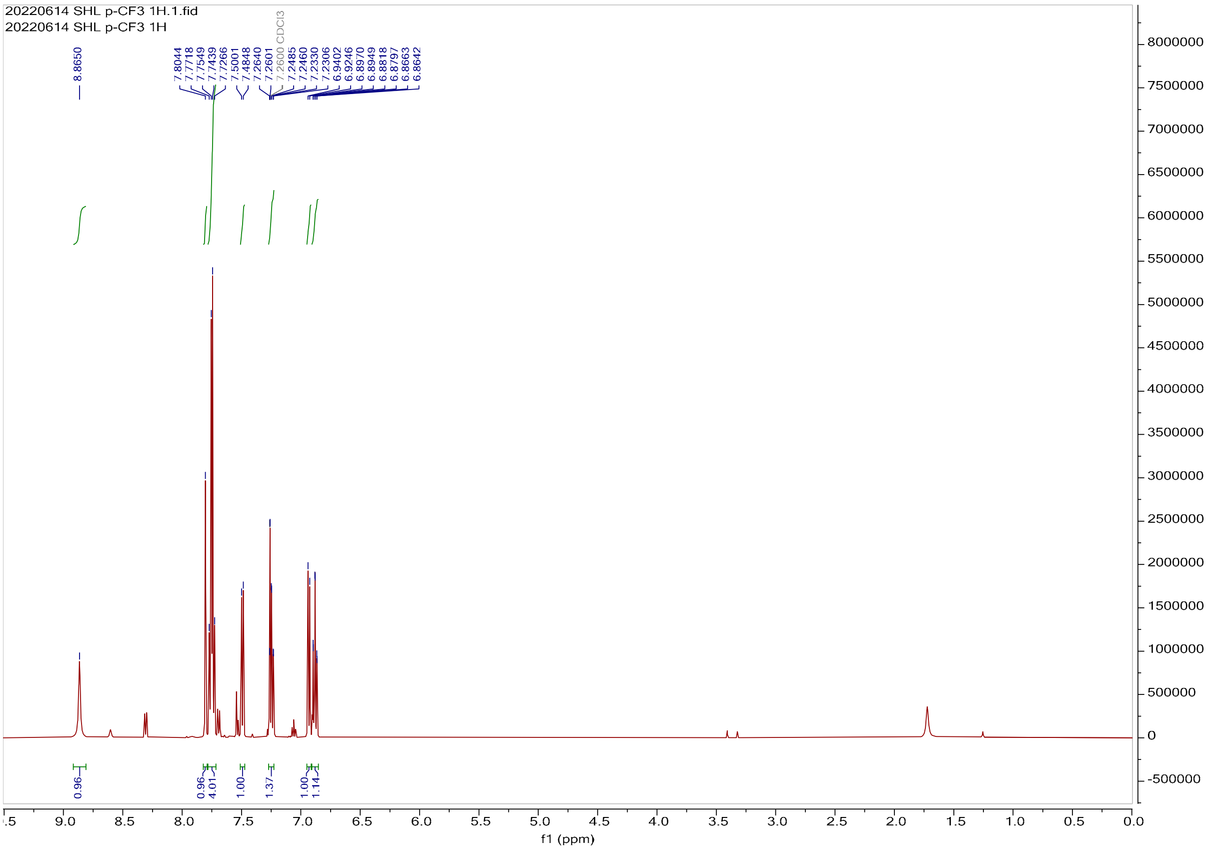

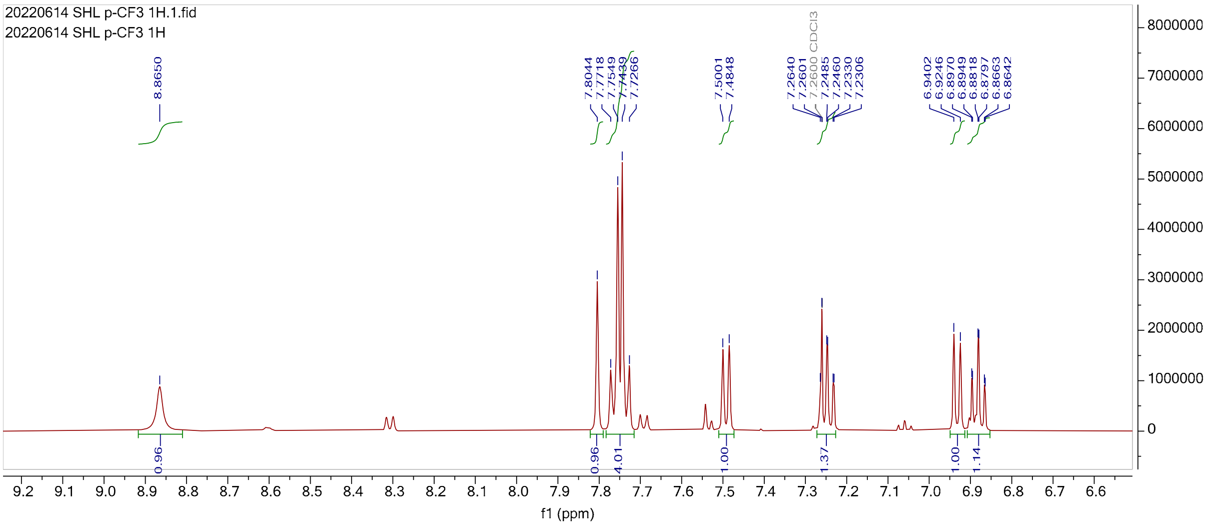

^{13}C-NMR($CDCl_3$) of OIC17

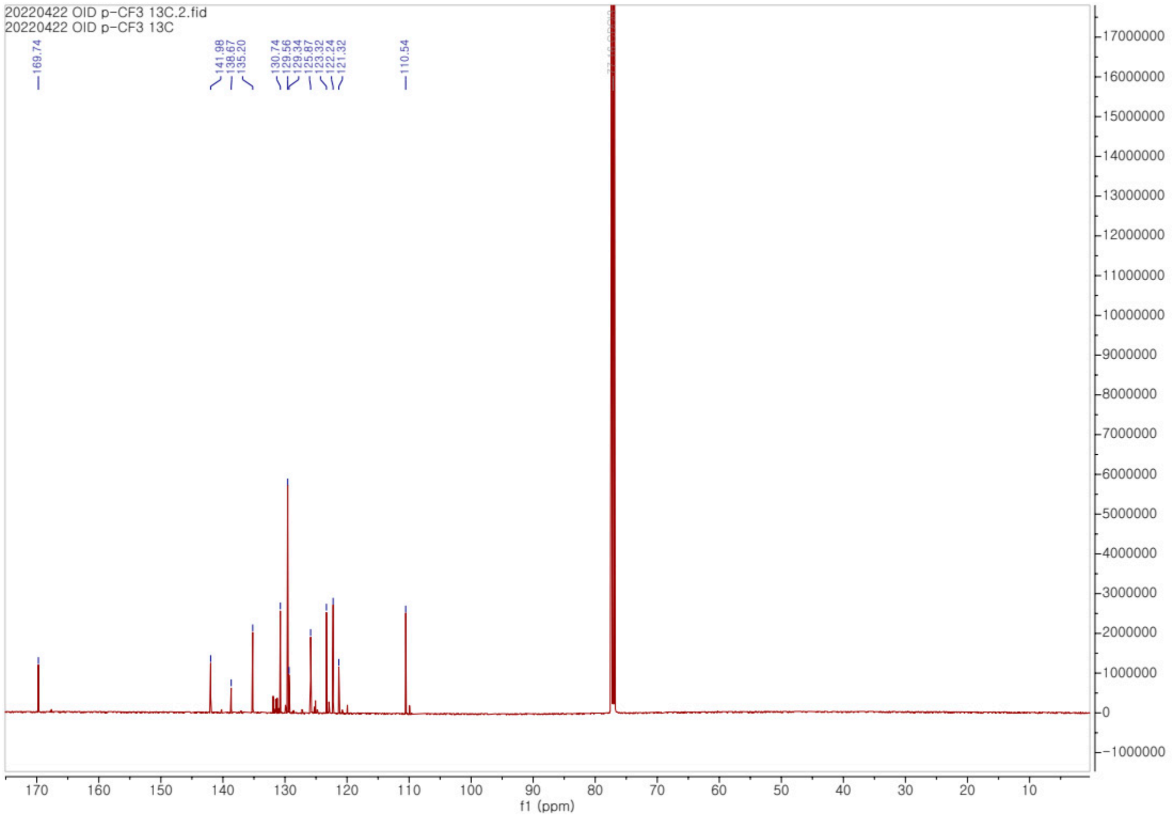

The mass data of OIC17

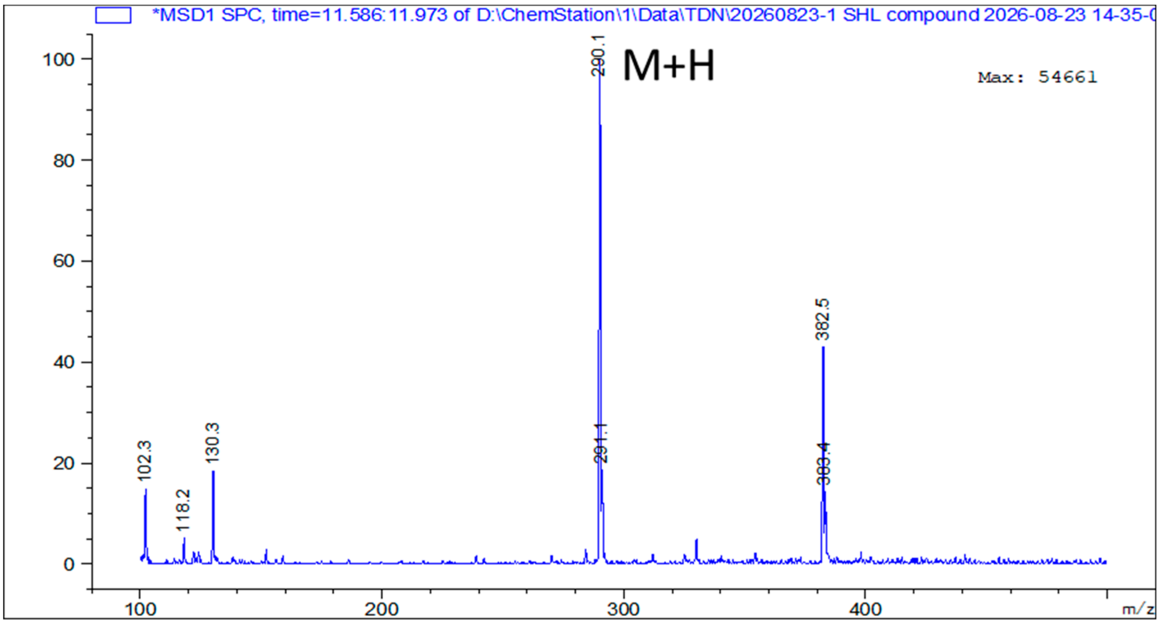

^{1}H-NMR($CDCl_3$) of OIC18

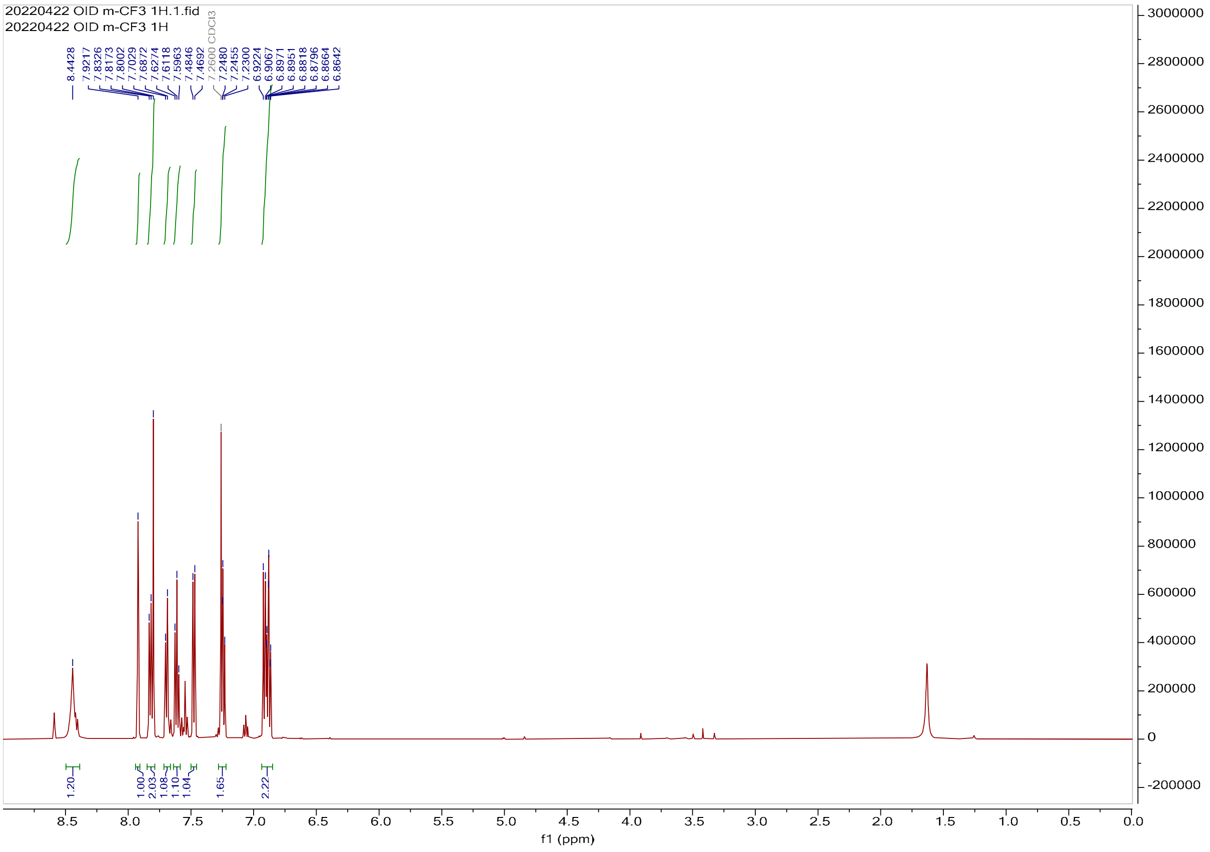

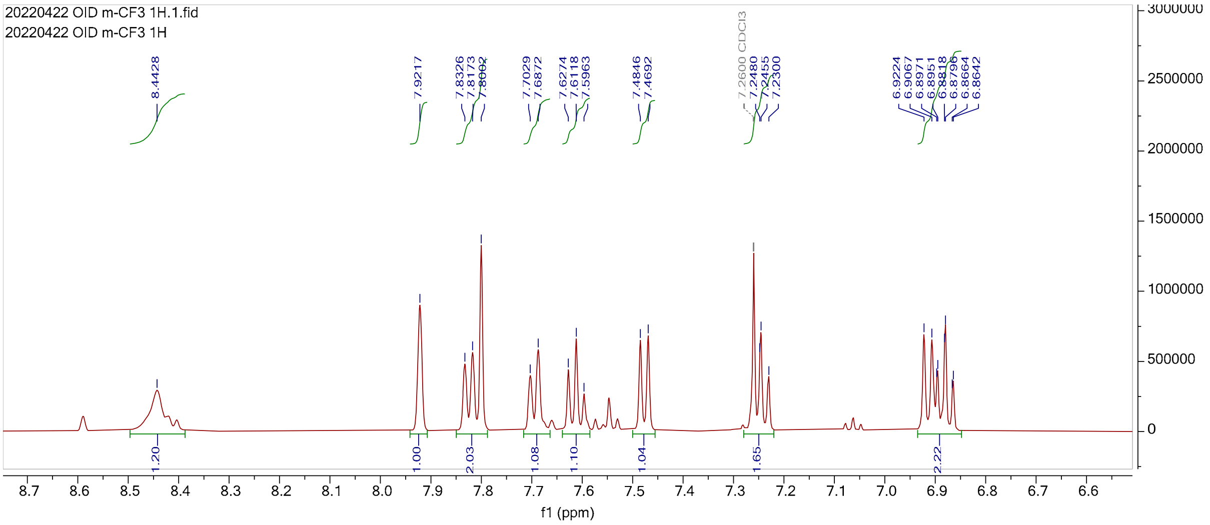

^{13}C-NMR($CDCl_3$) of OIC18

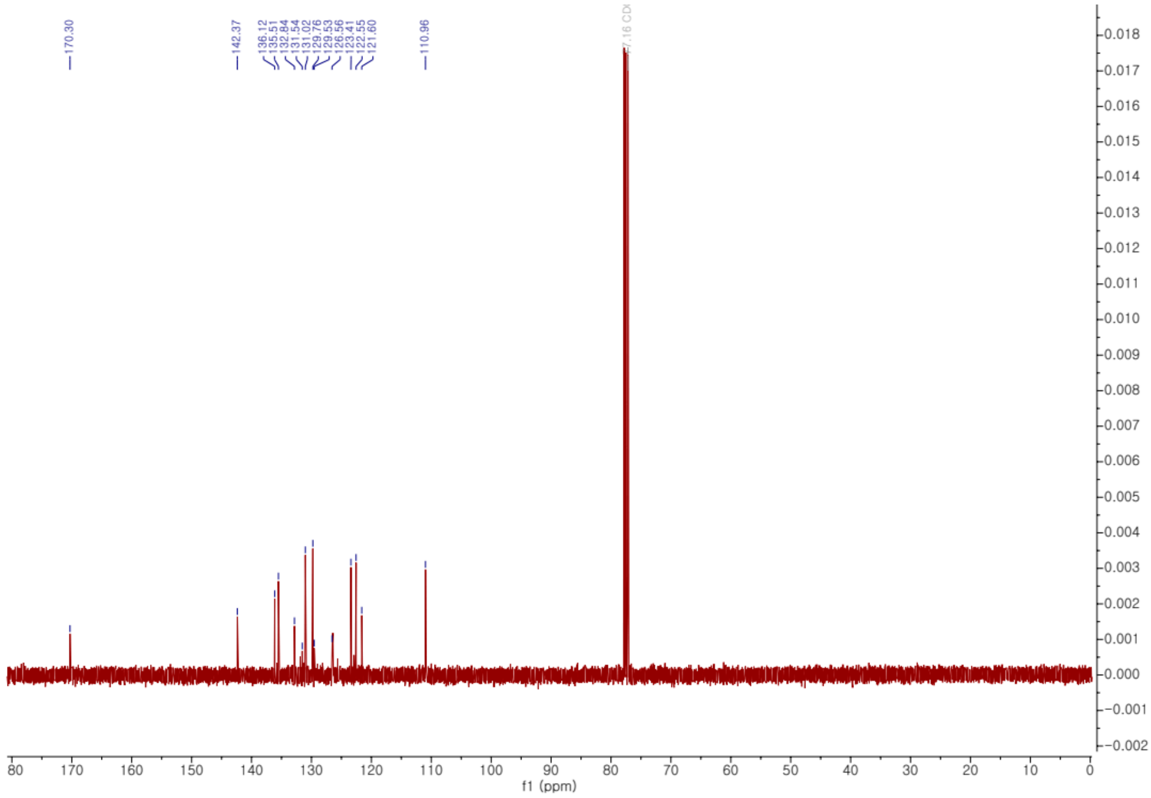

The mass data of OIC18

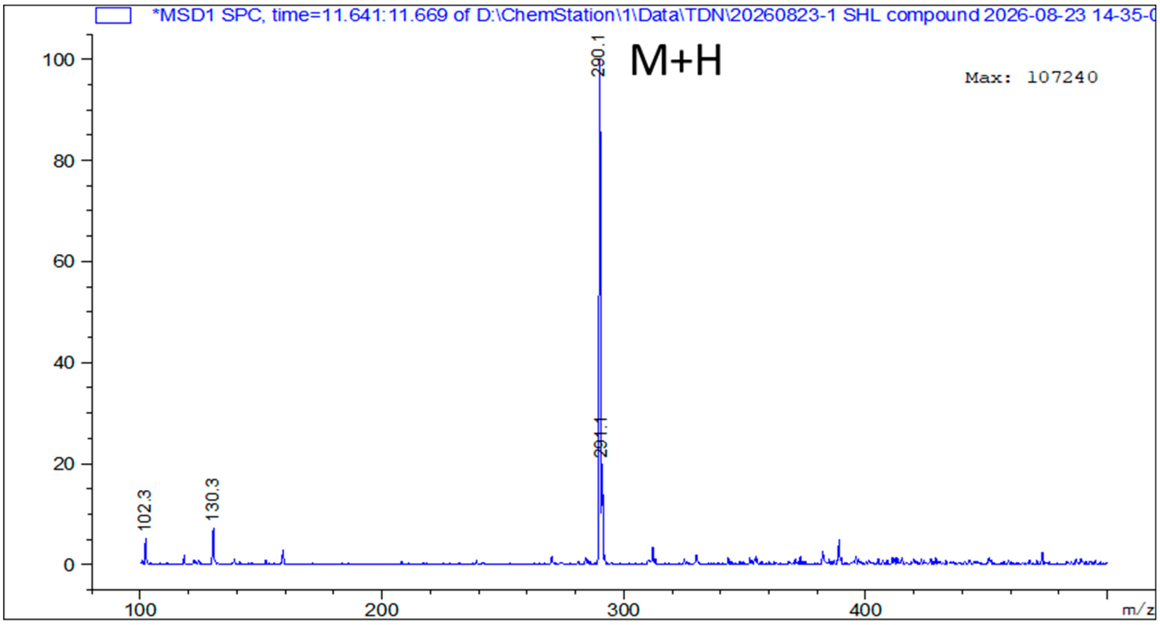

^{1}H-NMR($CDCl_3$) of OIC19

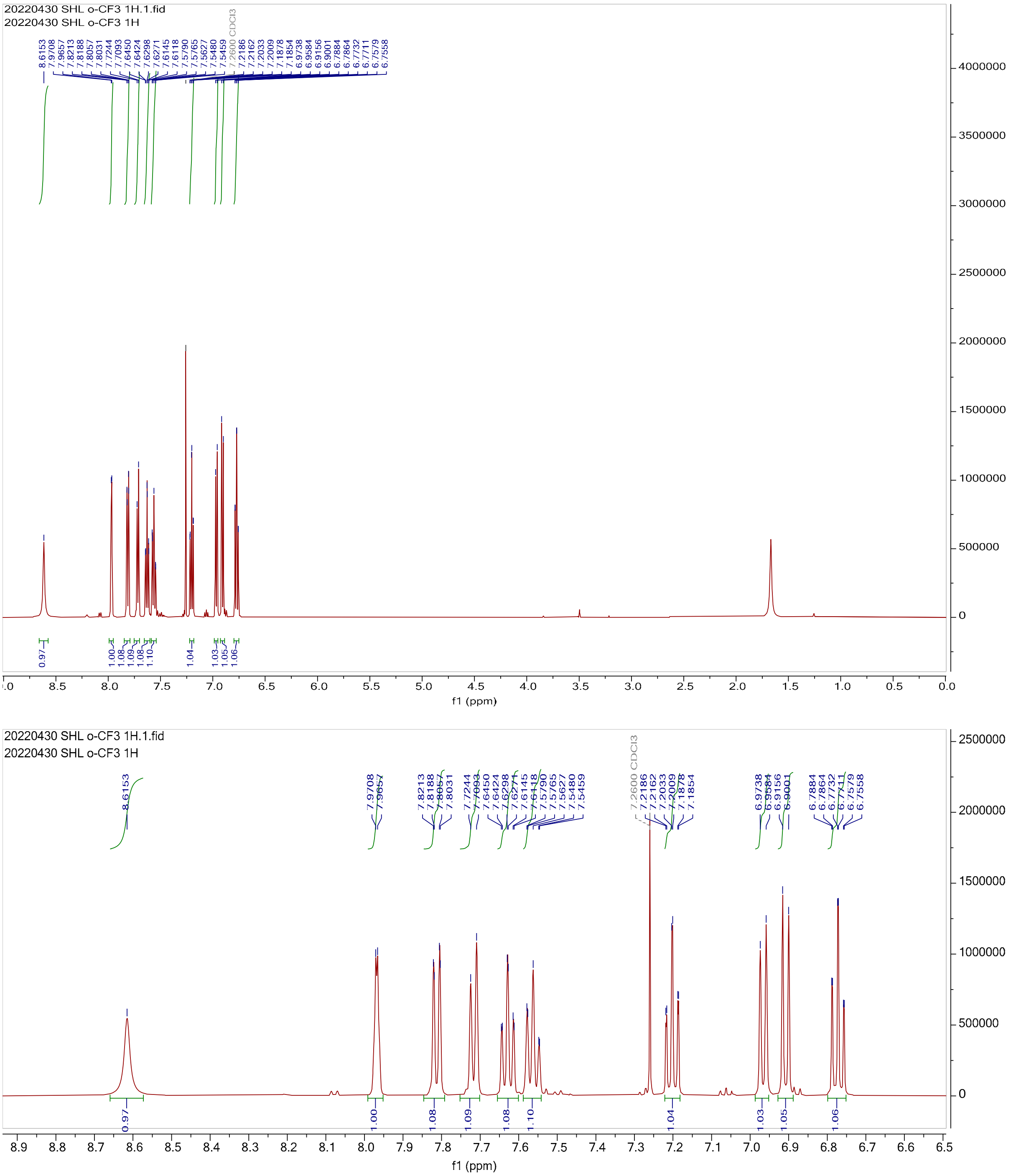

^{13}C-NMR($CDCl_3$) of OIC19

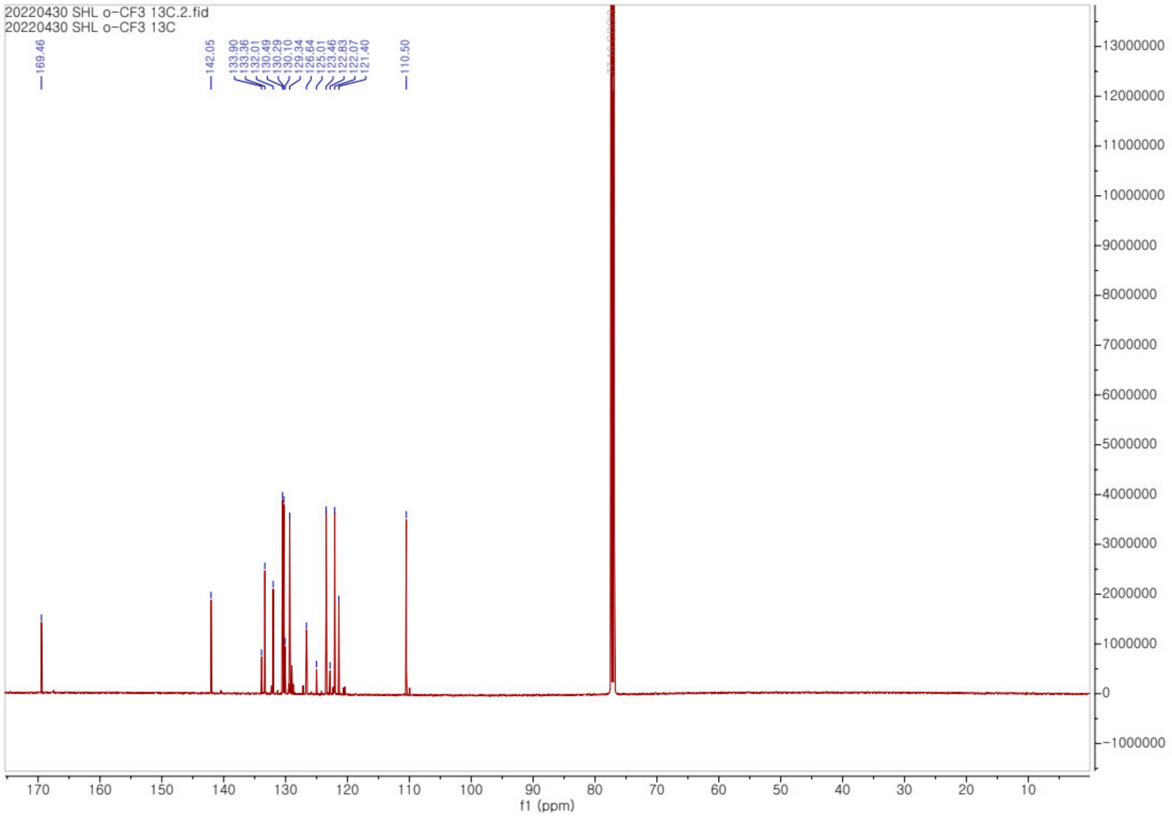

The mass data of OIC19

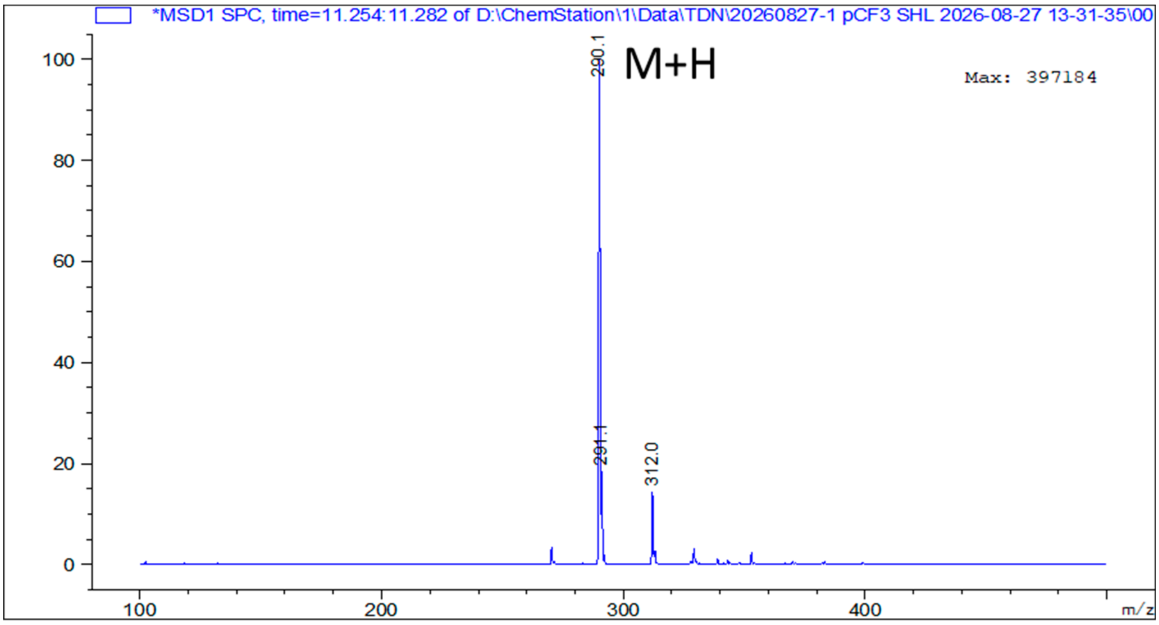

Supplementary Information

Table S1. Quantum chemical descriptors for the oxindole derivatives and reference Michael acceptors.

Compound	Subst.	Position	σ	E_{HOMO} (eV)	E_{LUMO} (eV)	μ (eV)	η (eV)	ω (eV)	Dihedral (°)	f^+ at C_β	f^+ 2nd atom	EC_{50} (μM)
OIC1	Cl	para	0.23	-5.792	-2.150	-3.971	3.642	2.165	33.3	0.244	0.124	58.2
OIC2	Cl	meta	0.37	-5.815	-2.157	-3.986	3.658	2.171	34.0	0.244	0.127	11.6
OIC3	Cl	ortho	n.a.	-5.789	-2.080	-3.935	3.709	2.087	39.8	0.250	0.125	6.2
OIC4	OMe	para	-0.27	-5.450	-1.814	-3.632	3.636	1.814	30.0	0.267	0.114	ND
OIC5	OMe	meta	0.12	-5.621	-1.925	-3.773	3.696	1.925	34.9	0.262	0.121	41.0
OIC6	OMe	ortho	n.a.	-5.475	-1.800	-3.638	3.675	1.800	34.6	0.256	0.115	6.8
OIC7	F	para	0.06	-5.714	-2.020	-3.867	3.694	2.024	32.7	0.261	0.121	ND
OIC8	F	meta	0.34	-5.774	-2.108	-3.941	3.667	2.118	33.6	0.248	0.126	ND
OIC9	F	ortho	n.a.	-5.727	-2.056	-3.891	3.671	2.062	33.8	0.249	0.124	20.0
OIC10	OH	para	-0.37	-5.482	-1.833	-3.657	3.650	1.833	30.0	0.267	0.116	ND
OIC11	OH	meta	0.12	-5.673	-1.981	-3.827	3.692	1.983	33.5	0.259	0.123	37.4
OIC12	OH	ortho	n.a.	-5.788	-2.030	-3.909	3.758	2.033	43.3	0.290	0.119	ND
OIC13	Br	para	0.23	-5.800	-2.172	-3.986	3.628	2.190	33.3	0.242	0.125	27.3
OIC14	Br	meta	0.39	-5.816	-2.177	-3.997	3.638	2.195	32.4	0.240	0.128	9.3
OIC15	Br	ortho	n.a.	-5.799	-2.060	-3.930	3.738	2.065	43.4	0.254	0.125	5.0
OIC16	CN	para	0.66	-6.019	-2.555	-4.287	3.465	2.652	34.6	0.183	0.124	ND
OIC17	CF3	para	0.54	-5.905	-2.305	-4.105	3.600	2.341	32.8	0.224	0.127	26.0
OIC18	CF3	meta	0.43	-5.845	-2.204	-4.025	3.641	2.225	33.0	0.237	0.129	3.6
OIC19	CF3	ortho	n.a.	-5.834	-2.163	-3.998	3.671	2.178	39.9	0.235	0.126	16.7
Methyl vinyl ketone				*-6.728*	*-1.482*	*-4.105*	*5.246*	*1.606*	–	–	–	–
(E)-3-Benzylidene-2-oxindole				*-5.666*	*-1.958*	*-3.812*	*3.708*	*1.959*	*33.7*	*0.258*	–	–
trans-Chalcone				*-6.313*	*-2.095*	*-4.204*	*4.218*	*2.095*	–	–	–	–
Dimethyl fumarate				*-7.631*	*-2.214*	*-4.922*	*5.416*	*2.237*	–	–	–	–

$\mu = (E_{HOMO} + E_{LUMO})/2$; $\eta = E_{LUMO} - E_{HOMO}$; $\omega = \mu^2/(2\eta)$.

Hammett constants from ref. [47]; n.a., not tabulated for *ortho* substituents.

The second-ranked atom by f^+ was the oxindole C3 in every compound.

EC_{50} values are from Table 1; ND, not determined.

All structures were free of imaginary frequencies. Boltzmann-weighted values differed from those listed by less than 0.03 eV, and continued optimization from the converged geometries changed ω by less than 0.012 eV.

Table S2. Covalent docking of OIC3 and OIC15 to Cys151 of the KEAP1 BTB domain (PDB 4CXT).

Parameter	CDDO (redocked)	OIC3	OIC15
cdock affinity (kcal mol^{-1})[a]	−4.87	−4.83	−4.76
Heavy-atom RMSD to the crystallographic pose (Å)	0.53 (0.26)[b]	–	–
RMSD between the two poses, common scaffold (Å)	–	0.006	0.006
C–S bond length (Å)	–	1.83	1.83
π–π with His154: centroid separation (Å)	–	4.36	4.36
π–π with His154: interplanar angle (°)	–	22.3	22.3
Aromatic C–H···O to the Lys131 backbone carbonyl (Å)	–	2.69	2.69
Nearest protein heavy atom to the *ortho* halogen (Å)	–	4.79	4.89

[a] Mean of the pre- and post-reaction GlideScore.

[b] Value in parentheses excludes the two nitrile atoms, whose orientation differs because the deposited ligand is modeled as the enol tautomer whereas the docking protocol generates the keto tautomer.

–, not applicable.
